# Optimal precision and accuracy in 4Pi-STORM using dynamic spline PSF models

**DOI:** 10.1038/s41592-022-01465-8

**Published:** 2022-05-16

**Authors:** Mark Bates, Jan Keller-Findeisen, Adrian Przybylski, Andreas Hüper, Till Stephan, Peter Ilgen, Angel R. Cereceda Delgado, Elisa D’Este, Alexander Egner, Stefan Jakobs, Steffen J. Sahl, Stefan W. Hell

**Affiliations:** 1 Department of NanoBiophotonics, Max Planck Institute for Biophysical Chemistry, Göttingen, Germany; 2 Department of Optical Nanoscopy, Institute for NanoPhotonics, Göttingen, Germany; 3 Clinic of Neurology, University Medical Center Göttingen, Göttingen, Germany; 4 Department of Optical Nanoscopy, Max Planck Institute for Medical Research, Heidelberg, Germany; 5 Optical Microscopy Facility, Max Planck Institute for Medical Research, Heidelberg, Germany

**Keywords:** Super-resolution microscopy, Fluorescence imaging, Single-molecule biophysics

## Abstract

Coherent fluorescence imaging with two objective lenses (4Pi detection) enables single-molecule localization microscopy with sub-10 nm spatial resolution in three dimensions. Despite its outstanding sensitivity, wider application of this technique has been hindered by complex instrumentation and the challenging nature of the data analysis. Here we report the development of a 4Pi-STORM microscope, which obtains optimal resolution and accuracy by modeling the 4Pi point spread function (PSF) dynamically while also using a simpler optical design. Dynamic spline PSF models incorporate fluctuations in the modulation phase of the experimentally determined PSF, capturing the temporal evolution of the optical system. Our method reaches the theoretical limits for precision and minimizes phase-wrapping artifacts by making full use of the information content of the data. 4Pi-STORM achieves a near-isotropic three-dimensional localization precision of 2–3 nm, and we demonstrate its capabilities by investigating protein and nucleic acid organization in primary neurons and mammalian mitochondria.

## Abstract (web summary)

A dynamic model of the 4Pi point spread function enables localization microscopy with exceptional three-dimensional resolution and a simpler optical design. 4Pi-STORM images of neurons and mitochondria reveal new details of nanoscale protein and nucleic acid organization.

## Main

Super-resolution microscopy allows biological samples to be visualized in new ways, by bringing together the benefits of fluorescent labeling with a resolving power far beyond the classical diffraction limit^1–3^. In particular, optical designs incorporating two objective lenses that detect light coherently (4Pi detection)^4–6^ are well suited for three-dimensional (3D) visualization of transparent samples, maximizing photon collection efficiency and resolution along the optical axis. The high axial sensitivity has allowed single-molecule localization microscopy (SMLM) concepts such as PALM and STORM^7–9^, when implemented with 4Pi detection, to achieve near-isotropic spatial resolution of ~10 nm^10–13^. Significantly, the ability to resolve 3D structure at biomolecular length scales has enabled the application of 4Pi-SMLM in studies of nanoscale protein localization^14,15^.

The advantages of 4Pi microscopy come at a cost, however. With a design similar to an interferometer, the microscope is sensitive to drift and other optical perturbations. Furthermore, the periodicity of the 4Pi point spread function (PSF) leads to the possibility of phase-wrapping errors during data analysis, which appear as periodic ‘ghost image’ artifacts. Depending on the sample, such artifacts can make it difficult to interpret 4Pi-SMLM images, or restrict the imaging depth to a thin section of only a few hundred nanometers in the *z* dimension. This problem can be mitigated by the addition of astigmatism to the PSF^13,16^, albeit at the expense of decreased resolution and higher instrument complexity.

In this work, we introduce a model of the 4Pi PSF that accurately captures the full PSF structure while allowing temporal variations in the phase of the PSF modulation to be detected and compensated. This model is an essential element of the 4Pi-STORM microscope presented here, which achieves optimal localization precision and artifact-free imaging over a large sample depth, without relying on modifications to the PSF such as astigmatism. We describe a simpler and more accessible optical design with substantially improved performance. We illustrate our method with single-color and multicolor nanoscale 3D imaging of nuclear pore complexes (NPCs), the neuronal cytoskeleton, and mitochondria, and we highlight new analyses made possible by the spatial resolution of each dataset.

## Results

### Numerical representation of the 4Pi point spread function

**Fig. 1:**
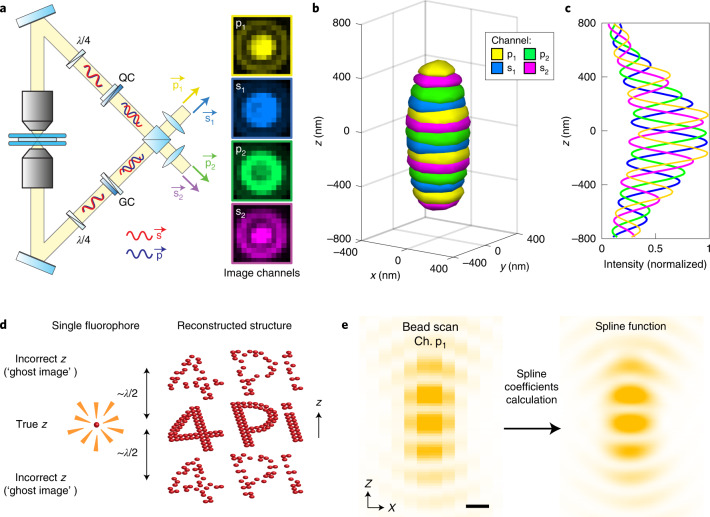
Cubic spline model of the 4Pi point spread function. **a**, Schematic of the 4Pi interferometric cavity. Light from the sample is collected by two objective lenses, and the p-polarized fluorescence is delayed relative to the s-polarized fluorescence by modified Babinet–Soleil compensators (QC and GC). The photons self-interfere at the 50:50 beam splitter, resulting in four beams (p_1_, s_1_, p_2_ and s_2_), each with a distinct interference phase. Images of a fluorescent emitter exhibit different patterns in each of the four detection channels (right). Quarter-wave plates, quartz wedge compensator, and glass wedge compensator are labeled by *λ*/4, QC, and GC, respectively. **b**, Four-channel representation of the microscope PSF, rendered as a 3D isosurface (at 35% of maximum intensity). **c**, Central intensity profile of the PSF, measured along the *z* axis, showing the modulation of each channel as the detected signals oscillate between constructive and destructive interference. **d**, Schematic of ghost image artifacts, arising due to localization errors that occur when an emitter’s *z* coordinate is assigned to the wrong interference period, shifting its position by a multiple of the fringe spacing (approximately *λ*/2). **e**, A cross-sectional slice (*x*–*z*) through the center of the pixelated PSF measurement (left), and the corresponding slice through the PSF spline function (right), rendered with a 20-fold higher sampling density, which captures the full detail of the PSF structure. For clarity, only channel p_1_ is shown. Scale bar, 250 nm.

**Extended Data Fig. 1:**
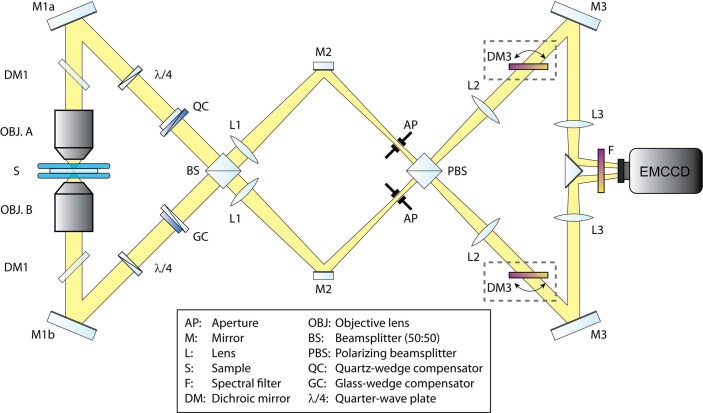
4Pi-STORM detection path. Optical layout of the 4Pi-STORM interferometric cavity and fluorescence detection path. Fluorescence emitted by the sample (S) is collected by the two objective lenses (OBJ. A and B) and travels along the arms of the cavity passing through the dichroic mirrors (DM1), quarter wave plates (λ/4), and Babinet-Soleil compensators (QC and GC). The fluorescence photons meet and self-interfere at the 50:50 beam-splitter cube (BS). Tube lenses (L1) form an image of the sample at the apertures (AP). After passing through the polarizing beam-splitter cube (PBS), the S-polarized fluorescence is separated from the P-polarized fluorescence. The telescopes formed by lenses L2 and L3 create four images of the sample on four quadrants of the EMCCD camera. The fluorescence signal is filtered by spectral filters placed before the camera (F). In the multicolor detection configuration, dichroic mirrors DM3 are placed in the detection path after lenses L2. These dichroic mirrors act as polarization sensitive long-pass spectral filters, and are mounted on rotation mounts so that their angle can be precisely adjusted to set the cutoff wavelength of the filter.

The 4Pi-STORM setup was designed for improved stability and throughput compared to earlier implementations, for robust and routine imaging of biological samples. Details of the microscope design are given in the Methods (Extended Data Fig. 1 and Supplementary Figs. 1–8). The microscope uses an optical layout in which fluorescence from the sample is collected coherently by two objective lenses, and divided by polarization and phase to be detected as four interference image channels (p_1_, s_1_, p_2_, and s_2_) on an EMCCD camera (Fig. 1a). Each channel corresponds to a specific phase delay (0, π/2, π, and 3π/2) between the two arms of the optical cavity. In combination, the images may be used to determine an interference phase for each single-fluorophore detection event, which is equivalent to a sensitive measurement of the emitter’s *z* coordinate (modulo the interference fringe period)^10–12^.

The PSF of the microscope was measured by scanning a small fluorescent bead through the focal plane, which serves as a representation of a freely rotating fluorophore because the bead contains dye molecules with all orientations. The resulting intensity distribution is displayed as a 3D isosurface in Fig. 1b, with the four channels rendered in different colors (Supplementary Fig. 9). A central line profile through the PSF (Fig. 1c) reveals the periodic modulation of the fluorescence along the *z* axis, as each channel oscillates between constructive and destructive interference.

This sharp periodicity is the source of the microscope’s sensitivity^10^, but it can also result in image artifacts. Estimation of the phase of the modulation is not sufficient to unambiguously determine an emitter’s *z* coordinate, because the phase repeats for each interference fringe, with a period of approximately one half of the fluorescence wavelength (~*λ*/2). Errors in assigning each localization to the correct fringe of the modulation pattern result in ghost images that repeat along the *z* direction, as illustrated in Fig. 1d, obscuring the true structure of the specimen.

Recently, a new method was proposed for fluorophore localization in SMLM data analysis, in which an analytic model of the PSF, in the form of a cubic spline, is created from an experimental measurement^17,18^. We hypothesized that this approach would be particularly effective for 4Pi-STORM data analysis. As opposed to models that account for only the PSF’s phase^11^ or incorporate approximate shape measures^12,13^, a spline model is capable of fully capturing the complex structural detail of the 4Pi PSF, including asymmetric components that reduce the self-similarity in different *z* planes.

To create an initial PSF model, we determined the piecewise-continuous 3D cubic polynomials that describe the PSF measurement. The static spline model of the PSF (*h*_4Pi_) is given by equations (1) and (2):

1 $$h_{{{{\mathrm{4Pi}}}}}\left( {x,y,z,c{{{\mathrm{|}}}}x_0,y_0,z_0,A,b} \right) = b + Af_S(x - x_0,y - y_0,z - z_0,c),$$

2 $$\begin{array}{c}f_S\left( {x,y,z,c} \right) = \mathop {\sum }\limits_{m = 0}^3 \mathop {\sum }\limits_{n = 0}^3 \mathop {\sum }\limits_{o = 0}^3 S_{i,j,k,m,n,o,c}( {\frac{{x - t_i}}{{\Delta t}}} )^m( {\frac{{y - u_{\small j}}}{{\Delta u}}} )^n( {\frac{{z - v_k}}{{\Delta v}}} )^o,\\ \left(\small {\begin{array}{*{20}{c}} {t_i \le x \le t_{i + 1},\, u_j \le y \le u_{j + 1},\, v_k \le z \le v_{k + 1}} \\ {\Delta t = t_{i + 1} - t_i,\,\Delta u = u_{j + 1} - u_j,\,\Delta v = v_{k + 1} - v_k} \end{array}} \right)\end{array}$$

where $(x_0,y_0,z_0)$ is the PSF center coordinate, *A* is the amplitude, and *b* is the background offset. Here the multichannel 3D spline function is denoted *f*_*S*_, where (*i*, *j*, *k*) are volume interval indices, $(t_i,u_j,v_k)$ are the interval center coordinates, *c* is the channel index and $S_{i,j,k,m,n,o,c}$ are the spline coefficients. The coefficients *S* were computed directly from the pixelated bead scan data (Methods). A cross-section through the center of the scan is plotted in Fig. 1e, where only one channel (p_1_) has been shown for clarity. The corresponding spline function (Fig. 1e) is smooth, reproducing the measured PSF, including the interference modulation (Supplementary Fig. 10). Conceptually, the spline model is sufficient to fully describe the detected signal from a fluorophore at any position in the sample.

The simple analytic form of the model lends itself well to numerical optimization, but presents a computational bottleneck owing to the large number of terms in the polynomials and their derivatives. To avoid this issue, we implemented multichannel 3D spline functions in Gpufit, an open-source graphical processing unit (GPU)-accelerated curve fitting library^19^. Using this approach, we were able to rapidly estimate the spatial coordinates of single emitters by fitting the fluorophore images with the spline function derived from the PSF measurement (Methods).

### Dynamic spline point spread function model

While the 4Pi interferometric detection concept has advantages in terms of precision, it is highly sensitive to perturbations in the optical cavity, such as thermal drift. A degree of cavity drift is unavoidable, and its effect on the PSF is to cause a shift in the phase of the interference modulation.

**Fig. 2:**
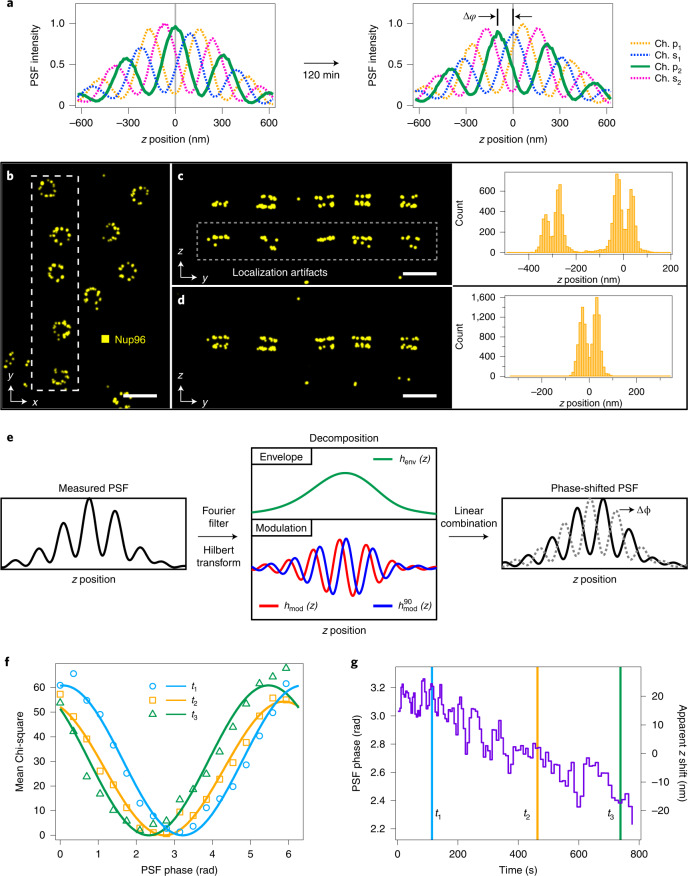
Point spread function phase estimation and correction. **a**, Central profile of the 4Pi PSF at two time points during an experiment, showing a shift in the phase of the modulation (Δ*φ*) relative to the focal plane (*z* = 0). **b**, 4Pi-STORM image of NPCs, in which SNAP-tagged Nup96 is labeled with Alexa Fluor 647. Top view, *x*–*y* projection. **c**, Side view (*y*–*z* projection) of five nuclear pores shown in the boxed region in **b**, processed without phase correction. The image exhibits strong ghost artifacts of the Nup96 structure (gray box). A histogram of the *z* coordinates of the localizations (right) also shows the artifacts, separated by approximately 300 nm from the true signal. **d**, The same region shown in **c**, after fitting the data with a phase-corrected PSF model. The ghost images are no longer present, and the *z-*profile histogram (right) shows only two peaks corresponding to the expected Nup96 distribution in the nuclear membrane. Scale bars, 250 nm. **e**, Illustration of the phase-shift transform. Beginning with the original PSF scan (one channel shown), the PSF is decomposed into *h*_env_ and *h*_mod_ components. A Hilbert transform is used to obtain a 90°-shifted modulation component ($h_{{{{\mathrm{mod}}}}}^{90}$). The components are linearly combined with a phase factor Δ*φ*, to obtain the 4Pi PSF with an arbitrary phase shift. **f**, Measurement of the PSF phase. Three sets of localizations from narrow time windows (*t*_1_, *t*_2_, and *t*_3_) were fit with PSF models having a range of phases spanning 360°, and the mean Chi-square value for each set of fit results was plotted versus the model phase. The plots exhibit a single minimum at the true phase of the PSF in that time window. Solid lines show sinusoidal fits to the data. **g**, Evolution of the PSF phase. Repeating the analysis shown in **f** for the entire Nup96 dataset yielded the time evolution of the PSF phase during the experiment. The three time points from **f** are indicated on the plot. In this example, each time window corresponds to 250 localizations and the phase measurement precision was 0.03 radians (Supplementary Fig. 14).

We tested for phase drift in our system by measuring the PSF at different time points. Two bead scans recorded 120 min apart showed a clear shift in the modulation phase with respect to the center of focus (Fig. 2a) despite the optical system being fully enclosed and temperature regulated. This result suggests a potential problem for the spline-based PSF model: if the phase changes after the PSF is recorded, the molecule images measured during the STORM experiment will no longer be well described by the model, leading to inaccurate fit results.

The effects of PSF phase drift can be seen in 4Pi-STORM image data. Figure 2b shows a 4Pi-STORM image of NPCs, in which the nucleoporin Nup96 was labeled with Alexa Fluor 647. Initially, this dataset was analyzed using a cubic spline PSF model based on the bead scan recorded at the start of the experiment. In the top view (*x*–*y* projection), the pores appear sharp and well resolved (Fig. 2b). A side view (*y*–*z* projection) of the same region, however, shows a large fraction of localizations incorrectly assigned by the fit procedure, resulting in a strong double image artifact (Fig. 2c, left). The upper and lower Nup96 rings of the NPC appear twice, as shown by a histogram of the localization *z* coordinates (Fig. 2c, right). Using simulations and experiments, we verified that such localization artifacts increase strongly in frequency when the PSF model phase is shifted by more than 60° from the true phase of the PSF (Supplementary Figs. 11 and 12 and Supplementary Note 1).

To overcome this problem, the time dependence of the 4Pi optical system must be accounted for during data analysis. For this purpose, we implemented a method for numerically shifting the phase of the experimentally measured PSF, as illustrated schematically in Fig. 2e. First, using a Fourier low pass filter applied along the *z* dimension, the PSF was decomposed into a static envelope function (*h*_env_) and a modulation component (*h*_mod_). Next, a Hilbert transform was used to alter the phase of the modulation component by 90° ($h_{{{{\mathrm{mod}}}}}^{90}$). These three components, given by equations (3)–(5), were then recombined, while introducing a phase shift Δ*φ*, to yield a new PSF model with an arbitrary modulation phase, according to equation (6).

3 $$h_{{{{\mathrm{env}}}}}\left( {x,y,z,c} \right) = {{{\mathcal{F}}}}_z^{{{{\mathrm{low}}}}}\left[ {h_{{{{\mathrm{4Pi}}}}}\left( {x,y,z,c} \right)} \right]$$

4 $$h_{{{{\mathrm{mod}}}}}\left( {x,y,z,c} \right) = h_{{{{\mathrm{4Pi}}}}}\left( {x,y,z,c} \right) - h_{{{{\mathrm{env}}}}}\left( {x,y,z,c} \right)$$

5 $$h_{{{{\mathrm{mod}}}}}^{90}\left( {x,y,z,c} \right) = {{{\mathrm{real}}}}\left\{ {{{{\mathcal{H}}}}_z\left[ {h_{{{{\mathrm{mod}}}}}\left( {x,y,z,c} \right)} \right]} \right\}$$

6 $$\begin{array}{rl}h_{{{{\mathrm{4Pi}}}}}^{{{{\mathrm{dyn}}}}}\left( {x,y,z,c,\Delta \varphi } \right) =& h_{{{{\mathrm{env}}}}}\left( {x,y,z,c} \right) +\\& \left[ {\begin{array}{*{20}{c}} {\cos \Delta \varphi } & {\sin \Delta \varphi } \end{array}} \right] \cdot \left[ {\begin{array}{*{20}{c}} {h_{{{{\mathrm{mod}}}}}} \\ {h_{{{{\mathrm{mod}}}}}^{90}} \end{array}} \right](x,y,z,c)\end{array}$$

This approach makes no assumptions about the PSF structure, and can be applied to any PSF having a periodic component along the *z* axis. We verified that our algorithm produced physically accurate PSFs by comparing its output with experimentally measured PSFs whose phase was altered by changing the length of one cavity arm (Supplementary Fig. 13). Numerical phase shifting of a measured 4Pi PSF is demonstrated graphically in Supplementary Video 1.

Using the phase-shifting algorithm, the time-dependent phase of the microscope's detection PSF may be measured directly from the localization data. Starting with a subset of molecule images corresponding to a short time window of the experiment, we fit the images with a series of phase-shifted PSF models. The mean value of Chi-square (〈*χ*^2^〉), averaged over the fit results, provides a measure of how well each model describes the data. After varying the model phase over 360°, we obtained plots of 〈*χ*^2^〉 versus phase for three different time windows (Fig. 2f). Each curve exhibits a single minimum, corresponding to the PSF phase at that time point.

The full temporal evolution of the PSF phase during an experiment can be determined using this approach. We found that as few as 250 emitter images were sufficient to measure the phase with a precision of 0.03 radians, allowing for a time resolution as high as 1 s for our data (Supplementary Fig. 14). The variation of the PSF phase during a full 4Pi-STORM image acquisition, with a duration of 800 s, is plotted in Fig. 2g. The phase undergoes short-timescale and long-timescale fluctuations, introducing a corresponding bias in the estimated *z* coordinates of the fluorophores.

Knowing the phase evolution, we calculated a phase-adjusted PSF model by shifting the phase of the initial model (derived from the bead scan) to the mean phase estimated for the experiment. The Nup96 dataset was reanalyzed using the new model, and phase drift was corrected by applying a time-dependent *z* shift to the molecule coordinates in proportion to the measured PSF phase evolution (Fig. 2g and Methods). This procedure resulted in a dramatic reduction in localization artifacts, as shown in Fig. 2d, while also improving image resolution by accounting for phase drift.

By including the time-dependent phase as an adjustable parameter of the spline representation, both the structure and dynamics of the 4Pi PSF can be modeled accurately. If left uncorrected, phase fluctuations introduce errors in the localization *z* coordinates, and larger phase shifts lead to phase-wrapping artifacts (ghost images). We refer to our model as a dynamic spline PSF, as it combines the strengths of a parametrized analytic description with a numerical model of the experimentally measured PSF structure.

### Localization precision and accuracy

**Fig. 3:**
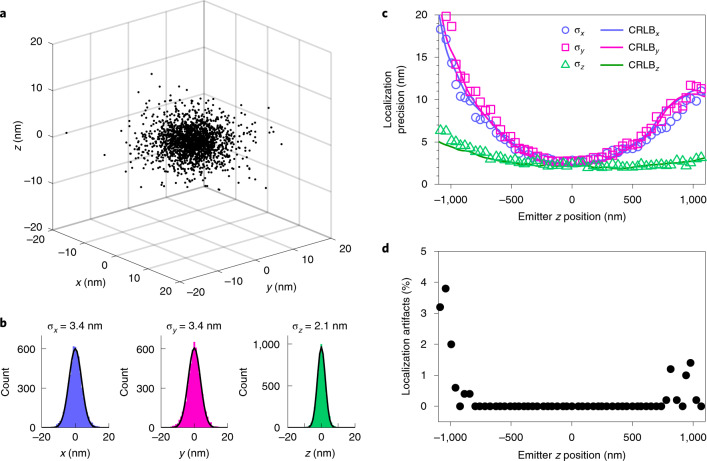
Localization precision and artifacts. **a**, Distribution of 3D position measurements for single Alexa Fluor 647 molecules, determined using the dynamic spline PSF model. The size of the distribution provides a direct measure of the localization precision of the microscope. On average, 8,002 photons were detected per fluorophore switching event. **b**, A 3D Gaussian fit to the histogram of the localization distribution yielded a standard deviation of 3.4 nm in the *x* and *y* dimensions, and 2.1 nm in the *z* dimension, corresponding to a spatial resolution with full width at half maximum values of 8 nm laterally and 5 nm axially. **c**, Variation of the localization precision as a function of the *z* coordinate of the emitter. By stepping a fluorescent bead through the focus of the microscope, the precision was measured over a 2.2-µm range. The bead brightness was similar to a single fluorophore (9,200 photons per frame). The CRLB, which estimates the theoretical limit of the precision on the basis of the information content of the data, is also shown (solid lines). **d**, Fraction of localizations erroneously assigned to the wrong interference fringe by the fitting procedure, as a function of the *z* coordinate of the emitter. When such an error occurs, the *z* position of the emitter is apparently shifted by a multiple of the PSF fringe spacing (*λ*/2), and this results in periodic ghost artifacts in the final image. Over a central range of 1.5 µm, no localization artifacts were observed.

We evaluated the performance of the microscope and the dynamic spline analysis by determining the localization precision and the frequency of localization artifacts. For this, we recorded 4Pi-STORM images of single molecules of Alexa Fluor 647, in which each fluorophore was localized multiple times. Localization clusters for 305 molecules with at least 10 localizations each were co-aligned, and the resulting distribution of position measurements (Fig. 3a) provided an estimate of the measurement precision^9,20,21^. The position distribution showed a 3D Gaussian profile with a standard deviation of 3.4 nm in the *x* and *y* dimensions, and 2.1 nm in the *z* dimension (Fig. 3b). The mean number of photons per switching event was 8,002 (Supplementary Fig. 16).

Next, we investigated the localization of emitters outside the focal plane. A fluorescent bead was scanned over a range of 2.2 µm, with the illumination adjusted such that ~9,200 photons were detected for each camera frame, similarly to a single fluorophore. By scanning the bead in steps of 20 nm (Supplementary Fig. 17), the precision could be measured at each position. We found that the dynamic spline fit reached the optimal precision in three dimensions, as measured by the Cramér–Rao lower bound (CRLB), over the full range of the scan (Fig. 3c), and exhibited no measurable bias (Supplementary Fig. 18 and Supplementary Notes 2 and 3).

The same measurement was used to determine the frequency of localization artifacts, which appeared at multiples of ~*λ*/2 above or below the true *z* position in the scan. Using the dynamic spline analysis, we found that such artifacts were almost entirely avoided. Over a range of 1.5 µm around the focus, we measured an artifact fraction of 0%, rising to ~5% for positions ±1.0 µm away from the focal plane (Fig. 3d).

**Extended Data Fig. 2:**
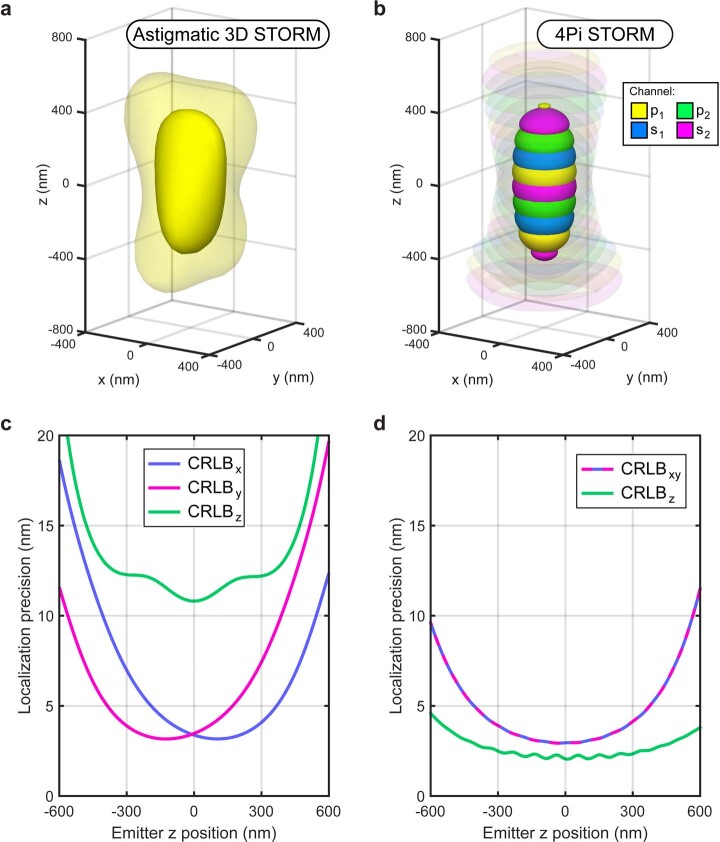
Comparison of standard astigmatic 3D STORM and 4Pi-STORM. **(a, b)** Side by side comparison of simulated PSFs for (a) single-objective 3D STORM based on astigmatism and (b) interferometric 4Pi-STORM. The astigmatic 3D STORM PSF was calculated for an oil immersion lens with a numerical aperture of 1.40 as described previously^21^, and the 4Pi-STORM PSF was calculated based on silicone immersion objective lenses with numerical aperture 1.35 as used in this study (Supplementary Note 4). Each PSF was rendered as isosurfaces at 35% and 8% of maximum intensity. **(c, d)** Comparison of the achievable localization precision for the two methods, as predicted by the Cramér-Rao lower bound on the localization precision in three dimensions. The CRLB was calculated based on the simulated PSFs, using realistic photon counts and background levels. For the astigmatic 3D STORM case, shown in (c), we assumed a mean photon count of 6000 photons per event^21^ and a background of 5 photons per pixel. For the 4Pi-STORM results, shown in (d), the mean photon count was 8000 photons per event and the background was 10 photons per pixel (Supplementary Table 1). The most significant difference is seen for the bound on the localization precision in the *z* dimension, which reaches a minimum of ~12 nm for astigmatic 3D STORM and ~2 nm for 4Pi-STORM near the focal plane (*z* = 0). Based on this estimate of the CRLB, we would expect a difference of a factor of approximately 6 in the achievable axial resolution between the two methods, consistent with previous results^10^. In practice, the performance of each method depends strongly on the microscope and the analysis algorithm used, as evidenced by the precision estimates obtained in earlier 3D-STORM experiments^21^.

The localization precision in the focal region (Fig. 3a,b) corresponds to an effective spatial resolution of 8 nm in the lateral (*x*–*y*) direction, and 5 nm along the optical axis (*z*). As a point of comparison, we note that the axial precision measured here is up to tenfold higher than that achieved with previous implementations of astigmatic 3D STORM^21^, highlighting the advantages of the 4Pi approach (Extended Data Fig. 2).

### Comparison with astigmatic 4Pi-SMLM

In previous implementations of 4Pi-SMLM, astigmatic imaging has been used as a means to avoid localization artifacts^13,16,22,23^. This method breaks the symmetry of the PSF along the *z* axis, and allows the interference fringe to be identified according to the ellipticity of each emitter image. To introduce astigmatism into the optical system, hyperbolic curved mirrors^16^ or electronic deformable mirrors^13,22,23^, are incorporated in the 4Pi cavity.

The astigmatic 4Pi approach has drawbacks, however. First, astigmatism causes the lateral extent of the PSF to increase dramatically for emitters positioned above or below the focal plane. This requires a larger area on the CCD detector for each emitter image, and can effectively limit the maximum density of fluorophores, reducing the achievable sampling resolution^24,25^. Second, the specialized mirrors required to shape the PSF add significant complexity and cost to the (already nontrivial) opto-mechanical design of a 4Pi microscope, and add a potential source of instability. Conversely, our measurements of the localization artifact frequency (Fig. 3d), obtained using an unmodified, symmetric 4Pi PSF, suggest that the dynamic spline approach renders astigmatism unnecessary.

**Fig. 4:**
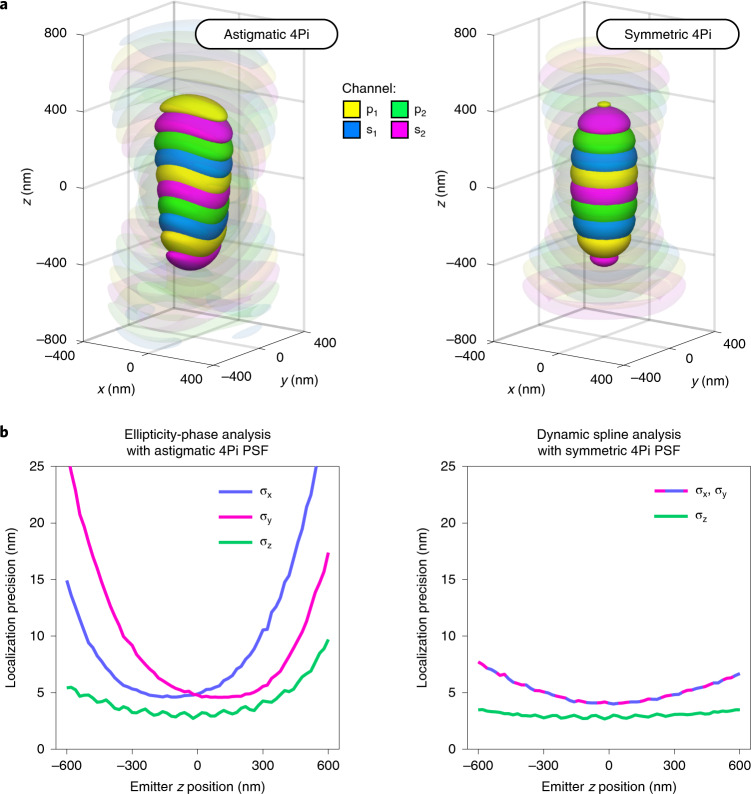
Comparison with astigmatic 4Pi PSF-based approaches. Previously reported 4Pi-SMLM microscopes use a shaped astigmatic 4Pi PSF, together with an analysis that measures both the ellipticities and the interference phases of the fluorophore images, to help avoid localization artifacts. **a**, Side-by-side comparison of simulated astigmatic 4Pi and symmetric 4Pi PSFs, rendered as four-color isosurfaces at 35% and 8% of maximum intensity. **b**, Localization precision comparison for the astigmatic 4Pi PSF using the ellipticity-phase analysis^13,22^ and the symmetric 4Pi PSF using the dynamic spline analysis. Simulated fluorophore images were generated on the basis of the two PSFs, using realistic photon counts (mean 8,000 photons) and signal-to-noise ratio (10 background photons per pixel), and the data were analyzed with the indicated method. The results show that while both approaches reached a similar localization precision at the focal plane (*z* = 0), for the astigmatic case the precision falls off sharply for positions further than 300 nm from focus. By contrast, the dynamic spline fit maintains a relatively constant precision over the full *z* range of the simulation. The frequency of localization artifacts for the two methods is evaluated in Supplementary Fig. 20.

To compare the methods, we created simulated data based on theoretical models of the symmetric 4Pi PSF and the astigmatic 4Pi PSF (Fig. 4a, Supplementary Fig. 19 and Supplementary Notes 4 and 5). For each case, we simulated single fluorescent emitters over a range of *z* positions, for typical experimental conditions. The data were analyzed with either the dynamic spline model (symmetric PSF) or the ellipticity-phase analysis^13,22^ (astigmatic PSF; Supplementary Note 6). The 3D localization precisions obtained for the two analyses are shown in Fig. 4b. Close to the focal plane, both methods yield similar lateral and axial localization precision (3–5 nm in *x*–*y*, and 2–3 nm in *z*). For |*z*| ≳ 300 nm, however, the astigmatic 4Pi PSF becomes strongly elliptical, spreading the detected photons over a larger area of the camera and causing the lateral localization precision (in the *x* or *y* dimension) to deteriorate sharply. In contrast, the symmetric 4Pi PSF, in combination with the dynamic spline analysis, yields a higher localization precision that is relatively insensitive to the emitter *z* position. This improvement is not only due to the PSF shape, but also because the dynamic spline PSF is a more accurate representation of the data. To support this view, we also created a cubic spline model of the astigmatic 4Pi PSF, and used it to fit simulated astigmatic 4Pi localization data. Modeling the astigmatic 4Pi PSF in this manner produced results more comparable to the symmetric 4Pi PSF (Supplementary Fig. 21).

Recent works have also developed new concepts for modeling the 4Pi PSF. For example, by including the phase as a free parameter of a 4Pi PSF model, a recent study examined the potential of estimating both the PSF phase and the *z* position simultaneously for individual fluorescent emitters in simulated datasets^26^. Also, phase retrieval was used to estimate the aberrations present in each arm of a 4Pi optical system^23^, allowing an analytic model of the astigmatic 4Pi PSF that is more accurate than the ellipticity-phase analysis, and obtains similar results to the spline fit in terms of localization precision. The dynamic spline method adopted here is distinguished, however, by its capacity to measure and compensate 4Pi PSF phase evolution with high temporal precision (Fig. 2g), in addition to being a simple and direct approach.

### 4Pi-STORM imaging of β-II spectrin

**Fig. 5:**
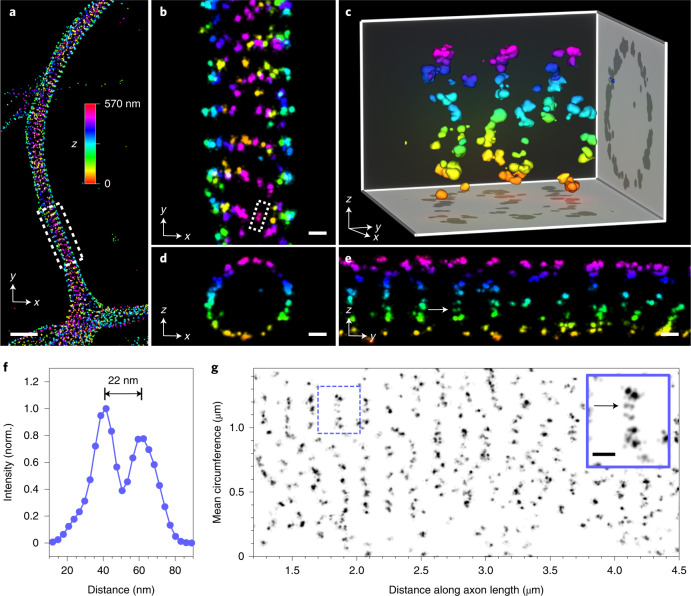
4Pi-STORM imaging of the neuronal cytoskeleton. β-II spectrin in the axon of a primary neuron, labeled with primary and secondary antibodies. Spectrin forms ring-like patterns around the circumference of the axon. **a**, Top view (*x*–*y*) of the axon, showing periodic spectrin stripes. Localizations are colored according to their *z* coordinate, as indicated by the color bar. Scale bar, 1 µm. **b**–**e**, Magnified views from the boxed region in **a**. Images show the neuron as viewed from the top (**b**), along its axis (**d**) and from the side (**e**). The near-isotropic resolution of the microscope allows these views to contain equivalent levels of detail. Seen from the side, within each stripe the spectrin signal appears to form doublets (white arrow in **e**), which also appear in the top view (**b**, white box). The image in **c** shows a 3D perspective view of a short section of the axon. Scale bars, 100 nm. **f**, A line profile through a spectrin doublet (white box in **b**) shows a spacing of 22 nm between the spectrin peaks. **g**, The spectrin signal as a function of its angular position around the axon circumference (*y* axis), plotted along the length of the axon (*x* axis). This view is equivalent to ‘unwrapping’ the spectrin into a 2D plane, and allows inspection of the spectrin organization with respect to the membrane. Spectrin doublets are visible in the plot (inset, black arrow). Some rings are curved as they wind around the axon, forming a spiral-like structure (for example, the region from 1.5 µm to 2.5 µm). The midpoint of the *y* axis corresponds to the top of the axon. The mean circumference of the axon in this region was approximately 1.4 µm. The inset (solid blue box) shows a magnified view of the region in the dashed blue box. Inset scale bar, 100 nm. Longer sections of unwrapped views from this sample are shown in Extended Data Figs. 5 and 6.

**Extended Data Fig. 3:**
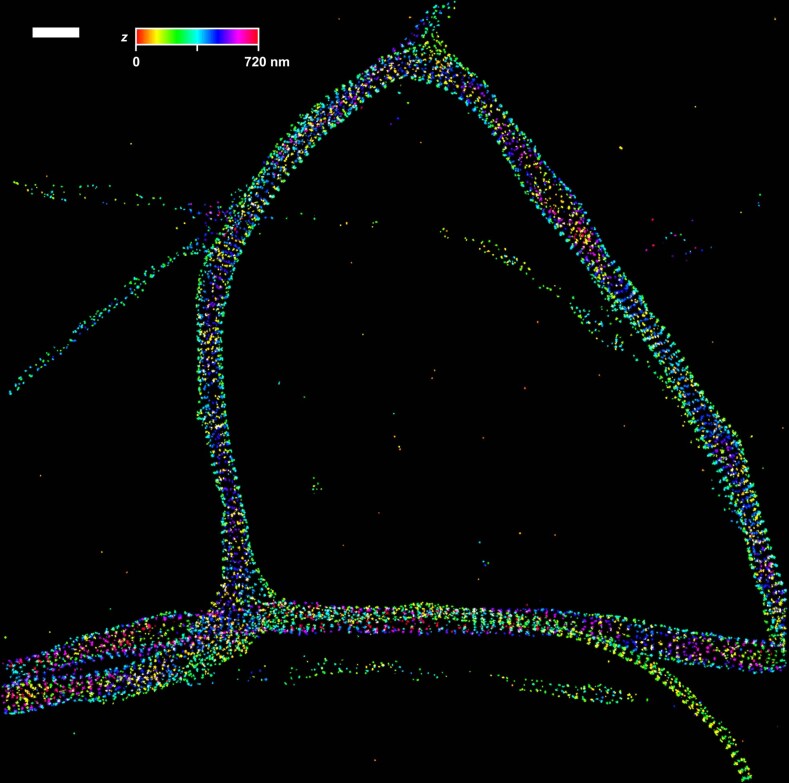
Beta-II spectrin in primary neuron cells. A larger view, showing the entire image, for the neuron sample displayed in Fig. 5. As described, the primary neuron cell is stained with antibodies against beta-II spectrin, and secondary F(ab)2 antibody fragments labeled with Alexa Fluor 647. The localizations were color-coded according to their *z* coordinate to show the depth information. Three-dimensional renderings of this dataset are shown in Supplementary Videos 2 and 3. Scale bar: 1 µm.

To characterize the capabilities of the microscope and the analysis pipeline in real applications, we imaged primary neuron cultures stained with antibodies against β-II spectrin, a component of the membrane-associated periodic skeleton (MPS)^27,28^. Viewed from above (*x*–*y* projection), the spectrin images show a characteristic pattern of stripes oriented orthogonally to the axis of the neuronal process (Fig. 5a,b and Extended Data Fig. 3). Closer inspection of the 3D data reveals spectrin molecules surrounding the axonal circumference, with few puncta inside the axon (Fig. 5b–e). At the surface of the axon, spectrin appeared in some regions as a linear series of double spots (Fig. 5e). A line profile through one such doublet is shown in Fig. 5f, exhibiting two peaks separated by 22 nm, which is compatible with the known structure of spectrin heterotetramers and the epitope recognized by the antibody^29^. Notably, each view (*x*–*y*, *x*–*z* and *y*–*z*) shows equivalent structural detail, due to the near-isotropic localization precision (Supplementary Videos 2 and 3).

**Extended Data Fig. 4:**
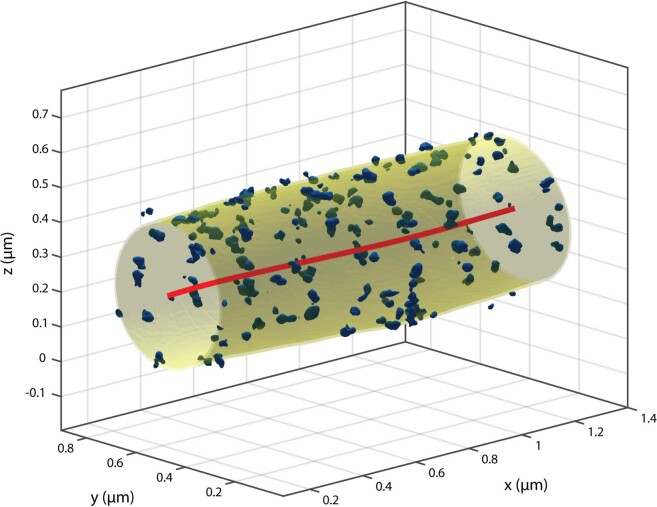
Mapping the surface of a neuronal process. Beta-II spectrin volume rendering corresponding to the data in Fig. 5b–e including the determination of the center line (red) and the tube-like surface (yellow) that best contains the beta-II spectrin localization data (see Supplementary Note 7). An animation illustrating the detection of the axonal membrane region, which is needed to generate the unwrapped view of beta-II spectrin, is shown in Supplementary Video 4.

**Extended Data Fig. 5:**
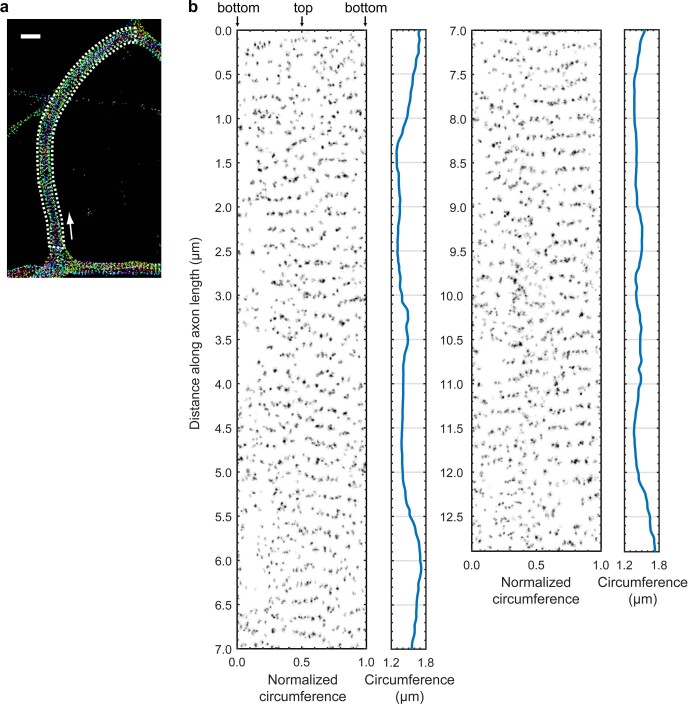
Unwrapped view of Beta-II spectrin (region 1). A longer section of the neuron shown in Fig. 5, digitally unwrapped to show the organization of beta-II spectrin in the cell. **(a)** Overview image, showing the region which was unwrapped (white box). The white arrow at one end of the box indicates the origin (coordinate 0) of the analysis and the direction of propagation. Scale bar: 1 µm. **(b)** The organization of beta-II spectrin relative to the plane of the neuronal membrane. The spectrin density is plotted as a function of the normalized distance around the circumference of the neuron (*x-*axis) and the distance along the length of the process (*y*-axis). The measured circumference of the neuron is plotted to the right of the spectrin density.

**Extended Data Fig. 6:**
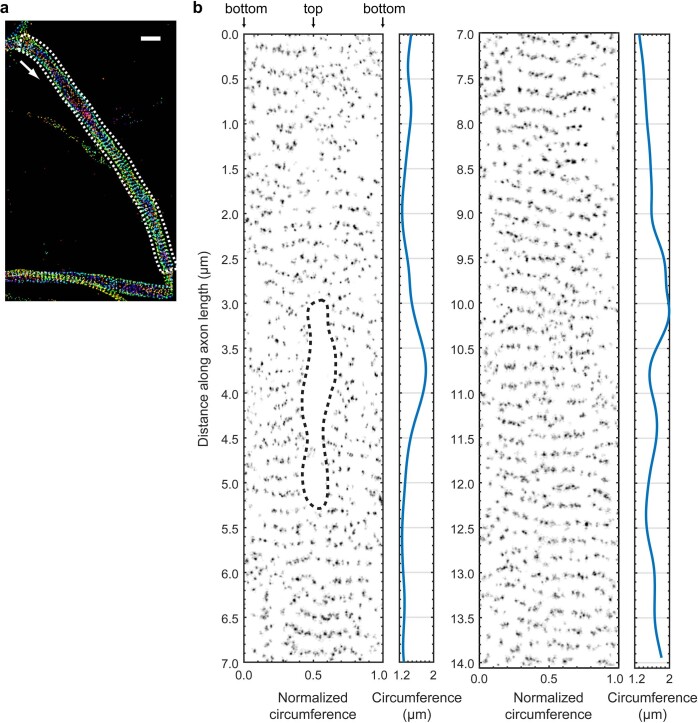
Unwrapped view of Beta-II spectrin (region 2). A longer section of the neuron shown in Fig. 5, digitally unwrapped to show the organization of beta-II spectrin in the cell. **(a)** Overview image, showing the region which was unwrapped (white box). The white arrow at one end of the box indicates the origin (coordinate 0) of the analysis and the direction of propagation. Scale bar: 1 µm. **(b)** The organization of beta-II spectrin relative to the plane of the neuronal membrane. The spectrin density is plotted as a function of the normalized distance around the circumference of the neuron (*x-*axis) and the distance along the length of the process (*y*-axis). The measured circumference of the neuron is plotted to the right of the spectrin density. Note that a gap in the spectrin density, marked by the dashed line, was due to the top of the neuron being out of focus in this region.

We analyzed the topology of the spectrin lattice by unwrapping the curving neuronal membrane onto a two-dimensional (2D) plane (Supplementary Note 7). A plot of the spectrin density around the circumference of the neuron, versus its position along the neuron’s length, yielded a 2D view of the membrane, which is easier to visualize (Fig. 5g, Extended Data Figs. 4–6 and Supplementary Video 4). This revealed details in the local organization of the MPS, such as gaps observed at the bottom of the axon, and Y-joints, kinks and bends, possibly indicating the presence of a splitting actin meshwork, or localized regulation of the tension and tilting of the MPS^30,31^. Intriguingly, within the space of few micrometers, the topology of the MPS can transition from an ordered series of perpendicular ring shapes, to a seemingly disordered pattern, to a tilted organization, which is consistent with a 3D spiral. A short section of this spiral-like structure is shown in the 3D perspective view (Fig. 5c).

The depth of field of the microscope, defined as the *z* range over which the resolution does not appreciably degrade, was ~1 µm (Fig. 3). Cellular samples are typically several micrometers thick, however, requiring a larger imaging depth. We addressed this issue by implementing stepwise *z-*stage scanning^13,32^. During the measurement, the sample was periodically shifted in *z*, with a step size of ~500 nm, over a range of 4–5 µm. Although this procedure reduces the numbers of localizations in each plane due to expenditure of switching cycles for undetected fluorophores, we found it to be an effective means of imaging moderately thick specimens. Supplementary Fig. 22 shows an example of a thick neuronal cell, imaged over ten *z-*planes for a total depth of ~5 µm.

### Multicolor imaging of Mic60 and mitochondrial nucleoids

**Fig. 6:**
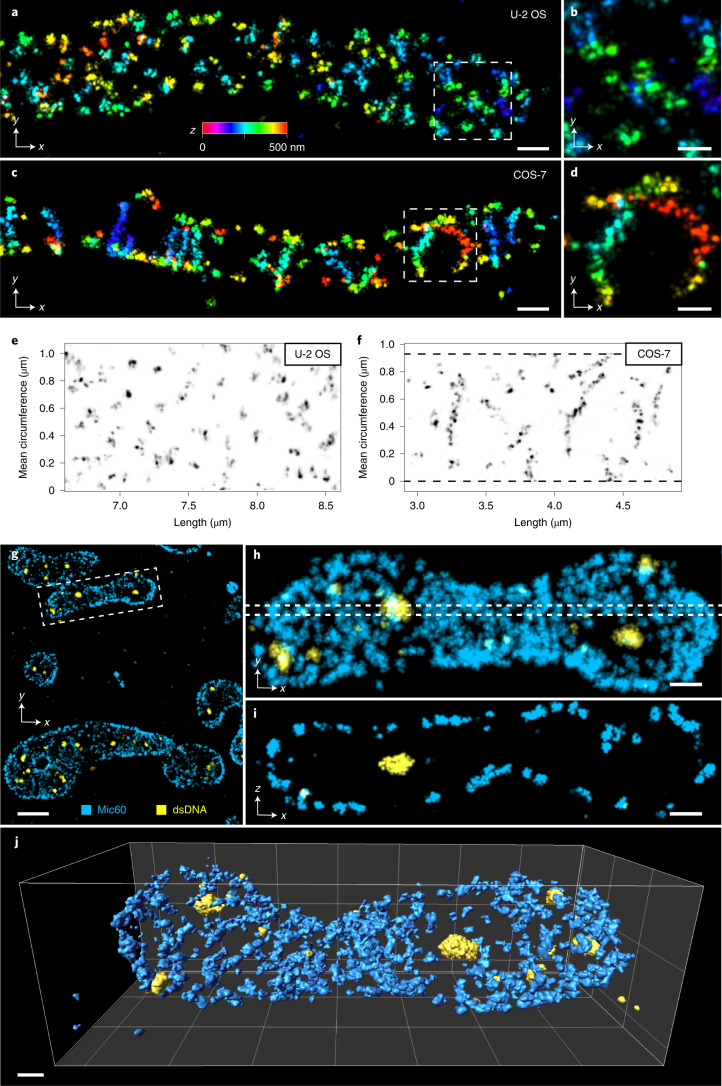
Single-color and multicolor imaging of Mic60 and mitochondrial DNA. Mic60 is a component of the MICOS complex, and is involved in the formation and maintenance of crista junctions that connect the crista membrane with the inner boundary membrane. **a**, Mic60 in a U-2 OS cell, labeled with primary and secondary antibodies. The Mic60 signals appear as structured, punctate clusters. The localizations are color coded according to their *z* coordinate (identical color scales in **a**–**d**). Scale bar, 200 nm. **b**, Magnified view of the boxed region in **a**. Scale bar, 50 nm. **c**, Mic60 in a COS-7 cell, in which the crista junctions exhibit a linear organization over segments of the inner boundary membrane. Scale bar, 200 nm. **d**, Magnified view of the boxed region in **c**. Scale bar, 50 nm. **e**,**f**, Unwrapped views of the Mic60 localization density around the surface of the mitochondria, showing the nanoscale distribution of Mic60. In U-2 OS cells, Mic60 appears predominantly punctate, with pairs or clusters of signal density separated by 20–40 nm (Extended Data Fig. 8 and Supplementary Fig. 23). In COS-7 cells, Mic60 appears to have a zigzag or double-line arrangement, with a typical width of approximately 25 nm (Extended Data Fig. 10 and Supplementary Fig. 24). Dashed lines indicate the extent of the data in **f**. **g**, Two-color image of Mic60 (blue) and mitochondrial nucleoids (yellow) in a COS-7 cell, stained with antibodies labeled with Alexa Fluor 647 and Cy5.5, respectively. Scale bar, 1 µm. **h**, Detailed view of the boxed region in **g**. Lower density of Mic60 close to the DNA signal, suggesting fewer crista junctions in these regions. **i**, Cross-section (*x*–*z*) through the region indicated by the dashed lines in **h**, showing Mic60 at the inner boundary membrane, and a DNA cluster in the center of the mitochondrion. **j**, A 3D perspective view of the mitochondrion shown in **h** and **i**, where the Mic60 and DNA signals have been rendered as isosurfaces. Scale bars, 250 nm (**h**–**j**).

The mitochondrial inner membrane forms numerous invaginations, with circular or slit-shaped openings, termed crista junctions. Crista junction formation requires the mitochondrial contact site and cristae organizing system (MICOS), a large multi-protein complex, which is embedded in the inner membrane. The nanoscale spatial organization of Mic60, the core component of MICOS, was recently investigated in a human bone osteosarcoma epithelial cell line (U-2 OS) using 3D MINFLUX nanoscopy^33^. Using 4Pi-STORM, we imaged Mic60 in U-2 OS cells and monkey kidney fibroblast-like cells (COS-7), to gain insight into the MICOS organization in different cell types (Fig. 6a–f).

In U-2 OS cells, Mic60 exhibited a 3D distribution which is mainly punctate (Fig. 6a,b). After determining the 3D envelope of the organelle, we unwrapped the data onto a 2D plane (as before for the MPS) making it easier to analyze its overall distribution (Fig. 6e). The data show that Mic60 puncta consist of 2–6 localization clusters spaced 20–30 nm apart, sometimes arranged as pairs in a short line, or sometimes forming circular groups of approximately 30 nm in diameter (Fig. 6b,e). These data further substantiate the view that in human U-2 OS cells, multi-protein arrangements of Mic60 surround, or line, individual circular crista junctions^33^.

**Extended Data Fig. 7:**
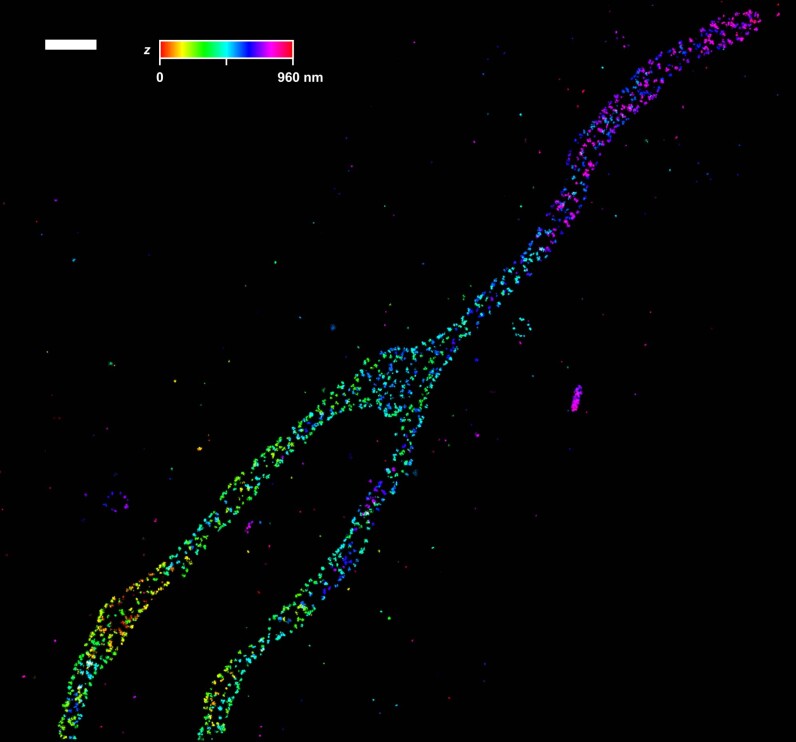
Mitochondrial Mic60 in U-2 OS cells. A larger view, showing the entire image, for the sample displayed in Fig. 6a. As described, the cell is stained with antibodies against Mic60, and secondary antibodies labeled with Alexa Fluor 647. The localizations were color-coded according to their *z* coordinate to show the depth information. A three-dimensional rendering of this dataset is shown in Supplementary Video 5. Scale bar: 1 µm.

**Extended Data Fig. 8:**
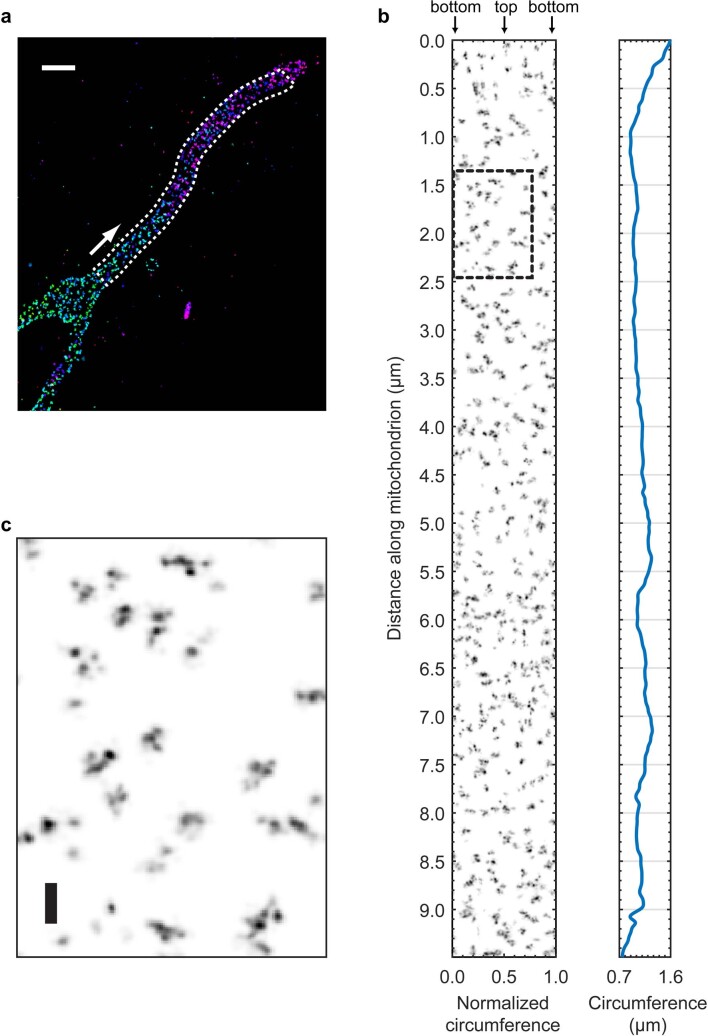
Unwrapped view of Mic60 in a U-2 OS cell. A longer section of the mitochrondrion shown in Fig. 6a, digitally unwrapped to show the organization of Mic60 at the inner boundary membrane. **(a)** Overview image, showing the region which was unwrapped (white box). The white arrow at one end of the box indicates the origin (coordinate 0) of the analysis and the direction of propagation. Scale bar: 1 µm. **(b)** The organization of Mic60 relative to the plane of the mitochondrial membrane. Mic60 density is plotted as a function of the normalized distance around the circumference of the membrane (*x-*axis) and the distance along the length of the mitochondrion (y-axis). The measured circumference is plotted to the right of the Mic60 density. **(c)** Zoomed view of the boxed region in (b). Scale bar: 100 nm.

**Extended Data Fig. 9:**
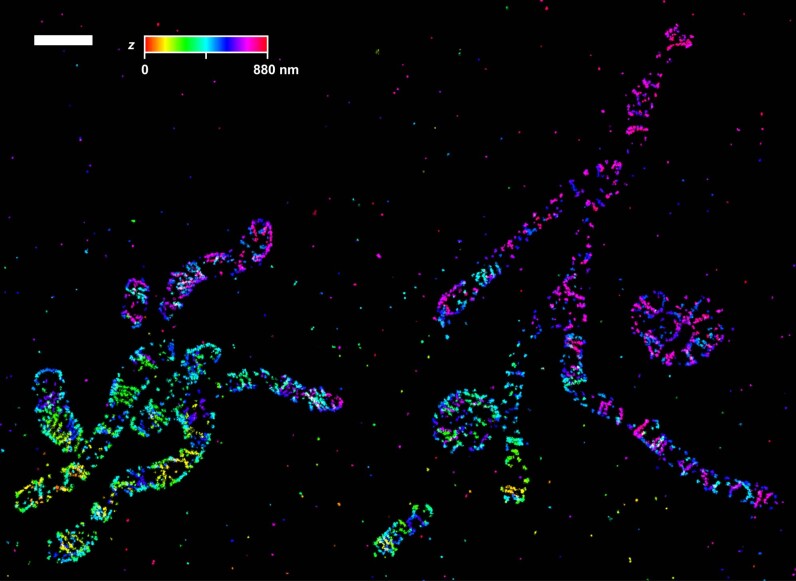
Mitochondrial Mic60 in COS7 cells. A larger view, showing the entire image, for the sample displayed in Fig. 6c. As described, the cell is stained with antibodies against Mic60, and secondary antibodies labeled with Alexa Fluor 647. The localizations were color-coded according to their *z* coordinate to show the depth information. A three-dimensional rendering of this dataset is shown in Supplementary Video 6. Scale bar: 1 µm.

**Extended Data Fig. 10:**
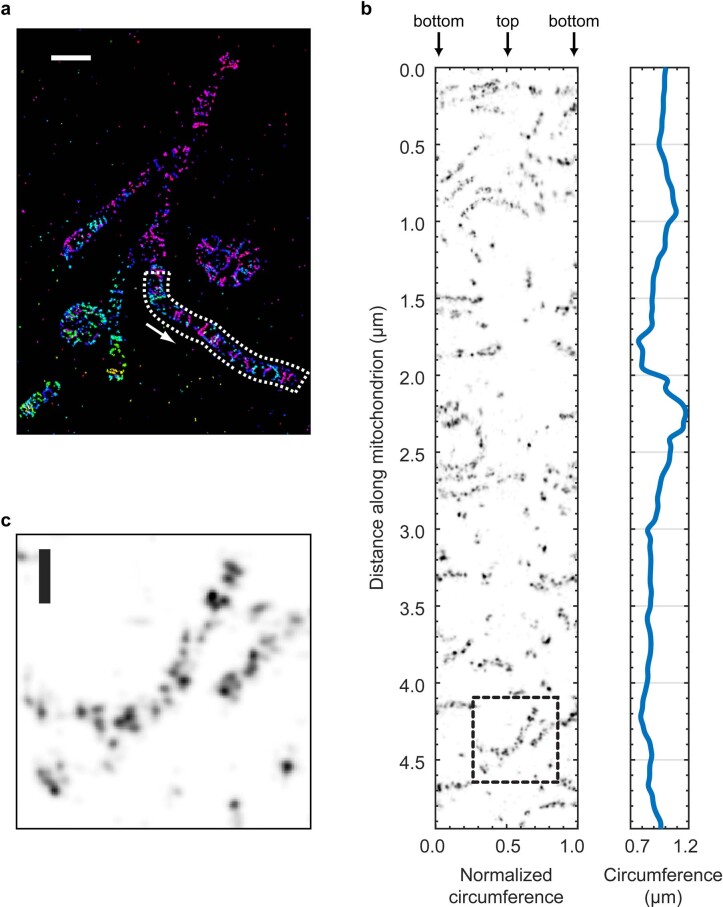
Unwrapped view of Mic60 in a COS-7 cell. A longer section of the mitochrondrion shown in Fig. 6c, digitally unwrapped to show the organization of Mic60 at the inner boundary membrane. **(a)** Overview image, showing the region which was unwrapped (white box). The white arrow at one end of the box indicates the origin (coordinate 0) of the analysis and the direction of propagation. Scale bar: 1 µm. **(b)** The organization of Mic60 relative to the plane of the mitochondrial membrane. Mic60 density is plotted as a function of the normalized distance around the circumference of the membrane (*x-*axis) and the distance along the length of the mitochondrion (y-axis). The measured circumference is plotted to the right of the Mic60 density. **(c)** Zoomed view of the boxed region in (b). Scale bar: 100 nm.

By contrast, in the mitochondria of COS-7 cells we observed that Mic60 often forms extended stripe-like arrangements (Fig. 6c,d). In these cells, Mic60 appeared in linear segments of varying length, and in some places these stripes appeared to comprise paired clusters of localizations, separated by ~25 nm (Fig. 6d,f). These data demonstrate differences in the sub-mitochondrial organization of MICOS in different cell types. Presumably, the differences in the Mic60 distribution have an immediate impact on the cristae structure in these cells. Further visualizations of Mic60 in the two cell types are shown in Extended Data Figs. 7–10, Supplementary Figs. 23 and 24, and Supplementary Videos 5 and 6.

The capability for multicolor, target-specific imaging allows visualization of the spatial organization of multiple cellular components. We implemented multicolor 4Pi-STORM using a ratiometric detection system^12,34^, and fit the localization data with fluorophore-specific dynamic spline PSF models. This allowed two photo-switchable fluorophores, Alexa Fluor 647 and Cy5.5, to be accurately localized with no significant loss of precision compared with single-color imaging, and efficiently discriminated with a color identification accuracy of approximately 93% (Methods and Supplementary Figs. 25–30).

We demonstrated multicolor imaging using COS-7 cells labeled for Mic60 and double-stranded DNA (dsDNA). Mitochondrial DNA is compacted into nucleoprotein complexes denoted nucleoids, which form ellipsoid-shaped clusters ~100 nm in size^35,36^. The dual-color images revealed mitochondria with peripheral, stripe-like distributions of Mic60, and dense clusters of DNA within the mitochondrial volume (Fig. 6g–j). Notably, the density of Mic60 labeling was reduced close to the nucleoids, indicating fewer crista junctions in those regions^37^. A detailed view of a single mitochondrion, together with a thin *x*–*z* cross-section (Fig. 6h,i), shows Mic60 localized to the inner boundary membrane, while nucleoids lie within the mitochondrial interior (Supplementary Video 7).

## Discussion

The strength of the 4Pi concept lies in the structure of the PSF. Self-interference of fluorescence photons, collected from both sides of the sample, introduces a sharp modulation along the optical axis, making the detected signal extremely sensitive to the 3D coordinate of the emitter^10^. Under conditions of spatially invariant illumination, the 4Pi PSF yields the highest localization precision per detected photon of any optical detection scheme^38^.

In this study, we developed an analysis framework that makes full use of the information content of 4Pi-STORM data by accurately modeling the PSF structure and dynamics, thereby obtaining optimal localization precision and accuracy. The dynamic spline PSF model allows the precise measurement and correction of phase transients, which is essential for accurate 3D fluorophore localization at single-nanometer length scales. For the conditions we tested, we found that the symmetric 4Pi PSF is ideal, achieving a higher localization precision and lower artifact rate compared with the astigmatic 4Pi PSF. Together, these developments enable robust and accurate 4Pi-STORM imaging, with a less complex instrument. Further, we anticipate that our approach may be generalized for use with other super-resolution microscopy techniques, for example, methods based on modulated excitation or detection PSFs^39,40^. The software tools developed for this work, including a GPU-accelerated multichannel spline fitting library, and algorithms for the creation of new spline models from 3D data, are published as open-source projects.

The 4Pi-STORM microscope enables the visualization of new structures in biological samples. Images of β-II spectrin in primary neurons (Fig. 5) showed the nanoscale organization of spectrin, and allowed the neuronal membrane to be mapped such that the topology of the spectrin rings could be digitally unwrapped and inspected. Images of crista junctions in mitochondria (Fig. 6) revealed distinct patterns of Mic60 in different cell types, and resolved the substructure of Mic60 stripes and puncta. We adapted our analysis for multicolor imaging, which is essential for studying the relative organization of multiple cellular components. The recently developed salvaged fluorescence concept^22^ would complement the methods described here by improving photon collection efficiency and multicolor discrimination. We note that the ‘salvaged’ signal could potentially be integrated as an additional channel of the PSF model, maximizing the information obtained.

## Methods

### Microscope design

The 4Pi-STORM microscope was designed and built using an optical layout based on earlier concepts^11,12^. The mechanical design includes new mounting and positioning systems for the objective lenses, mirrors, beam splitters and sample; a larger interferometric cavity; and the addition of a feedback control system for sample focus. The horizontal layout of the microscope also allows for easier construction and modification as compared with systems built on a vertically oriented optical breadboard.

### Objective lenses

The microscope uses two silicone oil immersion objective lenses (Olympus UPLSAPO100XS) with a focal length of 1.8 mm (magnification ×100), a numerical aperture (NA) of 1.35 and a working distance of 0.2 mm. One objective lens (obj. A) is fixed in position, mounted in a threaded cylinder, which is clamped into a V-block (Supplementary Fig. 5). The second objective lens (obj. B) is fully adjustable in terms of *x*, *y* and *z* position and tip/tilt. Obj. B is mounted inside the aperture of a three-axis piezo stage (Physik Instrumente, P-733.3DD; Supplementary Fig. 6). In this arrangement, the piezo stage allows fine control of the objective position in *x*, *y* and *z*. The entire assembly holding obj. B is mounted on sliding rails, which allow it to be retracted for sample insertion and extraction.

### Interferometric cavity and fluorescence detection

Fluorescence from the sample travels simultaneously along the upper and lower arms of the 4Pi interferometric cavity. In each of the two arms, the light passes through the objective lenses, dichroic mirrors (Chroma, ZT405/488/561/640/950RPC), quarter-wave plates (B. Halle, RAC3.4.15L) and modified Babinet–Soleil compensators (B. Halle) before reaching the 50:50 beam splitter (B. Halle TWK1.25; Extended Data Fig. 1). The dichroic mirrors allow the excitation light and control beams to be introduced into the system. The quarter-wave plates are set at an angle of 45° relative to the plane of the microscope, ensuring that fluorescence photons from the sample are equally distributed across all polarization angles. The compensator in the upper arm of the cavity (QC; Extended Data Fig. 1) consists of a fixed plate of BK7 glass and two adjustable wedges of quartz glass, such that the total thickness of quartz can be varied using a micrometer screw. This element allows a variable phase delay to be introduced between the s-polarized and p-polarized components of the fluorescence. In the lower arm, the compensator (GC) consists of a fixed plate of quartz glass and a variable thickness of BK7 glass, which can be adjusted to equalize the overall optical path length of the upper and lower arms and to correct for dispersion in the cavity. The compensators were adjusted such that the overall phase delay between the s-polarized and p-polarized fluorescence was 90°. The 50:50 beam splitter was mounted on a custom-made linear translation stage, incorporating a micrometer screw for coarse translation, adjustments for tip/tilt and a linear piezoelectric actuator (Physik Instrumente, P-841.1) for fine adjustment of the beam-splitter position.

The tube lenses (Linos, Achromat VIS, *f* = 500 mm; position L1 in Extended Data Fig. 1) form an image of the sample, which was masked by an adjustable rectangular aperture (OWIS SP60). After passing through the polarizing beam splitter (B. Halle, PTW 1.25), the fluorescence is split into four spatially separable components, consisting of two s-polarized and two p-polarized channels, each with a different interference phase. The telescopes formed by lenses L2 (Linos, Achromat VIS, *f* = 300 mm) and L3 (Linos, Achromat VIS, *f* = 250 mm) form four images of the sample on four quadrants of the EMCCD detector (Andor, Ixon Ultra 897). The effective magnification of the imaging system, including the objective lenses, tube lenses and telescopes, was 231.48×. Immediately before the camera, the fluorescence was filtered with a combination of spectral filters (Chroma, HQ700/75; Semrock, LP647RU and FF01-770SP). The four images formed on the four quadrants of the camera, numbered p_1_, s_1_, p_2_ and s_2_, correspond to a relative interference phase of 0, 90, 180 and 270°, respectively, such that a fluorophore that interferes constructively in channel p_1_ exhibits destructive interference in channel p_2_, and so on.

### Multicolor imaging configuration

When the microscope is configured for multicolor imaging, two additional filters are added to the detection path. The microscope was designed to discriminate fluorophores with strongly overlapping emission spectra by means of ratiometric spectral detection^12,34^. Two dichroic mirrors (Semrock, FF685-Di02), placed at an acute angle of 34° with respect to the fluorescence beam path, serve as polarization-selective long-pass spectral filters. The placement of the filters (DM3) is shown in Extended Data Fig. 1, and the filter spectra are shown in Supplementary Fig. 25. In this design, the ratio of s-polarized to p-polarized photons detected on the camera was ~0.6 for Alexa Fluor 647, and ~0.9 for Cy5.5, and this ratio may be used to distinguish the two dyes in the data analysis step. In the multicolor configuration, the bandpass filter (Chroma HQ700/75) was removed from the detection path.

### Sample mounting and positioning

Samples were prepared on 18-mm diameter glass coverslips (Marienfeld, 1.5H) with a thickness of 170 ± 5 μm. Before use, the 18-mm coverslips were sputter-coated with a thin aluminum layer on one side, covering one-quarter of the glass surface. This coating forms a mirror that was used for initial alignment of the sample and microscope objectives. For imaging, biological samples were prepared on the coated side of the coverslip. The 18-mm coverslip holding the sample was mounted onto a second, larger coverslip. For this purpose, 30-mm glass coverslips (Marienfeld, 1.5H) were glued onto a custom support consisting of a stainless-steel disc with an open aperture. Sample coverslips were then mounted in a sandwich configuration, by placing a droplet of imaging buffer onto the 30-mm coverslip, flipping the sample coverslip onto the droplet and removing excess buffer with vacuum suction. The coverslip sandwich was then sealed around the edge using fast-curing epoxy glue (Uhu). The assembly of the two coverslips, supported by the steel disc, was then magnetically coupled to a sample holder, which was connected to the sample translation stage. An isometric view of the stage assembly is shown in Supplementary Fig. 7.

The sample holder was mounted onto a three-axis translation stage (Newport, M-462-XYZ-M), which was coupled to two servo motors (Thorlabs, Z825B) for motorized sample positioning in *x* and *y*, and to a manual micrometer for initial focusing in *z* (parallel to the optical axis). Fine control of focus in *z* was provided by a linear piezo stage (Physik Instrumente, P-752) mounted underneath the top section of the translation stage.

### Excitation light path

A custom-built laser launch provided light for fluorophore excitation and activation. The following lasers were used for this purpose: red (642 nm, 1.5 W, MPB Communications, 2RU-VFL-P-1500-642), green (560 nm, 5 W, MPB Communications, 2RU-VFL-P-5000-560), blue (488 nm, 50 mW, Coherent Sapphire) and ultraviolet (405 nm, 100 mW, Coherent OBIS). The 642-nm, 560-nm and 488-nm beams were combined using dichroic mirrors and passed through an acousto-optical tunable filter (AA optoelectronic) for power control and modulation. Control of the 405-nm laser was done via software, and the 405-nm beam was combined with the other beams after the acousto-optical tunable filter. The four combined beams were coupled into a high-power polarization-maintaining single-mode fiber (OZ optics) via an achromatic fiber coupler (Schäfter & Kirchhoff, 60FC-4-RGBV11-47).

The optical layout of the illumination path is shown in Supplementary Fig. 1. Light exiting the fiber is collimated by lens L5 (Linos, Achromat VIS, *f* = 100 mm), passes through an adjustable aperture, and is focused by lens L4 (Linos, Achromat VIS, *f* = 500 mm) to the back focal plane of the fixed objective lens (obj. A), such that light exits the objective as a narrow collimated beam. By placing the output of the fiber in a plane that is conjugated to the back focal plane, and translating the fiber laterally with respect to this plane, the angle of the excitation beam at the sample could be adjusted between epifluorescence and total internal reflection fluorescence illumination modes. Typically, for 4Pi-STORM experiments, the illumination angle was adjusted to obtain the best signal-to-background ratio for a given sample.

### Objective alignment control system

Alignment of the two objective lenses must be maintained throughout the 4Pi-STORM experiment. To achieve this, a control system monitors the relative alignment of two lenses via an infrared laser beam, and continuously adjusts the three-axis piezo stage supporting obj. B, to maintain its lateral and axial position with respect to obj. A. The optical layout of the control system is shown in Supplementary Fig. 2. Laser light from a fiber-coupled laser diode (830 nm; Thorlabs, LP830-SF30) was collimated by lens L6 (Linos, *f* = 40 mm), and the size of the beam was adjusted by a variable aperture. The telescope formed by lenses L7 (Linos, *f* = 150 mm) and L4 expand the beam to a diameter of approximately 6 mm. The dichroic mirror DM2 (Chroma, HC670SP) was used to couple the beam into the excitation path. After obj. A, the beam is focused to a point inside the sample, and is then re-collimated by obj. B (Supplementary Fig. 2) when the two lenses are co-aligned. The outgoing beam exits the cavity at DM1 in the lower cavity arm and enters the beam deflection analyzer, which consists of a quadrant photodiode and two standard photodiodes located behind pinholes^12^.

### Sample focus control

The distance between the sample and the fixed objective lens is actively monitored during the experiment, to correct for sample drift along the *z* axis (the optical axis). For this purpose, a focus lock system was incorporated into the 4Pi-STORM microscope design. The system consists of an infrared laser (940 nm; Thorlabs, LP940-SF30), which is collimated and passes through a circular aperture, before being directed to one edge of the rear aperture of the fixed objective lens (Supplementary Fig. 3). A translation stage facilitates adjustment of the incoming beam position with respect to the objective. The beam emerges from the objective at an angle, focuses to the glass–water interface at the sample and is reflected back into the objective lens. The beam is re-collimated, and returns along the same path until reaching the 50:50 beam splitter, where part of the light is reflected and focused onto a CCD camera. Any change in the distance between the objective lens and the sample results in a lateral shift in the position of the returning beam, which is detected on the CCD. A software-based feedback system detects this shift and corrects for it by adjusting the position of the piezoelectric element built into the sample stage.

### Data acquisition and control systems

The microscope was controlled with custom software written in LabVIEW. Specifically, LabVIEW interfaces were written to control and monitor the EMCCD camera, excitation lasers, shutters, AOTF, piezoelectric translation stages, servo motors, focus lock CCD, photodiodes, and other electronic components. Higher-level interfaces and inter-process communication allowed the integration of multiple signals to implement feedback loops such as the focus lock and objective alignment control systems, and user interfaces allowing, for example, sample focus control and stage scanning. Analog voltage inputs and outputs, and digital input/output was managed with two DAQ cards (National Instruments, PCI-6229 and PCI-6731).

### Single-molecule sample

For measurements of single fluorophores (Fig. 3), short dsDNA oligomers, each labeled with a single Alexa Fluor 647 molecule, were bound to the surface of a glass coverslip as described previously^20,41^. Biotinylated and/or fluorophore-modified 30-base DNA oligonucleotides were purchased, purified using PAGE and labeled with a fluorophore, from Eurofins Genomics. Complementary strands of DNA were annealed to form biotinylated dsDNA constructs that contained a single Alexa Fluor 647 label. Annealing was carried out by mixing equimolar amounts of the two complementary strands in 10 mM Tris-Cl (pH 7.5), 10 mM NaCl, heating to 90 °C for 2 min, and then cooling the mixture to room temperature in a heat block over a period of 1 h.

Two DNA oligonucleotides, *SDA* and *SDB*, were used to form the dsDNA constructs. Note that [A647] indicates an end-positioned Alexa Fluor 647 modification, and [BioTEG] indicates a biotin modifier. The DNA sequences are listed below, from 5′ to 3′.

*SDA*: CTGCTTCGCGATGTACGTGCCGGATATACG[A647]

*SDB*: CGTATATCCGGCACGTACATCGCGAAGCAG[BioTEG-Q]

DNA constructs were immobilized via a streptavidin-biotin linkage to a biotinylated BSA-coated glass coverslip. Biotinylated BSA coverslips were prepared by incubating the coverslip with biotinylated BSA solution (Sigma, A8549, 1 mg ml^−1^) for 60 s. The slides were then rinsed with buffer (10 mM Tris, 10 mM NaCl, pH 7.5), incubated with streptavidin solution (Thermo Fisher, S888, 0.5 mg ml^−1^), and rinsed again with buffer, followed by incubation with the annealed DNA solution (50 pM) to immobilize the DNA to the slide at a low concentration.

### Tissue culture and immunofluorescence

Experiments were performed using either standard COS-7 cells or U-2 OS cells obtained from American Type Culture Collection (ATCC), or gene-edited U-2 OS cells expressing a SNAP-tagged version of the nucleoporin Nup107 (CLS Cell Lines Service, U-2OS-ZFN-SNAP-Nup107 clone 294)^18^ or Nup96 (CLS Cell Lines Service, U-2OS-CRISPR-NUP96-SNAP clone 33)^42^.

COS-7 cells were cultured in DMEM containing 4.5 g l^−1^ glucose and GlutaMAX additive (Thermo Fisher Scientific) supplemented with 100 U ml^−1^ penicillin and 100 μg ml^−1^ streptomycin (Merck Millipore), 1 mM sodium pyruvate (Sigma Aldrich) and 10% (vol/vol) FBS (Merck Millipore). U-2 OS cells (ATCC) were cultured in McCoy’s medium (Thermo Fisher Scientific). The growth medium was supplemented with 10% FBS, 1% penicillin–streptomycin and 1% sodium pyruvate.

For antibody and SNAP staining, cell samples were plated on glass coverslips (18 mm, 1.5H, Marienfeld) which were previously one-quarter coated with an aluminum mirror surface (see above). Cells were grown in culture medium overnight. Before fixation, samples were washed twice in warm PBS.

For labeling of mitochondria, COS-7 cells were chemically fixed by adding 2 ml of a pre-warmed solution of 8% formaldehyde in PBS (137 mM NaCl, 2.68 mM KCl and 10 mM Na_2_HPO_4_, pH 7.4, 37 °C) to 2 ml of the culture medium. After 5 min, we exchanged the solution for 4% formaldehyde in PBS and incubated for 5 min. U-2 OS cells were chemically fixed by replacing the culture medium with 2 ml pre-warmed 4% formaldehyde in PBS for 5 min. The cells were permeabilized by applying 0.5% (vol/vol) Triton X-100 in PBS for 5 min and subsequently blocked with 5% (wt/vol) BSA in 0.1 M PBS/glycine for 20 min. For antibody staining, we diluted primary antibodies against Mic60 (Proteintech, 10179-1-AP; dilution 1:100) or dsDNA (Abcam, ab27156, dilution 1:3,000) in 0.1 M PBS/glycine containing 5% BSA (wt/vol). The samples were incubated for 1 h at room temperature before washing six times with PBS to remove any unbound labels. Primary antibodies were detected using Fab fragments coupled to Alexa Fluor 647 (Thermo Fisher, A21246; dilution 1:500) or secondary antibodies coupled to Cy5.5 (Jackson ImmunoResearch, 715-005-150, custom labeled, dilution 1:200).

For NPC labeling, U-2 OS cells were chemically fixed by replacing the culture medium with 2 ml pre-warmed 2.4% formaldehyde for 15 min and briefly quenched with 50 mM NH_4_Cl. The cells were then washed with PBS and permeabilized with 0.2% Triton X-100 for 15 min. After permeabilization, SNAP-tag expressing cells were washed three times in PBS for 5 min, followed by blocking in Image-IT (Thermo Fisher, R37602). The cells were then incubated with the SNAP substrate (Alexa 647 – benzylguanine, 1 µM, NEB S9136S) in 0.5% BSA + 1 mM dithiothreitol at room temperature for 2 h. Cells were then washed three times in PBS for 5 min, and stored in PBS.

### Neuronal culture preparation and labeling

Cultures of dissociated rat hippocampal primary neurons were prepared from postnatal day 0 (P0)–P2 Wistar rats of either sex and cultured on glass coverslips coated with 100 µg ml^−1^ poly-ornithine (Merck KGaA) and 1 µg ml^−1^ laminin (BD Biosciences). Procedures were performed in accordance with the Animal Welfare Act of the Federal Republic of Germany (Tierschutzgesetz der Bundesrepublik Deutschland, TierSchG) and the Animal Welfare Laboratory Animal Regulations (Tierschutzversuchsverordnung). According to the TierSchG and the Tierschutzversuchsverordnung, no ethical approval from the ethics committee was required for the procedure of euthanizing rodents for subsequent extraction of tissues, as performed in this study. The procedure for euthanizing P0–P2 rats performed in this study was supervised by animal welfare officers of the Max Planck Institute for Medical Research (MPImF) and conducted and documented according to the guidelines of the TierSchG (permit number assigned by the MPImF: MPI/T-35/18).

Cells were grown in the presence of 1-β-d-Arabinofuranosyl-cytosin (Merck KGaA) at 37 °C and 5% CO_2_. Cultures were fixed at 19 d in vitro in 4% PFA in PBS, pH 7.4, for 15 min and quenched for 5 min in PBS supplemented with 100 mM glycine and 100 mM ammonium chloride. Cells were permeabilized for 5 min in 0.1% Triton X-100, blocked with 1% BSA for 1 h, and incubated with anti-β-II spectrin primary antibody (BD Biosciences, 612563; 1:200 dilution) overnight at 4 °C. After washing in PBS, samples were incubated with secondary antibody (anti-mouse Alexa Fluor 647 Fab Fragment; Thermo Fisher, A21237; 1:500 dilution) for 1.5 h at room temperature and further washed. Samples were post-fixed in 2% PFA for 10 min, quenched for 5 min, rinsed and mounted for imaging.

### Calibration and alignment markers

Before mounting the sample, fluorescent beads were added on the surface of the coverslip, which act as fiducial markers for registration of the 4Pi image channels, and for the purpose of measuring the microscope PSF before imaging. Far-red fluorescent beads with a diameter of 100 nm (Thermo Fisher, F-8798) were added to the sample in PBS at a high dilution (1:2 × 10^6^), such that the beads were sparsely distributed on the coverslip surface. Beads were bound to the sample by briefly rinsing with 10 mM Tris pH 8 + 50 mM MgCl_2_. The sample was then washed three times in PBS and stored in PBS.

### Imaging buffer

Before imaging, samples were mounted in STORM imaging buffer, consisting of Tris (50 mM; pH 8.0), NaCl (10 mM), glucose (10 % wt/vol), β-mercaptoethanol (143 mM; Sigma, M3148), and an enzymatic oxygen scavenger system (1% vol/vol). The enzymatic oxygen scavenging system was added to the buffer immediately before use, and the 100× stock solution was prepared by mixing pyranose oxidase powder^43^ (10 mg; Sigma, P4234) with catalase slurry (80 μl; Sigma, C100) in PBS (170 μl), and centrifuging the mixture at 13,000 r.p.m. for 1 min.

### 4Pi-STORM image data acquisition

After mounting the sample, recordings were made in order to measure the registration parameters for the four image channels, and also to measure the PSF of the optical system. First, an isolated fluorescent bead was found on the sample (bound to the glass surface), and images of the bead were recorded at multiple positions in the field of view. This set of images defines the transform that maps the channels onto each other, and is used for co-registration. Next, an isolated fluorescent bead was centered in the field of view and scanned through the common focal plane of the objectives while recording a sequence of images on the camera. The depth of the scan was 3 µm, recorded over 3,000 steps. The resulting bead image stack serves as an initial measurement of the PSF.

After locating a region of the sample to be imaged, the sample piezo stage was adjusted to bring the sample into focus and the focus lock control system was started. Next, the intensity of the red illumination was increased to 10–20 kW/cm^2^, causing the fluorophores to switch off and induce stochastic reactivation due to the red light. The angle of the illumination was adjusted to maximize the signal-to-background ratio of the image data. The EMCCD camera recording was started, with a typical recording duration of 80,000–100,000 frames at 100 Hz. The EMCCD was operated in 2 × 2 binning mode, making the effective pixel size 32 × 32 µm, corresponding to an effective imaging region of 138.2 × 138.2 nm per pixel. During the measurement, the 405-nm laser was switched on, and its intensity slowly increased, to maintain a constant rate of fluorophore switching events over time.

### Channel transform calculation

Images of fluorescent beads recorded at different positions in the field of view were used to determine a polynomial transformation, which relates coordinates in each of the image channels to every other channel. The 4Pi-STORM data consist of four image channels: two for s-polarized fluorescence and two for p-polarized fluorescence, numbered p_1_, s_1_, p_2_ and s_2_. A sequence of images of bright fluorescent beads were segmented and fit as described above to determine the bead coordinates in each channel, creating a list of equivalent coordinate pairs between the channels. The POLYWARP function (IDL) was used to calculate a second-order (quadratic) polynomial mapping between each of the channel pairs using the coordinate list.

### Image transformation

The raw STORM image stacks, and the PSF calibration stack, were transformed to map the four image channels to a common coordinate system. Making use of the polynomial mapping calculated in the previous step, we transformed the image data from each channel to the coordinate system of channel p_1_. This was done using the POLY_2D function (IDL), which uses cubic convolution for interpolation. After this step, image data from the four channels were precisely co-registered to within a root-mean-square deviation of 2 nm or less.

### Calculation of spline coefficients for the PSF model

The bead calibration image stack was converted to a set of cubic spline coefficients describing the PSF of the microscope. First, a 17 × 17-pixel region of the image stack, centered on the bead, was cropped out of the stack. These data were downsampled along the *z* axis by a factor of 10, using an averaging filter. Next, the stack was smoothed along the *z-*direction using a boxcar averaging filter with a spatial width of 50 nm. After this procedure, the size of the image stack containing the bead scan was 17 × 17 × 300 pixels, corresponding to a physical volume of 2.35 × 2.35 × 3.00 µm.

These data were converted into a set of cubic spline coefficients, using standard linear algebra and an algorithm adapted from Babcock et al.^17^. The cubic spline PSF model has four channels, which were generated by running the spline coefficients calculator four times, once for each channel of the bead image data. A 3D cubic spline function has 64 polynomial terms for each volume interval, and thus the total number of spline coefficients needed to describe the four-channel 17 × 17 × 300 voxel bead image stack was 4 × 64 × 16 × 16 × 299 ≈ 1.96 × 10^7^ coefficients. These values define an analytic function representing a model of the microscope PSF. A new PSF model was measured before each set of 4Pi-STORM measurements.

### Numerical method for 4Pi PSF phase shifting

For the purpose of determining the phase of the interference modulation of the PSF, with respect to the sample focus, a method is needed to shift the phase of the PSF spline by an arbitrary amount.

The dynamic spline model of the PSF is based on experimental data, and hence there is no phase parameter that can be simply adjusted. To dynamically shift the phase of the PSF model, we devised the following approach. First, the initial PSF model was rendered as a four-channel 3D image stack over the original range of the data, and with the original sampling frequency. These data were then divided into two components: a modulation component (*h*_mod_), corresponding to the interference of the fluorescence, and a slowly varying envelope function (*h*_env_). The envelope function, given by equation (3), was measured using a Fourier low-pass filter applied to each column of pixels in the PSF data stack, along the *z* direction. For our measurements, the cutoff frequency of the filter was set to remove frequency components with a period shorter than 500 nm, well above the oscillation period of the interference pattern (~*λ*/2, where *λ* is the fluorescence wavelength).

The modulation component, given by equation (4), was determined by subtracting the envelope function from the rendered image stack. To shift the phase, we used a Hilbert transform applied to the modulation stack, along the *z* direction, according to equation (5). Taking the real part of the transform results in a new image stack ($h_{{{{\mathrm{mod}}}}}^{90}$) in which all sinusoidal components of the signal are shifted by 90°. A linear combination of *h*_mod_ and $h_{{{{\mathrm{mod}}}}}^{90}$ allows an arbitrary phase shift to be introduced, and the phase-shifted PSF is obtained by adding back the envelope function, according to equation (6). The dynamic spline PSF model is then created by calculating the new set of spline coefficients corresponding to $h_{{{{\mathrm{4Pi}}}}}^{{{{\mathrm{dyn}}}}}\left( {x,y,z,c,\Delta \varphi } \right)$.

### Initial segmentation of 4Pi-STORM data

Initial 2D localization of fluorophore switching events was done by summing the four data channels (after transformation), resulting in an image stack which is effectively free of interference effects. This stack was segmented to identify initial peak candidates, and each peak was fit with a 2D Gaussian function to obtain an initial measure of its centroid, brightness and width. Fluorophore switching events that persisted over multiple camera exposures were identified and grouped.

### Single-molecule localization with the dynamic spline PSF model

Fluorophore switching events identified in the previous step were extracted from the image stack by cropping square 7 × 7-pixel regions containing the molecule images out of the transformed, four-channel raw data stack. Switching events persisting for multiple frames were summed along the time axis. Each image was fit with the PSF model, to find the optimal alignment in *x*, *y* and *z* between the molecule image and the model, thus determining the 3D coordinate of the emitter. For this purpose, a multichannel cubic spline model function was implemented for the Gpufit curve fitting library^19^ (Supplementary Note 8). This model function accepts a set of N-channel, 3D spline coefficients, and minimizes the sum of the squared deviation between the model and the data (Chi-square), allowing the estimation of the 3D spatial offset, baseline and amplitude of the spline function that best matches the emitter image. Using a PC with an Intel i7-5820K CPU and an Nvidia GTX 1080 GPU, the fits were executed at a typical rate of 80,000 per second.

Owing to the periodic nature of the PSF, multiple local minima exist in the Chi-square landscape when fitting the PSF model to the emitter image. To sample the full parameter space, the fitting algorithm was started with multiple initial *z* offsets, spaced evenly along the *z* axis, spanning the full range of the spline model. The spacing of the starting points was smaller than the periodicity of the PSF, ensuring that all of the local minima were sampled during fitting. The best fit between the spline and the emitter image was determined by selecting the fit result with the lowest Chi-square value.

Accurate fit results depend on using a PSF model that correctly describes the experimental data, particularly with respect to the phase of the PSF modulation. For this reason, the time-dependent PSF phase was measured for each experiment, as described below. The PSF spline was then shifted to the mean experimental phase, and used for fitting the images of all emitters in the dataset.

### PSF phase estimation

A set of emitter images acts as an effective reporter of the PSF phase at any time point during the experiment. To determine the phase, the initial PSF model derived from the bead scan was used to generate a series of 12 phase-shifted PSF models, equally spaced in phase between 0 and 2π. The set of emitter images were then fit with each model, and the mean Chi-square value (〈*χ*^2^〉) was computed for each case. A plot of 〈*χ*^2^〉 versus phase shift (Δ*ϕ*) exhibited a sinusoidal pattern, with a minimum at the phase corresponding to the experimental PSF phase. The position of the minimum was determined by fitting a sine function to the 〈*χ*^2^〉 versus Δ*ϕ* data (Fig. 2f).

The time evolution of the PSF phase was measured by repeating the phase determination for multiple time windows spanning the full dataset. The precision and time resolution of the result was dependent on the number of localizations per time window (Supplementary Fig. 14).

### Phase drift correction

To correct the effects of PSF phase drift, the proportionality factor between phase shift and apparent *z-*coordinate shift was determined for each measured 4Pi PSF in the following manner. Data slices taken from the PSF, corresponding to individual *z* planes, were fit with phase-shifted versions of the full PSF, with phase shifts spanning a range of 2 radians. For each phase shift, the fit returned a shifted *z* coordinate. A plot of phase shift versus *z* shift exhibits a linear relationship, and the slope of this curve yields the proportionality between *z* shift and PSF phase. After determining the time-dependent experimental PSF phase as described above, phase drift was rescaled to *z* drift, and subtracted from the *z* coordinates of the localizations.

We note that the lateral variation of the PSF phase across the field of view (Supplementary Fig. 15) is typically small enough (±40°) so as not to present a problem with respect to localization artifacts. As long as the PSF model used for fitting the localization data is within 60° of the true detection PSF phase, no increase in artifacts will occur (Supplementary Figs. 11 and 12).

For datasets with large phase drift (greater than 60°) during a single measurement, localization artifacts can be avoided by dividing the dataset into time windows, and analyzing each with a PSF model having a phase shift optimal for that window. This was not typically necessary for our data, with the exception of multistep scanning measurements (see below).

### Sample drift correction

Sample drift was measured by dividing the set of fluorophore localizations into time windows, rendering a 4Pi-STORM image for each time window, and calculating the 3D correlation function between images from different time points. The centroid of the peak of the correlation function was determined by fitting with a Gaussian function, and this coordinate represents the spatial offset between different time points, as described previously^20^. We used the robust cross correlation approach, measuring the correlation between all pairs of time windows, to obtain the optimal 3D drift trajectory, which was then subtracted from the localization coordinates^44^.

### Wavelength and refractive index corrections

Several factors necessitated corrections to the *z* coordinates. First, we accounted for the difference in the mean wavelengths of the fluorescence detected from the calibration bead (712 nm) and the fluorophores (Alexa Fluor 647, 680 nm; Cy5.5, 699 nm), which results in a small change in the period of the PSF modulation. The second correction was for the difference in the refractive indices of the sample (1.346) and the immersion oil (1.406). This is relevant due to the way in which the PSF was measured, by scanning the bead using the piezo stage rather than moving an emitter within the sample medium. Both corrections were implemented as a linear rescaling of the *z* coordinates obtained from the PSF model fit.

### Multistep scanning data analysis

Multistep stage scanning was used to record 4Pi-STORM images of thick regions of the sample (Supplementary Fig. 22). During the recording, the *z* position of the sample stage was periodically shifted up or down by uniform increments in a staircase pattern, covering the depth of the desired imaging region. The typical step size was ~500 nm, such that the 4Pi-STORM image data from neighboring steps would overlap partially, for later registration.

When the sample is shifted, the phase of the PSF also shifts, and this must be accounted for in data analysis. The approximate PSF phase shift Δ*φ*, for a shift in the sample position by Δ*z*, is given by equation (7):

7 $$\Delta \varphi = \left( {\frac{{n_{{{{\mathrm{imm}}}}}}}{{n_{{{{\mathrm{sample}}}}}}} - \frac{{n_{{{{\mathrm{sample}}}}}}}{{n_{{{{\mathrm{imm}}}}}}}} \right)\frac{{4\pi n_{{{{\mathrm{sample}}}}}}}{{\lambda _0}}\Delta z,$$

where *n*_imm_ and *n*_sample_ are the refractive indices of the immersion oil and the sample, respectively, and *λ*_0_ is the fluorescence wavelength in vacuum. This result is due to a sum of two effects, namely the shift of the focal plane when the sample is shifted, and the shift of the phase of the interferometric detection. We note that this result is only approximate, as it assumes objective lenses with low NA. In most 4Pi-SMLM microscopes with an index mismatch between the immersion oil and the sample, this is not the case.

We account for sample shifts by treating each step of the multistep scan as an independent measurement. For each step in the scan, the experimental PSF phase was determined separately during data analysis. The data for that step were then fit with a dynamic spline PSF model with the specific phase for that step, and phase correction was applied as described. In this manner, the PSF phase at each step was determined precisely without using equation (7). Small shifts in registration between 3D volume images from different scan steps were resolved during drift correction.

### Multicolor data analysis

Multicolor 4Pi-STORM data were recorded with additional filters in the detection path (DM3 in Extended Data Fig. 1), which act as polarization-sensitive spectral filters. For each localization event, the ratio of s-polarized to p-polarized photons was determined, and this ratio was used for initial assignment of the localization as one of the two fluorophore species (Alexa Fluor 647 or Cy5.5) according to a simple threshold (Supplementary Fig. 26).

The additional filter in the multicolor detection case causes each fluorophore to have a distinct detection PSF. In particular, with the filters in place, we found that the amplitudes of the s-polarized channels for the Alexa Fluor 647 PSF were attenuated by a factor of ~0.6 relative to the p-polarized channels. For Cy5.5, the s-polarized channels were attenuated by a factor of ~0.9. These values were measured directly from the data, by fitting a sample of localizations from each color species with a series of PSF models with varying s/p amplitude ratios (Supplementary Fig. 27).

After determining the correct PSF scaling for each fluorophore, final assignment of the fluorophore species for each localization was made by fitting with the PSF model. For this purpose, the localizations were fit with each fluorophore-specific (rescaled) PSF, and the color was identified by selecting the best-fitting result (lowest Chi-square value). This procedure minimizes cross-talk and ensures that the PSF model matches the true detection PSF for each localization, which is necessary to avoid introducing localization bias errors.

Using simulations, we investigated the effect of scaling the PSF model to have the correct amplitude ratio. Using a realistic PSF model (derived from a real PSF measurement), we generated simulated datasets for the two fluorophores, using an s/p amplitude ratio of 0.6 for Alexa Fluor 647 and 0.9 for Cy5.5. We then fit the data with either a correctly scaled PSF model or an incorrectly scaled model. Supplementary Fig. 28 shows that when the incorrect model was used to fit the data (for example, using a data ratio of 0.6 and a model ratio of 0.9), a *z*-bias error is introduced into the estimated localization coordinate. This bias is *z*-position dependent, meaning that it would cause distortions on the order of 5–10 nm, which vary over length scales of 100–200 nm, and cannot be simply corrected by an offset. Fitting the data with the correctly scaled PSF resulted in zero bias error, however.

We validated the simulation results by analyzing experimental data that were fit with a correctly scaled or an incorrectly scaled PSF model. A sample labeled with both fluorophores was analyzed by fitting all localization data with both versions of the PSF model. The fluorophore identity for each localization was determined using the s/p photon ratio, as above. For each set of fluorophore results, the difference in the estimated *z* coordinate between the fit with the correctly and incorrectly scaled PSF model is shown as a function of the *z* coordinate (assuming the correct result is obtained with the correctly scaled model; Supplementary Fig. 29). This experimental analysis reproduced a similar degree of bias error as seen in the simulations.

To explore the differences between the single-color and multicolor optical configurations, we simulated Alexa Fluor 647 and Cy5.5 molecules with photon counts and background levels obtained from the experimental data (Supplementary Table 1). The predicted localization precision versus the emitter *z* coordinate for each case is plotted in Supplementary Fig. 30. The results show that the localization precision for Alexa Fluor 647 is relatively unaffected by the configuration, with an average difference of 4% between the single-color and multicolor modes. In the multicolor mode, the *z*-localization precision for Alexa Fluor 647 is modulated as a function of the *z* coordinate (amplitude ≅ 1 nm) due to the uneven distribution of fluorescence between the s-polarized and p-polarized channels. We note that this modulation would be avoided when using a different multicolor detection method, such as salvaged fluorescence^22^. Finally, comparing the performance of the two fluorophores used for multicolor imaging, the simulation showed that, on average, Cy5.5 exhibited a 10% higher localization uncertainty than Alexa Fluor 647, due to its intrinsically lower brightness.

Despite the relatively small difference in the emission spectra of Alexa Fluor 647 and Cy5.5, some degree of chromatic aberration may still be present, which could introduce an overall *z* shift between the color channels. To correct for residual chromatic offset, a calibration sample was prepared in which the same structure (Nup107 in NPCs) was labeled with both fluorophores, allowing the effect to be measured from the data. The *z* offset between the fluorophores was determined to be approximately 10 nm, and subtracted from the data.

### Analysis software

A MATLAB script is provided with the paper to demonstrate a practical example of the 4Pi-STORM data analysis methods described here. The script encompasses the generation of the cubic spline PSF model, fitting the raw localization data, estimation of the PSF phase evolution and the dependence of the *z* coordinate on the phase, correction for PSF phase drift, sample drift and index mismatch, and visualization of the final localization coordinates. The script runs on a sample dataset, which is also provided. Further details of the supplementary data and software are given in Supplementary Note 9.

### Statistics and reproducibility

The data presented herein show representative results of our experiments. We have repeated all experiments three or more times, with multiple rounds of sample preparation. We obtained similar results for all measurements, with low statistical variation.

### Reporting Summary

Further information on research design is available in the Nature Research Reporting Summary linked to this article.

## Online content

Any methods, additional references, Nature Research reporting summaries, source data, extended data, supplementary information, acknowledgements, peer review information; details of author contributions and competing interests; and statements of data and code availability are available at 10.1038/s41592-022-01465-8.

## Supplementary information

Supplementary Information Supplementary Notes 1–10, Figs. 1–30, and Tables 1 and 2.

Reporting Summary

Supplementary Software Supplementary software and data package.

Supplementary Data Supplementary mechanical drawings.

Supplementary Video 1 The 4Pi PSF numerically phase-shifted over 360°. The four channels p_1_ (yellow), s_1_ (blue), p_2_ (green) and s_2_ (purple) of the 4Pi PSF were extracted from the scan of a fluorescent bead and rendered as a 3D volume. The phase of the PSF was numerically shifted through several cycles of 360°.

Supplementary Video 2 Neuronal cytoskeleton: detail of immunolabeled β-II spectrin in a primary neuron. Animation of a relatively linear section of an axon, taken from the dataset shown in Fig. 5. Localizations are colored according to their *z* coordinate.

Supplementary Video 3 Neuronal cytoskeleton: immunolabeled β-II spectrin in a primary neuron. Animation of the complete dataset shown in Fig. 5, including a fly-through of the axonal process. Localizations are colored according to their *z* coordinate.

Supplementary Video 4 Neuronal cytoskeleton: visualization of the creation of an unwrapped view. Sequential visualization of perpendicular (*x*–*z*) sections through the 4Pi-STORM image, sliding along the centerline of an axon in which β-II spectrin was immunolabeled (region shown in Extended Data Fig. 5a). To obtain an unwrapped view, an elliptical band was fit to the β-II spectrin distribution and integrated along the radial direction.

Supplementary Video 5 Crista junctions in mitochondria: immunolabeled Mic60 in a U-2 OS cell. Animation of the dataset shown in Extended Data Fig. 7. Localizations are colored according to their *z* coordinate.

Supplementary Video 6 Crista junctions in mitochondria: immunolabeled Mic60 in a COS-7 cell. Animation of the dataset shown in Extended Data Fig. 9. Localizations are colored according to their *z* coordinate.

Supplementary Video 7 Crista junctions and DNA nucleoids in mitochondria: immunolabeled Mic60 (blue) and dsDNA (yellow) in a COS-7 cell. Animation of the multicolor dataset shown in Fig. 6g–j, containing a sequential visualization of thin-slice sections through a single mitochondrion from the 3D dataset.

## Data Availability

A sample dataset demonstrating the analysis methods described above, and mechanical drawings (CAD files) specifying custom microscope components, are available as Supplementary Data (further details are given in Supplementary Notes 9 and 10). All data that support the findings of this study are available from the corresponding authors upon request.

## Code availability

A full working example source code written in MATLAB, demonstrating the methods described above, is available as Supplementary Software. Furthermore, the full source code for the spline calculation and fitting routines are provided, under an open-source license, at https://github.com/gpufit/Gpufit/ and https://github.com/gpufit/Gpuspline/.
