# Supplementary material for: Optimal precision and accuracy in 4Pi-STORM using dynamic spline PSF models
Source: Nat Methods. 2022 May 16;19(5):603–12. doi: 10.1038/s41592-022-01465-8 (PMC9119851; doi:10.1038/s41592-022-01465-8)
Supplement: Supplementary file 1 — Supplementary Notes 1–10, Figs. 1–30, and Tables 1 and 2. [file 41592_2022_1465_MOESM1_ESM.pdf]

nature portfolio

https://doi.org/10.1038/s41592-022-01465-8

Supplementary information

Optimal precision and accuracy in 4Pi-STORM using dynamic spline PSF models

In the format provided by the authors and unedited

Supplementary Information for

Optimal Precision and Accuracy in 4Pi-STORM using Dynamic Spline PSF Models

Mark Bates[1,2*], Jan Keller-Findeisen[1], Adrian Przybylski[1], Andreas Hüper[1], Till Stephan[1,3], Peter Ilgen[1,3], Angel R. Cereceda Delgado[1,4], Elisa D'Este[5], Alexander Egner[2], Stefan Jakobs[1,3], Steffen J. Sahl[1], and Stefan W. Hell[1,4,*]

[1]Department of NanoBiophotonics, Max Planck Institute for Biophysical Chemistry, 37077 Göttingen, Germany
[2]Department of Optical Nanoscopy, Institute for NanoPhotonics, 37077 Göttingen, Germany
[3]Clinic of Neurology, University Medical Center Göttingen, 37075 Göttingen, Germany
[4]Department of Optical Nanoscopy, Max Planck Institute for Medical Research, 69120 Heidelberg, Germany
[5]Optical Microscopy Facility, Max Planck Institute for Medical Research, 69120 Heidelberg, Germany

* To whom correspondence should be addressed.
mark.bates@mpibpc.mpg.de, stefan.hell@mpibpc.mpg.de

Table of contents

Supplementary Notes 3
1 Estimation of localization artifact rate in Nup96 measurements 3
2 Bead scan localization precision and artifact rate calculation 3
3 Cramér-Rao lower bound of the position estimation 4
4 Calculation of Point Spread Functions 4
5 Generation of simulated fluorescent emitter images 5
6 Astigmatism-based 4Pi-SMLM position estimation 5
7 Unwrapping of Beta-II Spectrin and Mic60 data 6
8 Gpufit and Gpuspline software libraries 7
9 Supplementary analysis software and data 8
10 Supplementary mechanical drawings 8
Supplementary Figures 9
Supplementary Fig. 1: 4Pi-STORM excitation path. 9
Supplementary Fig. 2: Objective alignment control system. 10
Supplementary Fig. 3: Sample focus control. 11
Supplementary Fig. 4: 4Pi-STORM optical system overview. 12
Supplementary Fig. 5: Fixed objective mount. 13
Supplementary Fig. 6: Movable objective mount. 14

Supplementary Fig. 7: Sample mounting stage. 15
Supplementary Fig. 8: 4Pi-STORM interferometric cavity overview. 16
Supplementary Fig. 9: Individual channels of the 4Pi PSF. 17
Supplementary Fig. 10: Comparison between PSF spline and measured bead scan. 18
Supplementary Fig. 11: Artifact frequency vs. PSF phase error (simulation). 19
Supplementary Fig. 12: Artifact frequency vs. PSF phase error (experiment). 20
Supplementary Fig. 13: Validation of 4Pi PSF phase shift algorithm. 21
Supplementary Fig. 14: Precision of phase estimation. 22
Supplementary Fig. 15: PSF phase variation across the field of view. 23
Supplementary Fig. 16: Number of photons detected per localization event. 24
Supplementary Fig. 17: Bead step scan. 25
Supplementary Fig. 18: *z*-coordinate localization accuracy. 26
Supplementary Fig. 19: Simulated symmetric and astigmatic 4Pi PSFs 27
Supplementary Fig. 20: Comparison with Astigmatic 4Pi (ellipticity-phase) analysis. 28
Supplementary Fig. 21: Spline analysis with Astigmatic or Symmetric 4Pi PSF. 29
Supplementary Fig. 22: Beta-II spectrin in a thick neuronal cell. 30
Supplementary Fig. 23: Measurements of Mic60 organization in a U-2 OS cell. 31
Supplementary Fig. 24: Measurements of Mic60 organization in a COS-7 cell. 32
Supplementary Fig. 25: Polarization-specific spectral filter for multicolor imaging 33
Supplementary Fig. 26: Color discrimination and crosstalk estimation. 34
Supplementary Fig. 27: Multicolor PSF S/P scale factor determination. 35
Supplementary Fig. 28: *z*-coordinate bias due to PSF model scaling (simulation). 36
Supplementary Fig. 29: *z*-coordinate bias due to PSF model scaling (experiment). 37
Supplementary Fig. 30: Single color vs. multicolor localization precision. 38
Supplementary Tables 39
Supplementary Table 1: 4Pi-STORM localization data statistics 39
Supplementary Table 2: 4Pi-STORM localization filter parameters 40
Supplementary References 41

Supplementary Notes

1 Estimation of localization artifact rate in Nup96 measurements

In the central 6x6 µm region of the measurement of a single cell nucleus (a section of which is shown in Fig. 2b) the nuclear envelope is slightly curved and can be accurately estimated as a surface from the Nup96 data alone. For this, a distance weighted average *z*-position of all localizations within the central region was computed. In a second iteration, localizations far away from this surface were disregarded. The Gaussian weighting factor acted as a smoothing parameter for the computed surface. The *z*-position of the nuclear envelope estimation was then subtracted from each localization's *z*-position, to place all the data at a common reference point in the *z*-direction. The reference point was further shifted so that the histogram part referring to artifact free localizations was located at z=0 on average.

Non-specific labelling was visible everywhere in the sample. To control for it, a mask in the *x*-*y* plane was created that contained all nuclear pores. The nuclear pore mask included ~20% of the area and ~95% of the available localizations. Histograms of all surface corrected *z*-positions within that mask and outside of the mask were calculated. The histogram from outside of the mask was rescaled to account for difference in area and subtracted from the histogram of *z*-positions within the mask. The resulting histograms are shown in Fig. 2c and Fig. 2d.

The localization artifact rate was computed by comparing the number of surface corrected localizations within a z=±0.15 µm range to that in a z = ±0.45 µm range with the localizations in the 0.15-0.45µm range being the localizations in the next 4Pi PSF fringe, i.e. the most likely mis-localizations. The surface and nuclear pore mask detection for the PSF phase with the lowest artifact rate was used for all tested PSF phases.

2 Bead scan localization precision and artifact rate calculation

The bead step scan (Supplementary Fig. 17) featured 250 frames recorded at every scan position. In each recorded frame, a region of 7x7 pixels including the bead image was cut out and fit with the PSF model as described above. In order to estimate the localization precision, the standard deviation of the *z*-positions estimated within a scan step were calculated from the squared differences in coordinates $\{z_i\}$ of consecutive estimations:

$$\sigma_z(\{z_i\}) = \sqrt{\frac{1}{2N}\sum\nolimits_{i=1}^{N}(z_{i+1} - z_i)^2}$$

A localization was classified as localization artifact if the estimated *z*-position was more than 70 nm away from the expected *z*-position. Sample drift during the scan was corrected by subtracting a low-pass filtered smooth curve from the estimated *z*-positions over the recording time (for display only).

3 Cramér-Rao lower bound of the position estimation

We followed a standard approach, for example as outlined in Balzarotti et al.[1] Because the fluorescence was detected by an EMCCD camera, we modeled the data as Poisson distributed with a variance that is approximately two times the expected number of photons, and a pixel readout noise term. The probability to obtain a measurement $d(x,y)$ is then given by

$$P(d(x,y)|f,v_0) = \frac{1}{\sqrt{2\pi(2f(x,y)+v_0)}} e^{-\left(d(x,y)-f(x,y)\right)^2/(2(2f+v_0))}$$

with the model $f(x,y)$ defined as in Eq. 1, and the readout noise v_0. The entries of the Fisher information matrix $I(\theta)$ for the fit parameters θ (amplitude, background, *xyz*-positions) were then computed as expectation values

$$I(\theta) = \left\{-E\left(\frac{\partial^2 l}{\partial\theta_i \partial\theta_j}\right)\right\} = \left\{\sum_{x,y} \frac{1}{2f(x,y)+v_0}\left(\frac{\partial f(x,y)}{\partial\theta_i}\right)\left(\frac{\partial f(x,y)}{\partial\theta_j}\right)\right\}$$

4 Calculation of Point Spread Functions

We employed vectorial diffraction theory to compute the propagation of the electric field of a dipole emitter through the optical system[2-5]. For simulations of 4Pi detection, we considered a dipole emitter located close to the common focus of two high NA objective lenses, whose collected signal is detected on a pixelated, planar detector. Aberrations arising due to mismatch between experimentally present and design values of refractive indices or thicknesses of the different layers (sample, glass, immersion oil) of the optical setup were modeled with a Gibson and Lanni phase term[3]. We do not assume rotational symmetry in the aperture, to allow for the introduction of an additional astigmatic phase distortion for the astigmatic PSF case. The foci of both objective lenses were overlaid in *z*, such that the points of maximal detection efficiency coincide. The thickness of the sample layer was assumed to be 20µm with the focus being positioned at 2µm inside the sample. The emitter position was then moved from 0 to 4µm inside the sample. We averaged over all emitter dipole positions by taking the mean intensity for an *x*, *y* and *z*-oriented emission dipole, effectively simulating a freely rotating fluorophore or a fluorescent bead.

The calculated symmetric 4Pi PSFs were compared to measurements of the experimental 4Pi PSF of the microscope setup, for each objective lens individually, as well as the interference of the signal from both lenses. The modulation frequency, and the lateral and axial widths of the PSF envelope, were found to match well with the simulation when the numerical aperture of both lenses was lowered slightly, from 1.35 to 1.30. The generally asymmetric shape of the 4Pi PSF with respect to the *z*-position that is visible in the experimental PSF was qualitatively reproduced in the simulation.

For the introduction of astigmatism an additional phase distortion proportional to $x_a^2 - y_a^2$ (aperture coordinates) was introduced after collection of the light by the objective lenses and before focusing by the tube lens. The strength of the astigmatism was adjusted such that the

distance along the *z* dimension between the plane of minimal PSF width in *x* and the plane of minimal PSF width in *y* matched a previously reported astigmatic 4Pi-PSF[6]. This resulted in the phase apodization term $e^{i\pi 0.31(x_a^2 - y_a^2)}$. The corresponding symmetric 4Pi PSF without astigmatism simply did not feature such a phase apodization term.

For calculation of the single objective lens astigmatic PSF (Extended data Fig. 2), the simulation was adjusted to set the lens' numerical aperture to 1.40, and the index of the immersion medium was changed to match standard immersion oil (1.51).

5 Generation of simulated fluorescent emitter images

Some parts of the study required the generation of simulated raw localization data (fluorescent emitter images) for emitters located at a certain z-position and with a certain brightness and background level (e.g. Fig. 4). For this purpose, a spline model of the desired PSF was first created (see Methods), and then the mean expected signal within a region of camera pixels was computed by interpolating the PSF model to have suitable center, amplitude, and offset parameter values onto a grid reflecting the camera pixel positions. From this model, noisy data was created by drawing values from a Normal distribution with the model value as mean, and with a variance two times the model value plus a camera readout noise term, independently for each pixel. The additional variance effectively simulates data from an EMCCD camera. The *x-y* center positions were chosen so that the molecule location was uniformly distributed within the area of the peak camera pixel.

6 Astigmatism-based 4Pi-SMLM position estimation

Astigmatism-based 4Pi-SMLM data analysis was reported previously[6, 7], and this method was reproduced in our study for comparison purposes. We refer to the method as the ellipticity-phase analysis, because it is based on a measurement of both the ellipticity and the interference phase for each fluorophore image. For the ellipticity-phase analysis, the transformed images of the emitter in each channel were summed, and an elliptical Gaussian function was fit to the summed image, resulting in *x-y* coordinates of the localized event, and fitted widths σ_x and σ_y. From these peak widths, a monotonic measure m was calculated as:

$$m = \frac{2\pi}{40}\left(\frac{\sigma_x^3}{\sigma_y} - \frac{\sigma_y^3}{\sigma_x}\right)$$

This measure is monotonic within ~1.2 µm for the simulated astigmatic 4Pi PSF used in our study. A phase for the central Gaussian moment (φ_0) was also computed, and the combination of m and φ_0 allowed the unambiguous determination of the emitter's *z*-position. Simplifying the analysis somewhat, a 4Pi PSF with the known correct PSF phase was used in

the analysis of simulated data and the z-coordinate of the emitter was determined by finding the z-value which minimizes:

$$L(z) = w_m\big(m_d - m_c(z)\big)^2 + w_\varphi\big(\varphi_d - \varphi_c(z)\big)^2$$

where m_d is the monotonic measures of the data, $m_c(z)$ are the monotonic measures of the astigmatic 4Pi PSF, φ_d is the zero order central moment of the data, $\varphi_c(z)$ are the zeroth order central moments of the PSF, and w_m, w_φ are empirically determined weighting factors ($w_\varphi = 10w_m$).

7 Unwrapping of Beta-II Spectrin and Mic60 data

To create unwrapped views of the beta-II spectrin distribution along an axon, and of Mic60 distributions in mitochondria, we modeled the distributions as tubular structures that could be locally represented by a rotated, elliptical shell.

To generate the initial model, an approximation of the centerline coordinates of the structure (i.e. the smooth line that passes along the center of an axon or a mitochondrion) were chosen by hand. The chosen centerline was smoothed and divided into sections of equal length (40 nm). We then iteratively refined our model of the centerline, together with the model of the elliptical shape of the membrane at each position along the centerline. A Cartesian coordinate system (reference system) was constructed from a vector tangential to the centerline and two perpendicular vectors in the transverse plane. The latter two vectors were chosen such that initially one pointed upwards, i.e. in the positive z-direction, and such that the change in the vectors between consecutive centerline points was as small as possible. This allowed the reference coordinate system to be translated smoothly along the centerline. At each centerline position, the volume-rendered 4Pi-STORM data was linearly interpolated over a rectangular grid in the associated coordinate system, and a slice of a certain thickness (100-200nm) along the centerline direction was summed. An elliptical shell of a certain thickness (typically 80-100nm thick) was then fit to the sum image in the reference system (by maximizing the normalized cross-correlation), and a shift in the ellipse center (in x, y) as well as the lengths of the major and minor axes and the orientation angle of the ellipse were obtained. This was done for each position along the centerline independently, and the new centers as well as major and minor axis lengths and ellipse orientations were smoothed along the centerline coordinate. This fitting step was repeated until the process converged, i.e. the centerline did not change further, and the fitted ellipse sizes and angles were stable (typically ~10 iterations were performed).

In a final step, unwrapped views were created by progressing along the centerline in small increments (4 nm pixel size) and calculating a grid over an elliptical shell with a certain thickness (80-100nm) in the transverse plane using the ellipse parameters determined in the previous step (see Supplementary Video 4). The grid had a radial and an azimuthal direction, in which the azimuthal positions were equally spaced around the circumference of the ellipse. Equal spacing of grid positions around the circumference was obtained using the incomplete

elliptic integral of the second kind. The data was linearly interpolated onto this grid and summed along the radial direction, resulting in a measure of the beta-II spectrin or Mic60 signal intensity as a function of position around the circumference of the axon or mitochondrion. The aspect ratio of the unwrapped views was chosen so that the mean circumference coordinate and the length along the centerline were scaled equally, resulting in a physically accurate representation which minimizes warping of the data.

8 Gpufit and Gpuspline software libraries

Two open source software libraries were written to calculate spline coefficients and interpolated spline values, and to perform nonlinear least-squares curve fitting with the spline model functions.

Gpuspline (www.github.com/gpufit/Gpuspline) was used to create a cubic spline representation of the 4Pi PSF from pixelated bead scan data. The API function *calculate_coefficients_3d* calculates a set of coefficients for a single channel 3D pixel stack representing the x,y,z intensity distribution of the PSF in one channel. We called this function four times, once for each image channel, to obtain the spline coefficients describing the four-channel 4Pi PSF. The order of the spline coefficients representing a dataset and the corresponding Matlab or Python binding calls are described in the Gpuspline documentation (gpuspline.readthedocs.io/en/latest/index.html). Examples of the calculation of spline coefficients from a dataset, as well as interpolation of a dataset using the calculated spline coefficients, are included with the project source code.

Gpufit (www.github.com/gpufit/Gpufit) was used to fit the experimental data, using the cubic spline models generated with Gpuspline. For this purpose, we created new fit model functions for general purpose multichannel, multidimensional spline fitting. The arrays of spline coefficients for each image channel were concatenated, and served as the definition of the multichannel spline models in Gpufit. The fit model function *SPLINE_3D_MULTICHANNEL* was used for 4Pi STORM data analysis, and the concatenated spline coefficients were passed in using the *user_info* parameter of the *gpufit* API function call. In addition to fitting with multiple image channels, the dynamic phase of the 4Pi PSF (from Eq. 6 in the main text) was implemented as a model parameter in another Gpufit model function *SPLINE_3D_PHASE_MULTICHANNEL,* which requires three sets of multichannel spline coefficients passed via the *user_info* parameter, corresponding to the PSF envelope h_{env}, the modulation h_{mod}, and the shifted modulation h_{mod}^{90} as defined in Eqs. 3-5. The definitions of the spline fit model functions, the structure of additional user information for the fit models and the corresponding Matlab or Python binding calls are described in the Gpufit documentation (gpufit.readthedocs.io/en/latest/index.html). Examples for the use of a spline representation of a model function to fit data with Gpufit are included in the project source code (www.github.com/gpufit/Gpufit/tree/master/examples/matlab).

9 Supplementary analysis software and data

An additional software package is provided with the manuscript in order to demonstrate 4Pi-STORM data analysis using the dynamic spline PSF model. This package is written in Matlab, and contains experimental data including a fluorescent bead scan (experimental PSF), and the emitter images for the Nup96 dataset shown in Fig. 2. The package executes each step in the analysis, including the creation of the spline representation of the PSF (using Gpuspline), estimation of the phase evolution of the PSF and fitting the localization events with the optimal PSF phase (using Gpufit), correction for phase drift, and correction for sample drift.

The supplementary software and data are included with the supplementary materials of the manuscript, as a zip file: *supplementary_data_and_software.zip*. Usage instructions are contained in the file *README.txt*. This package includes Windows binary files for the Gpufit and Gpuspline libraries. Note that a CUDA-supported graphics processing unit (GPU) is required to run the software, as the fits are executed in parallel on the GPU.

10 Supplementary mechanical drawings

Digital mechanical designs are provided as supplementary data with the manuscript. Full 3D mechanical drawings for the fixed objective mount, movable objective mount, and sample stage (Supplementary Figs. 5-7) are saved in STEP format, a widely used format which is accessible from many CAD software environments. The drawings are contained in the zip file: *supplementary_mechanical_drawings.zip*. All mechanical designs are released under the terms of the CERN Open Hardware License Version 2 (Strongly Reciprocal).

Supplementary Figures

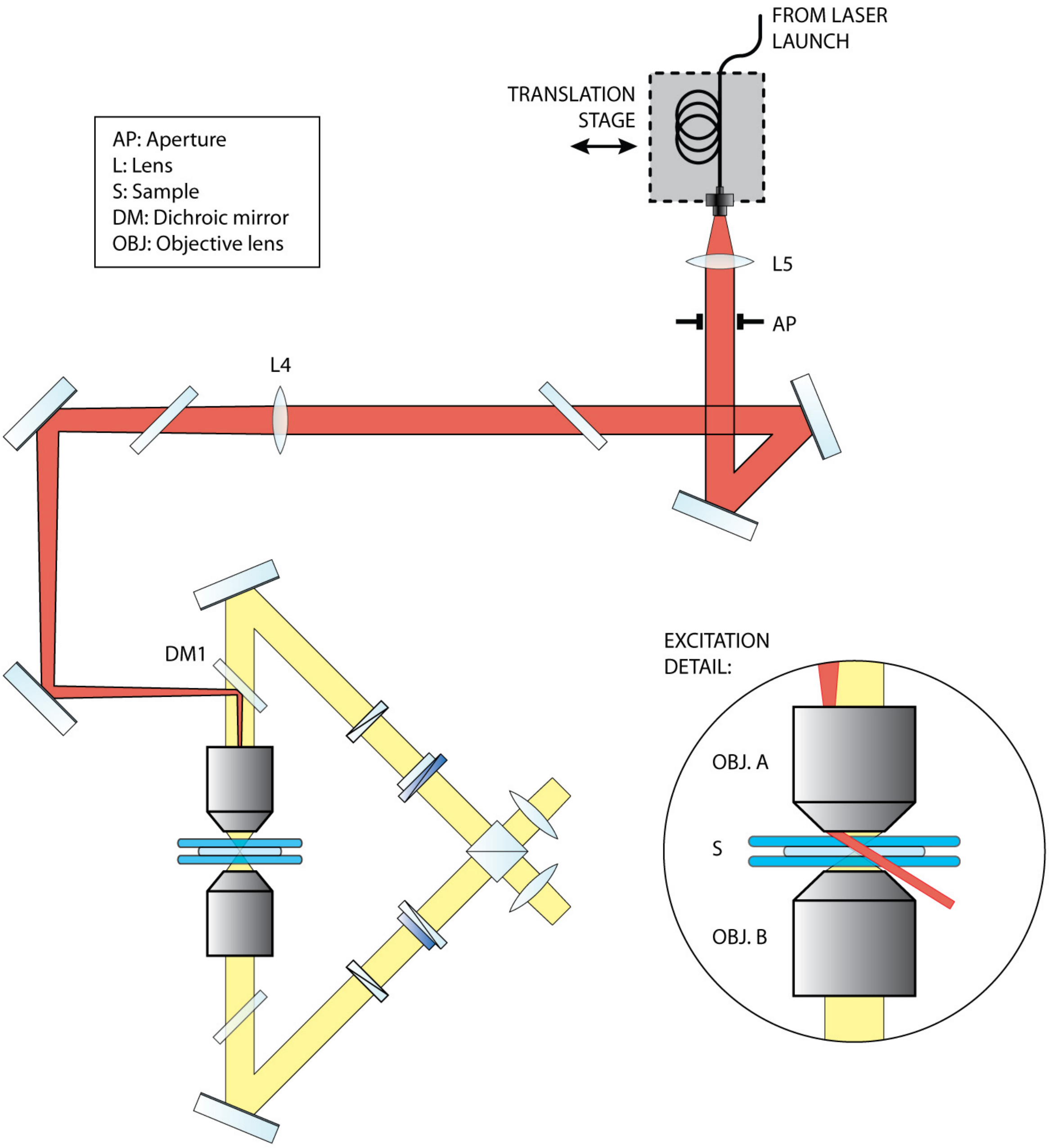

Supplementary Fig. 1: 4Pi-STORM excitation path. Optical layout for the sample illumination path. Laser light is coupled into a high-power, polarization-maintaining single mode optical fiber, and the output of the fiber is mounted on a translation stage. Light from the fiber is collimated by lens L5. Lens L4 focuses the beam onto the back focal plane of the objective lens (OBJ A). The excitation beam is coupled into the 4Pi cavity at the dichroic mirror DM1. The adjustable aperture (AP) is located in a plane which is conjugate to the sample plane, and may be adjusted to limit the extent of the illumination area. The output of the fiber is located in a plane which is conjugate to the back focal plane of the objective lens, and by translating the fiber output laterally, the angle of the excitation beam at the sample may be adjusted between epi-illumination and near-TIRF illumination modes (see excitation detail, inset).

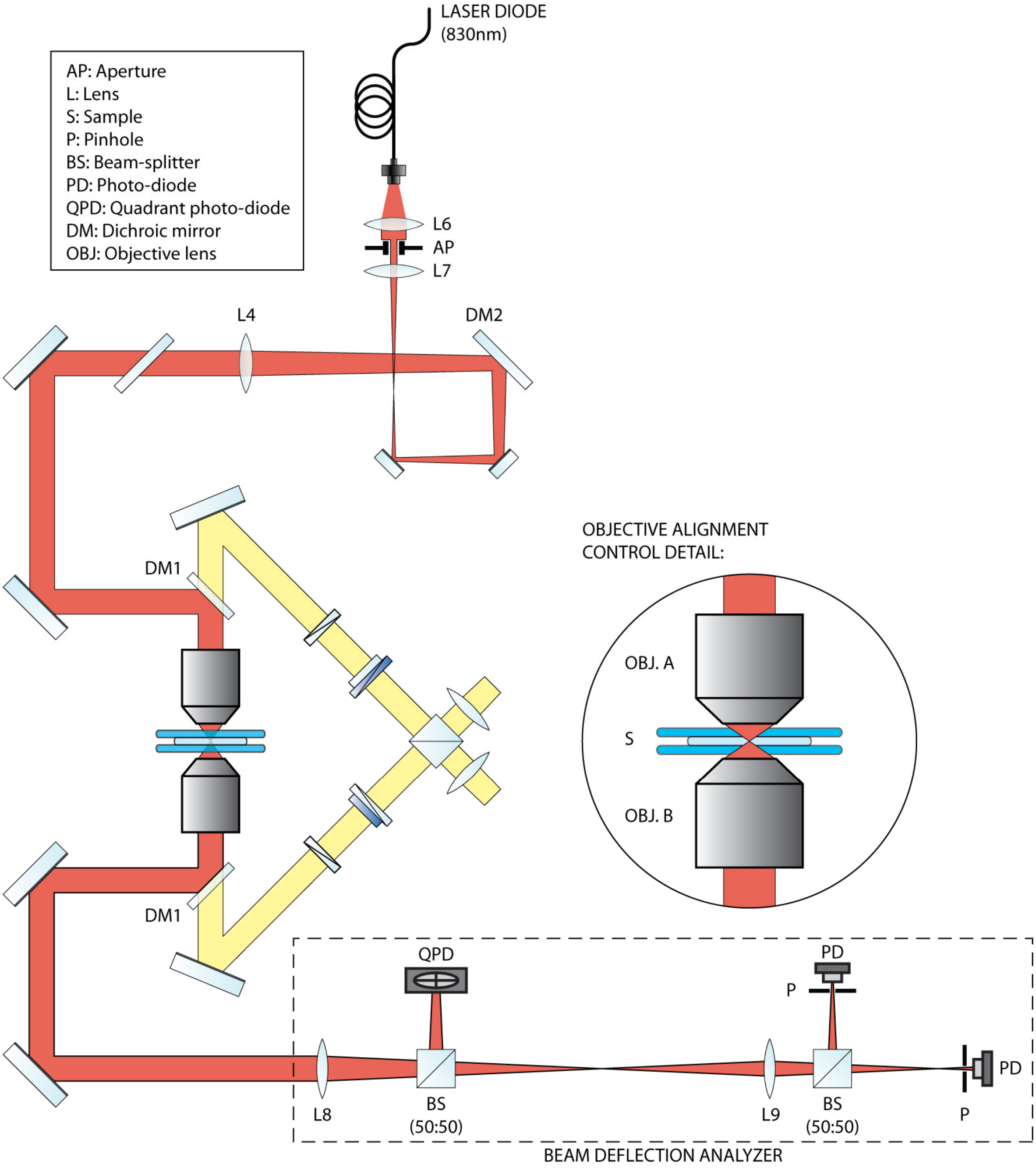

Supplementary Fig. 2: Objective alignment control system. A fiber-pigtailed laser diode at 830nm is collimated by lens L6, and expanded by lenses L7 and L4. The beam fills the back aperture of Objective A. The beam is focused to a point in the sample, and then re-collimated by Objective B (see detail, inset). Any lateral shift between the two objectives is detected as a shift in the angle of the outgoing beam. Any shift in the focus position of the two objectives is detected as a change in the collimation of the outgoing beam. The angle and collimation of the beam, after Objective B, are monitored using the deflection analyzer, consisting of a quadrant photodiode, and two additional photodiodes placed behind pinholes.

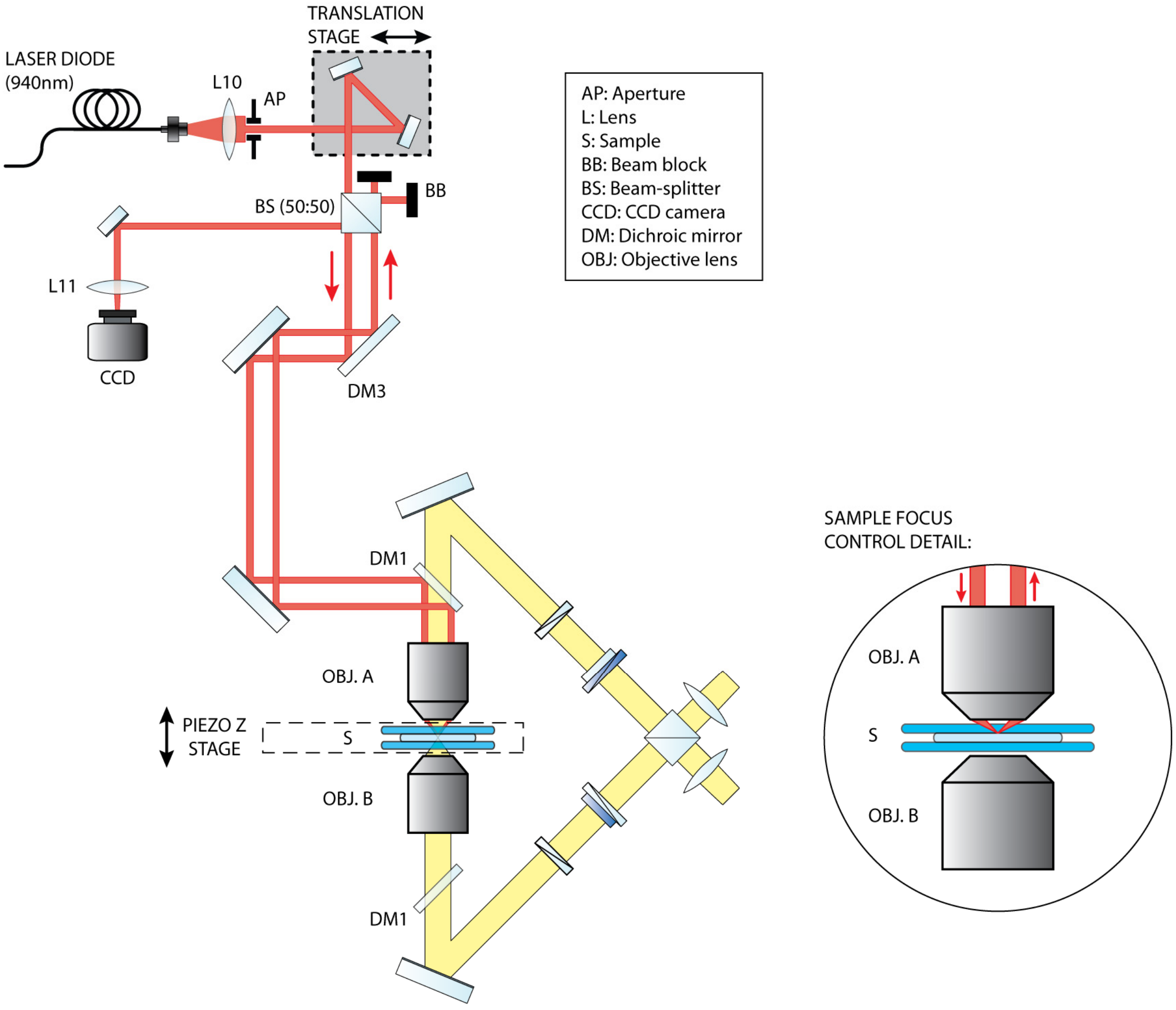

Supplementary Fig. 3: Sample focus control. The position of the sample with respect to Objective lens A is maintained by a feedback system, which is based on an infrared laser beam which reflects from the coverglass-sample interface and is detected on a CCD camera. Light from a fiber-pigtailed laser diode (940nm) is collimated, and directed to the edge of the back aperture of Objective A. A translation stage facilitates adjustment of the beam entrance position. Light emerges from the objective lens at a sharp angle (see inset), is reflected at the glass-water interface in the sample, and is re-collimated by the objective lens, exiting on the other side of the back aperture. The returning light beam is reflected at the beam splitter and focused onto a CCD camera. Any shift in the sample position along the optical axis, relative to the objective lens, is detected as a shift in the beam position on the CCD. By monitoring the beam position, sample drift is continuously corrected by adjusting the piezoelectric transducer (focus piezo) built into the sample translation stage.

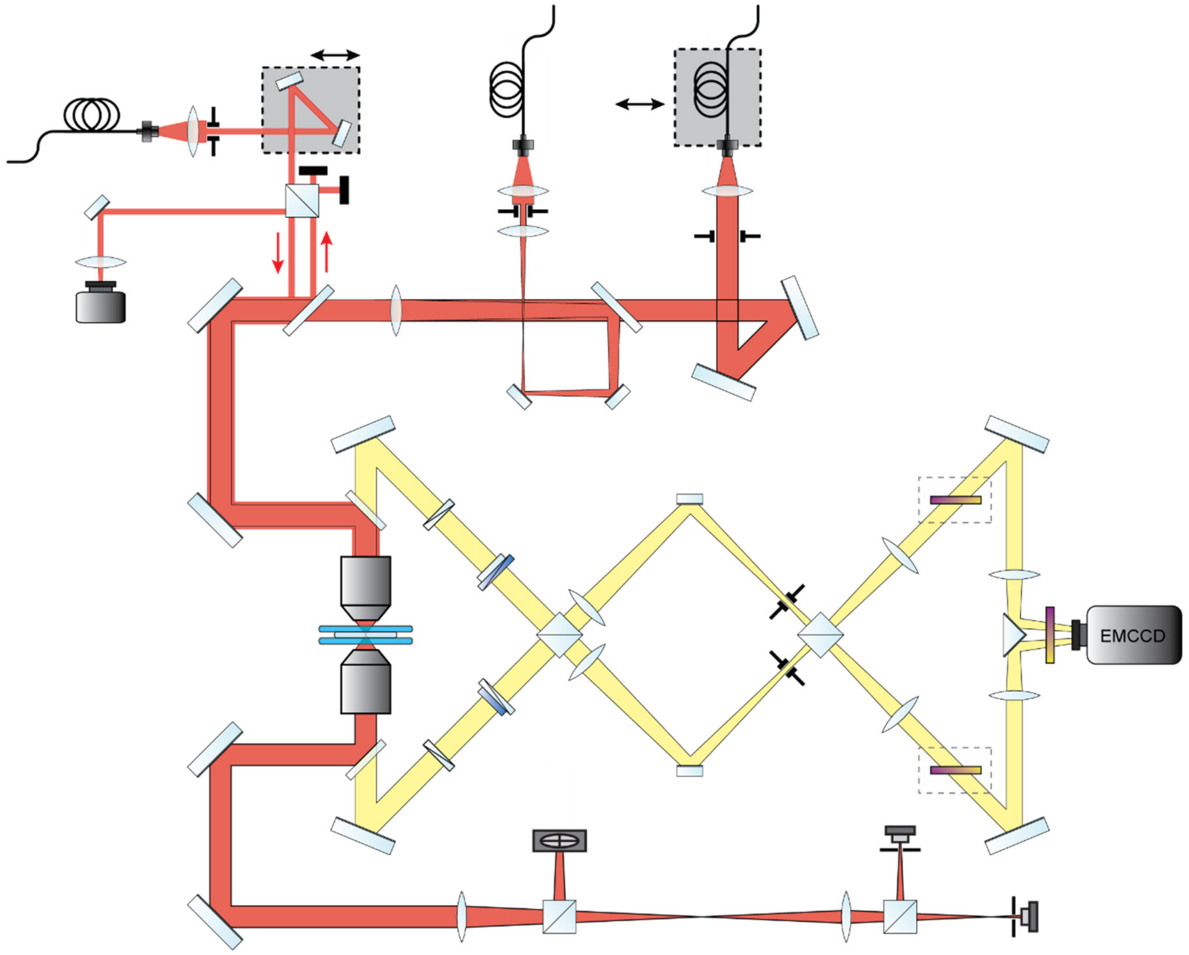

Supplementary Fig. 4: 4Pi-STORM optical system overview. The 4Pi fluorescence detection cavity, excitation light sources, objective alignment control system, and sample focus control system, together form the 4Pi-STORM microscope. This figure shows how these systems, shown in detail in Extended Data Fig. 1 and Supplementary Figs. 1-3, are integrated together on a single optical table. The system was constructed in the horizontal plane, on an optical table measuring 150cm x 120cm. The laser launch was built on a separate optical breadboard (not shown).

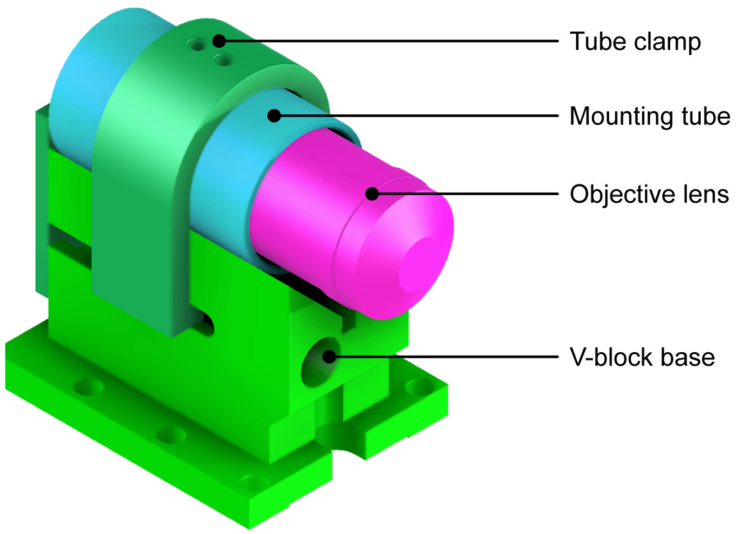

Supplementary Fig. 5: Fixed objective mount. Mechanical assembly drawing of the mounting system for the fixed objective lens (Objective A). The objective lens is mounted in a stainless steel threaded tube, which is supported by a machined aluminum V-block. The tube is clamped to its support using a U-clamp, tightened by two set screws at the top of the clamp. A mechanical stop at one end of the V-block sets the axial position of the mounting tube. This design allows the objective lens to be removed from the system, and replaced, with high positional reproducibility. A CAD file containing this drawing is available as Supplementary Data with this manuscript (see Supplementary Note 10).

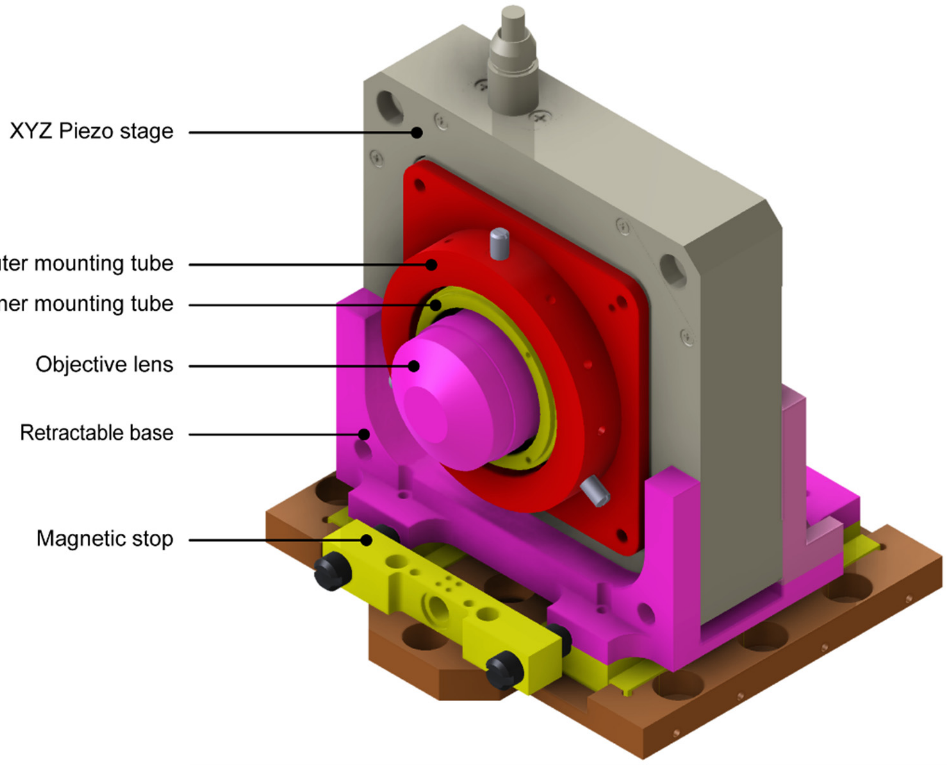

Supplementary Fig. 6: Movable objective mount. The position and orientation of the second objective lens (Objective B) is fully adjustable. The objective is mounting in stainless steel threaded tube, which itself is held within an outer mounting tube (colored red in the drawing) by six set screws. Adjustment of these screws allows coarse positioning of the objective, and adjustment of tip and tilt, during initial microscope alignment. The mounting assembly is held within the aperture of a three-axis piezoelectric stage, which provides fine translation in x, y, and z. The piezo stage is supported in a sliding mount, which can be translated by several centimeters for sample insertion and removal. A magnetic stop at one end of the translation range provides coarse focus adjustment for the movable objective. A CAD file containing this drawing is available as Supplementary Data with this manuscript (see Supplementary Note 10).

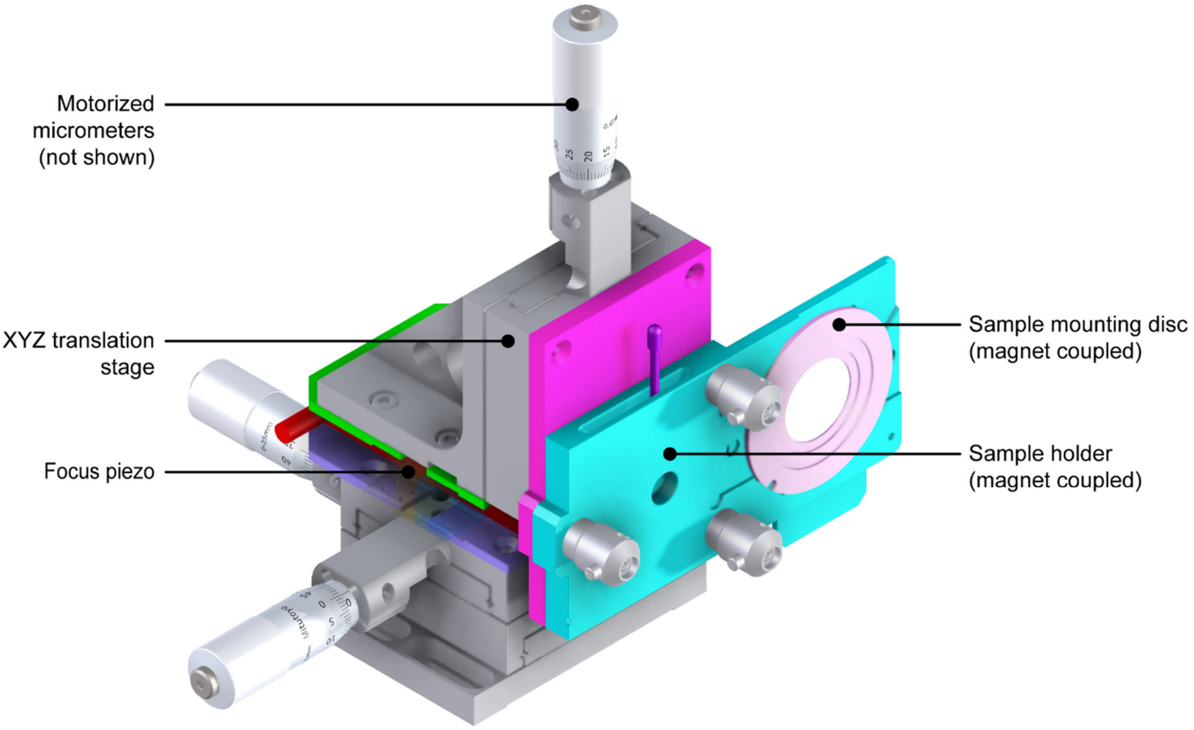

Supplementary Fig. 7: Sample mounting stage. The sample stage consists of a three-axis coarse translation stage, a linear piezoelectric stage, and a removable sample holder. Two axes of the XYZ translation stage are driven by motorized micrometer screws (not shown) to provide lateral sample translation. A manual micrometer screw provides coarse focus control, and a linear piezoelectric transducer provides fine focus control over a range of 30 micrometers. The sample coverslip is mounted on a stainless steel disc, which is magnetically coupled to the sample holder. The disc is oriented vertically, orthogonal to the objective lenses. The sample holder (colored cyan in the drawing) is also magnetically coupled to the translation stage, and may be released by shifting a lever. While coupled together, the sample holder and mounting disc may be inserted or removed from the microscope in order to change the sample. A CAD file containing this drawing is available as Supplementary Data with this manuscript (see Supplementary Note 10).

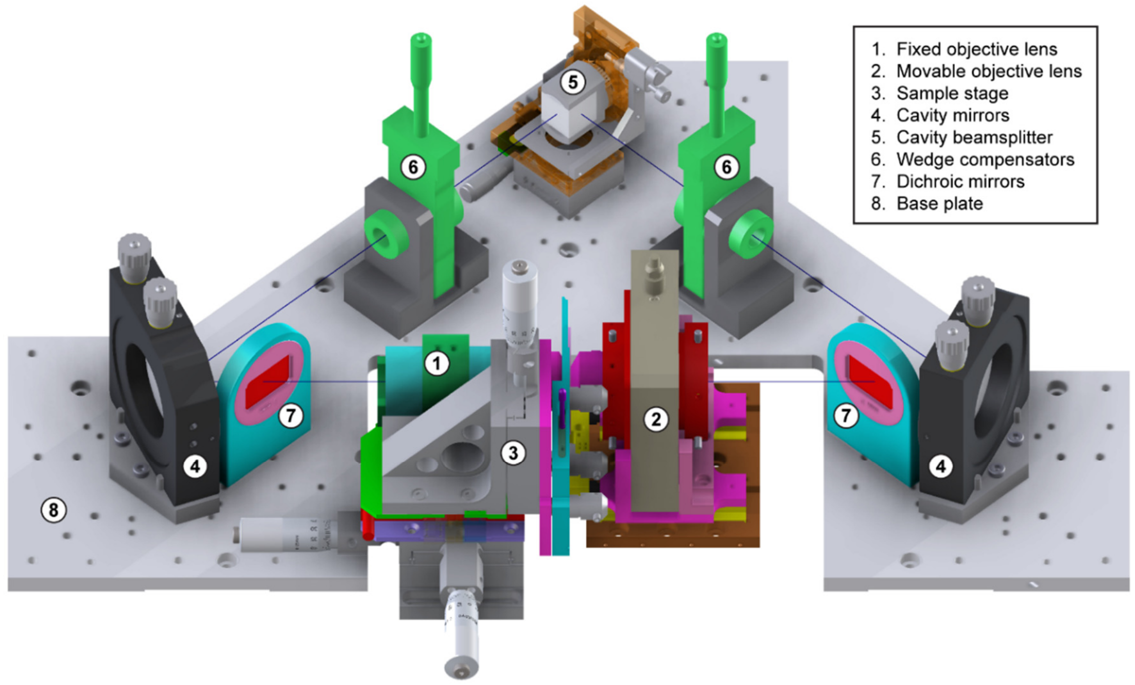

Supplementary Fig. 8: 4Pi-STORM interferometric cavity overview. Mechanical assembly drawing showing the 4Pi-STORM interferometric cavity. A machined base plate is used to assist in precise positioning of the cavity mirrors, dichroic mirrors, compensators, and beam splitter. The scaled drawing shows the positioning of the objective lenses and sample stage relative to the other components. Note that the quarter-wave plates are not shown in the drawing.

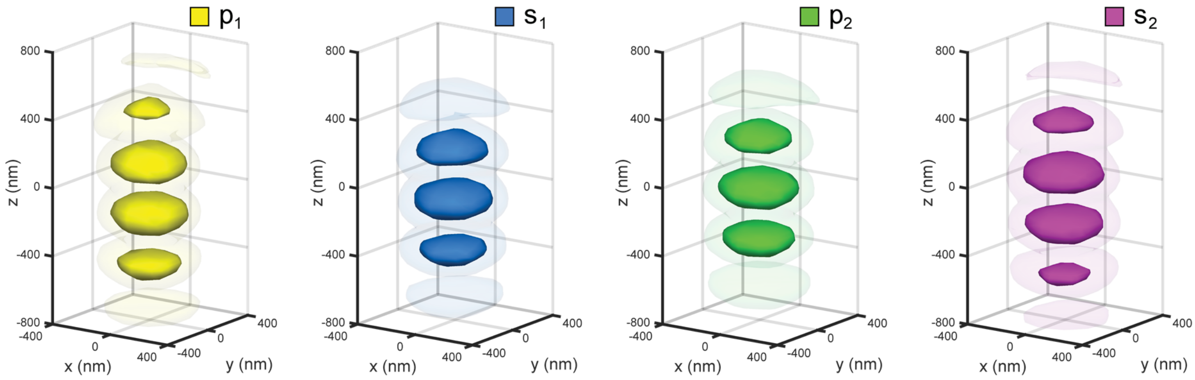

Supplementary Fig. 9: Individual channels of the 4Pi PSF. The four channels of the experimental 4Pi PSF shown in Fig. 1b and 1c, rendered as 3D isosurfaces at relative threshold levels of 35% and 13%, respectively.

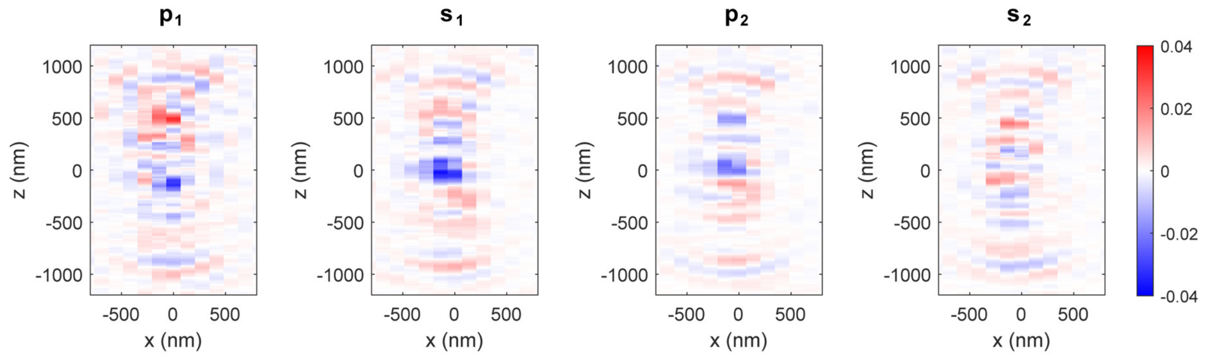

Supplementary Fig. 10: Comparison between PSF spline and measured bead scan. Normalized difference between the cubic spline PSF model obtained from one bead scan and the image stack from a second bead scan, for all detection channels. To match the second bead scan, the cubic spline PSF model was translated in *x*, *y*, and *z*, and its phase was shifted by 26°. An *x*-*z* cross-section through the center of the difference stack is shown for each detection channel. The maximum absolute deviation between the spline and the bead scan was 4% of the maximum intensity of the PSF.

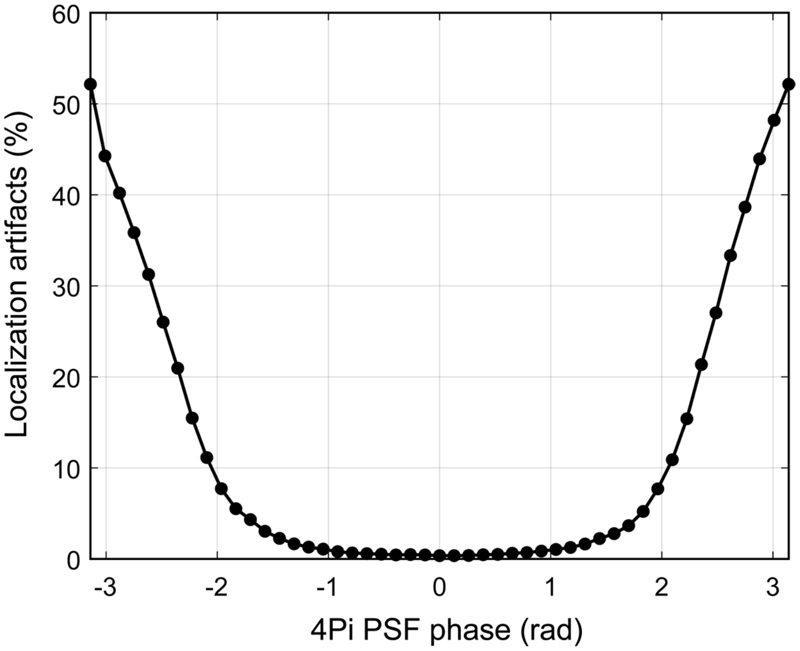

Supplementary Fig. 11: Artifact frequency vs. PSF phase error (simulation). A spline model of the 4Pi PSF was created from a fluorescent bead scan. Simulated fluorophore images were generated based on this model, with *z* coordinates evenly spaced over a range of 1 µm around the focal plane, and with a mean brightness of 8000 photons. The phase of the PSF model was then shifted (see Methods) and the model was used to fit the simulated fluorophore data for various PSF phase shifts. The fraction of localization artifacts was determined by generating a histogram of the difference of fitted and simulated z positions and marking all but the largest peak in the histogram as artifacts. This estimation is equivalent to the localization artifact estimation for experimental data. These results were also validated using an experimental dataset (Supplementary Fig. 12).

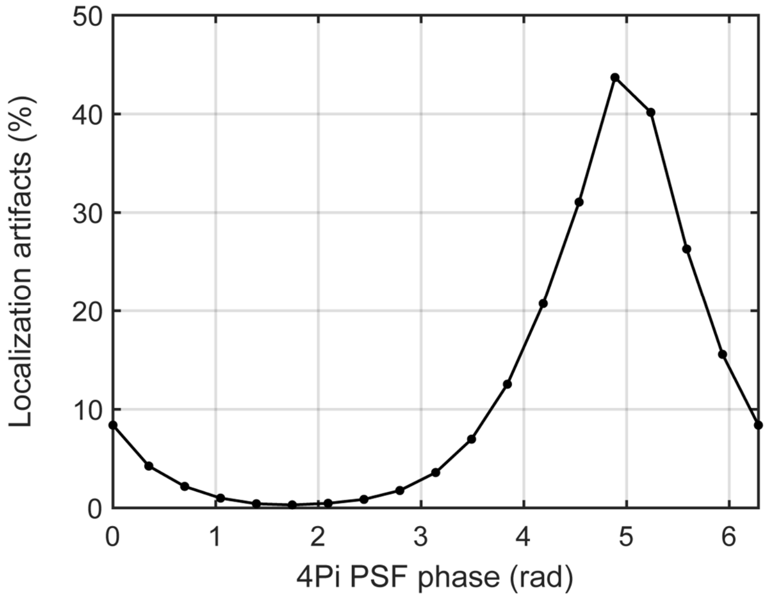

Supplementary Fig. 12: Artifact frequency vs. PSF phase error (experiment). Localization artifact frequency for an experimental dataset (Nup96 shown in Fig. 2) versus the phase of the cubic spline PSF model used to analyze the dataset. The PSF phase was numerically shifted over 18 steps, the dataset was analyzed with the PSF model calculated at each step, and the fraction of localization artifacts was determined from the localization results (black circles) as described in Supplementary Note 1. The plot shows that the artifact rate is close to zero at the global optimum PSF phase for the measurement, and remains low over a range of at least 2 radians centered at the best phase. At the opposite phase, the artifact rate approaches a maximum value of 50%.

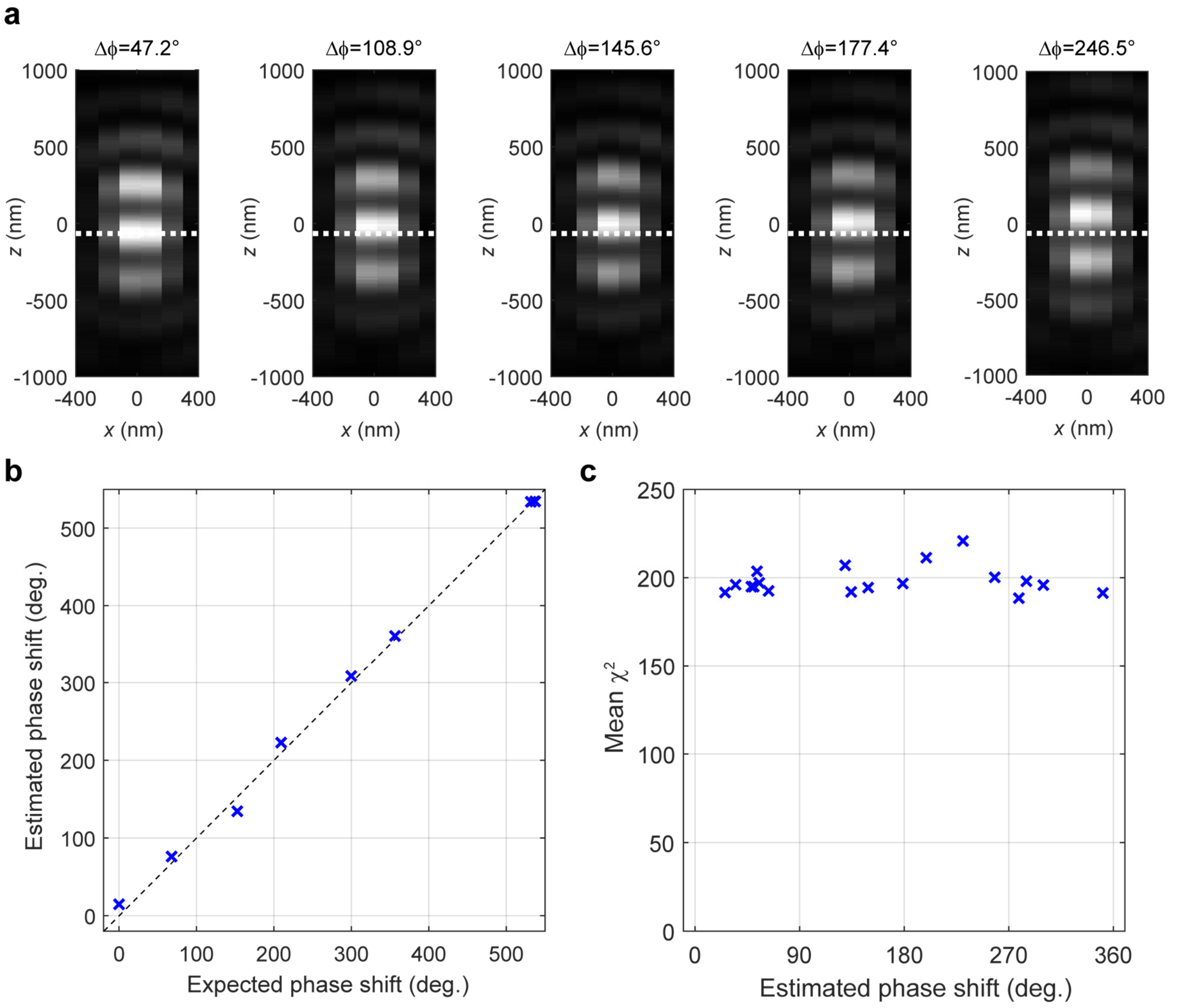

Supplementary Fig. 13: Validation of 4Pi PSF phase shift algorithm. A series of externally applied phase shift were introduced into the 4Pi cavity by manually changing the thickness of the BK7 glass wedge compensator in one arm of the cavity. This control experiment was used to verify the accuracy of the PSF phase estimation, and also the accuracy of the algorithm used to numerically shift the PSF phase. After each manual shift, a bead scan was recorded to measure the PSF, and the phase of the PSF was estimated using the spline fit approach. **(a)** Cross-section views (*x-z*) of the bead scans after each phase shift. The estimated phase shift is shown above the bead image. **(b)** Estimated phase (blue markers) at each shift position versus the expected phase (in degrees). A dashed line with a slope of 1 is plotted for reference. The results show that the estimated phase agrees well with the expected phase shift, within the precision of the micrometer screw on the compensator. The expected phase shift was calculated from the known relationship between the optical path length of the cavity and the glass wedge thickness. **(c)** For each phase estimation, the fit procedure returns a mean value for chi-square ($<\chi^2>$), which measures of how well the PSF model describes the data. A plot of $<\chi^2>$ vs. the estimated phase showed no dependence on the amount of phase shift, demonstrating that the numerical phase shift algorithm yields physically accurate estimates of the true PSF.

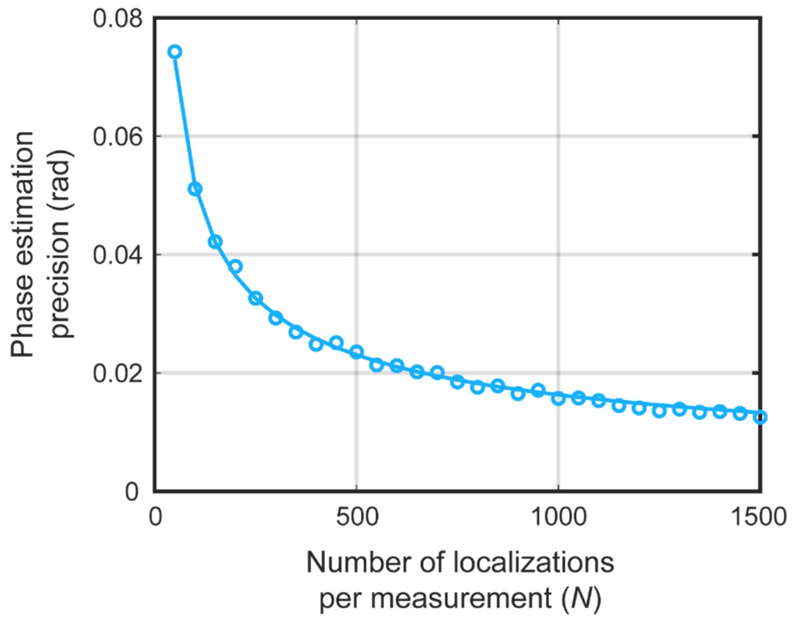

Supplementary Fig. 14: Precision of phase estimation. Precision of the PSF phase estimation as a function of the number of localizations used to estimate the phase. Simulated localization data was generated based on a PSF with a known phase shift, and then phase was then estimated using different numbers of input images. The simulated data featured emitter *z*-positions uniformly distributed over a 1 µm range around the focal plane, an emitter brightness of 8000 photons per event and a background signal of 10 photons per pixel. The simulation results (blue circles) show an improving precision as the number of localizations per measurement increases, as expected. The data were fit with a power law curve which scales as the square root of the number of input images (blue line), with a brightness- and background-dependent amplitude factor.

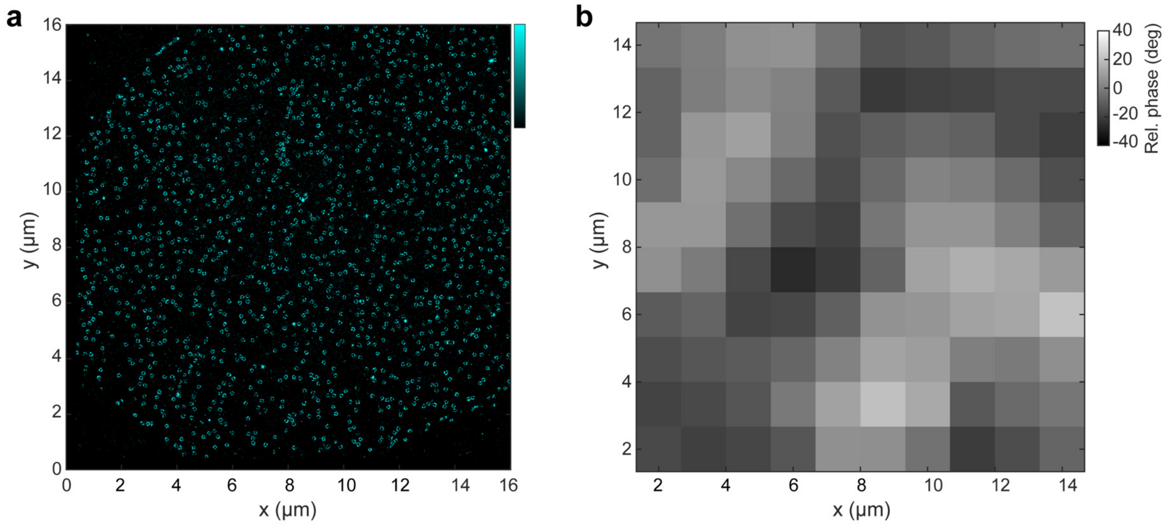

Supplementary Fig. 15: PSF phase variation across the field of view. Phase variation across the field of view of the 4Pi-STORM microscope. **(a)** The relative phase was determined by analyzing subsets of localizations from an image of nuclear pore complexes (Nup107) in a nuclear membrane which extends across a large area. **(b)** The localization data was subdivided into bins from sample regions 1.33 x 1.33μm in size, and the PSF phase was determined for each bin. Within the image, the phase was found to vary over a range of ± 40 degrees.

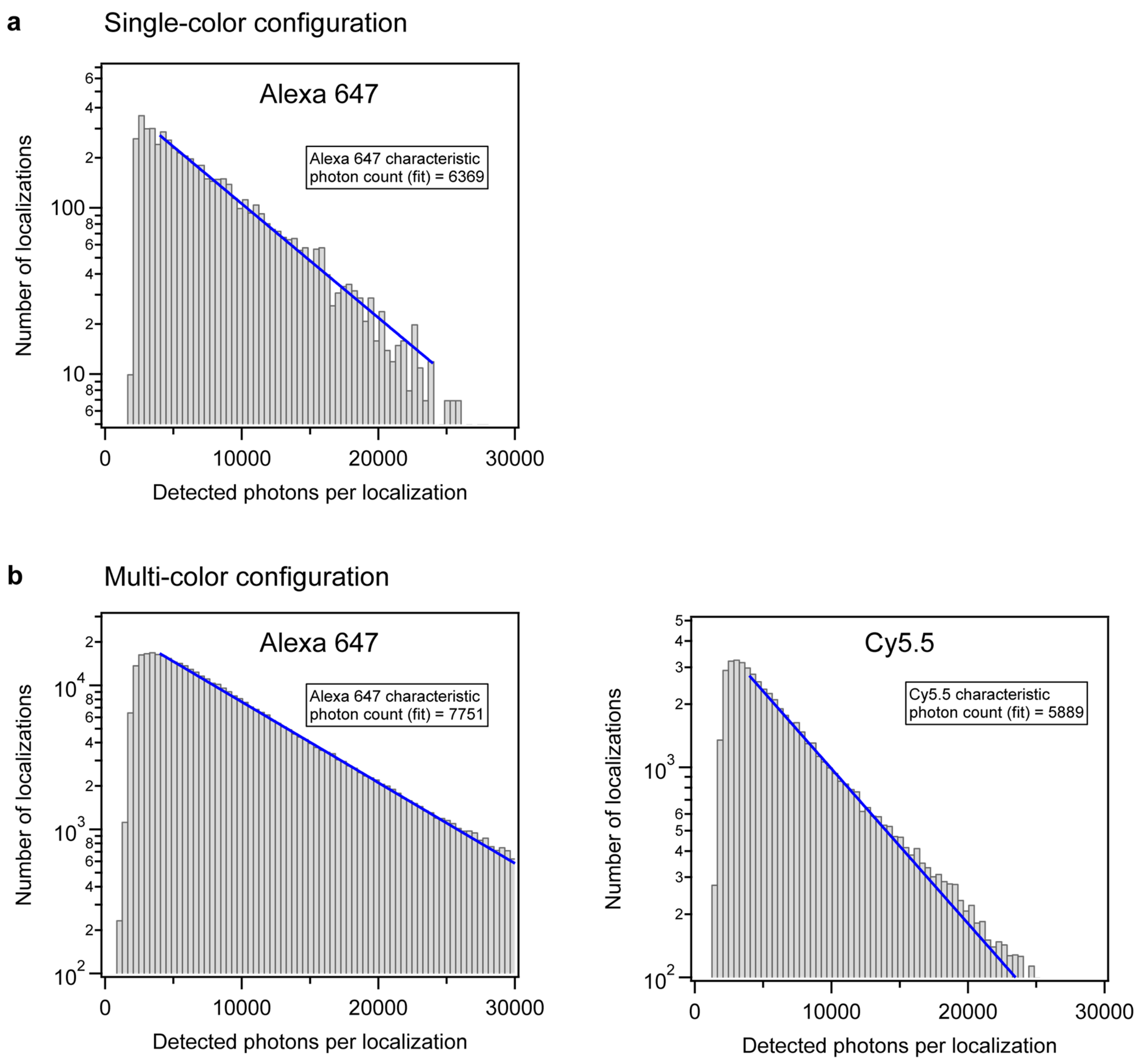

Supplementary Fig. 16: Number of photons detected per localization event. (a) With the microscope in the single-color detection configuration (see Methods), single molecules of Alexa Fluor 647-labeled double-stranded DNA, bound to a glass coverslip, were used to measure the localization precision of the 4Pi-STORM microscope (Fig. 3). A histogram of the number of photons per on-off switching event (grey bars) was fit with an exponential function (blue line) to determine the characteristic detected photon count. Events with fewer than 1500 photons were discarded from the analysis. The fit yields a characteristic photon count of 6369, and the mean value of the photon distribution was 8002. **(b)** For comparison, photon count histograms are also shown for Alexa Fluor 647 and Cy5.5 with the microscope in the multicolor detection configuration. Localization data are from the two-color 4Pi STORM image shown in Fig. 6. In this experiment, both Alexa Fluor 647 and Cy5.5 were bound to secondary antibodies. Exponential fits to the histograms (blue lines) yield a characteristic photon count of 7751 for Alexa Fluor 647, and 5889 for Cy5.5.

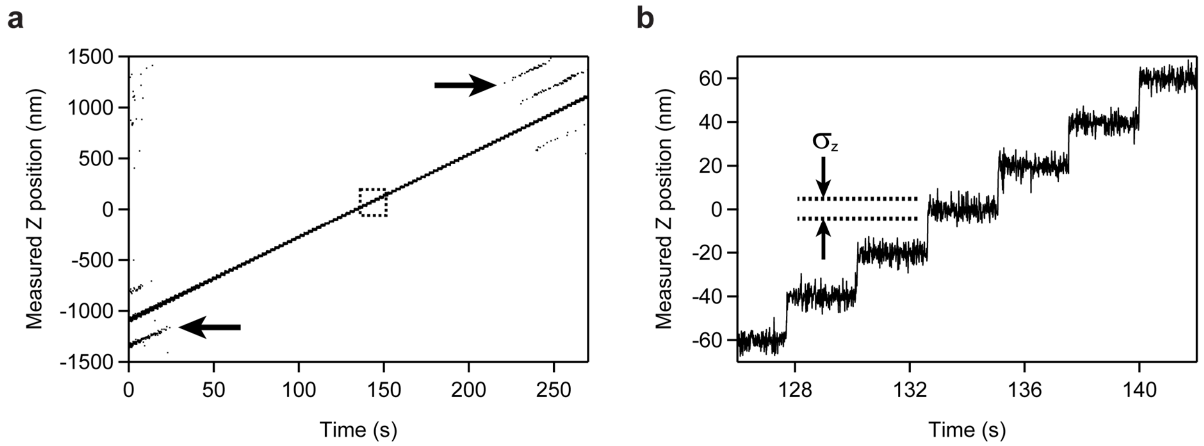

Supplementary Fig. 17: Bead step scan. A fluorescent bead (100 nm diameter) bound to a glass coverslip was scanned through the focal plane of the microscope in 20 nm steps, to measure the localization precision and the frequency of localization artifacts. The illumination was adjusted such that the number of detected photons per camera frame was similar to the brightness of a single fluorophore (~9200 photons per frame). For every image in the scan, the bead position was measured by fitting the image with the cubic spline PSF model. **(a)** Measured *z* position of the bead during the scan. At the beginning and end of the scan, localization artifacts are visible as periodic bands below and above the true position of the bead (arrows). These are localization errors (artifacts) which arise when the fit algorithm assigns the bead position to the wrong interference fringe of the PSF. The fraction of localization errors can be calculated by comparing the number of correctly localized events to the number of events in the bands above and below the central scan line. **(b)** Zoomed view of the boxed region in (a). The detail of the scan shows the individual steps. At each scan step, the localization precision was measured by calculating the point-to-point variation in the position measurement. Sample drift was estimated using a low-pass filter, and subtracted from the step scan data.

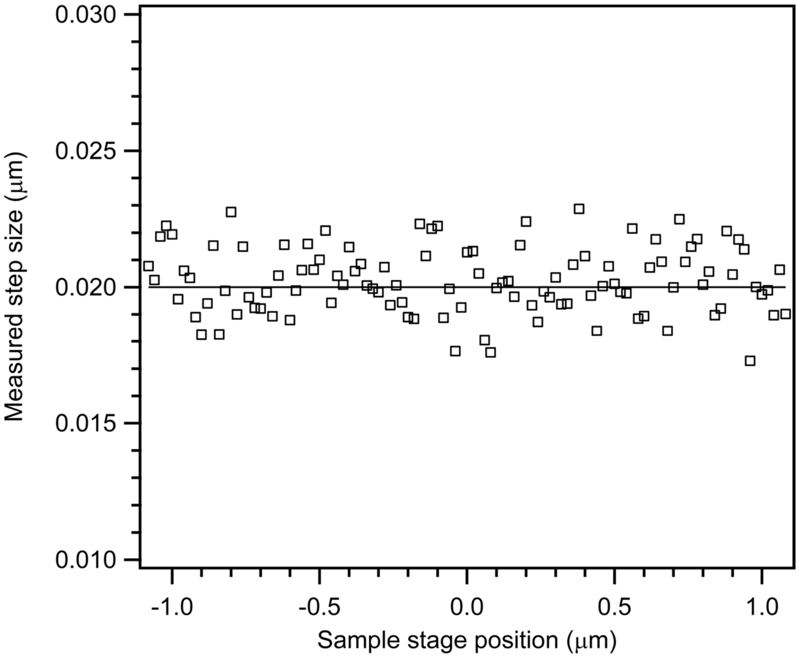

Supplementary Fig. 18: *z*-coordinate localization accuracy. A fluorescent bead (100 nm diameter) bound to a glass coverslip was scanned through the focal plane of the microscope in 20 nm steps, as in Supplementary Fig. 17. For this measurement, the illumination was adjusted such that more photons were detected from the bead (approximately 25000 per exposure). Here, the measured step sizes of the scan (black squares) are plotted vs. the piezo stage position during the scan, to test for bias in the localization procedure. Scatter in the data points is due to random measurement error at each time point. Within the measurement precision, the estimated step sizes do not deviate from the expected value (solid black line) over the full range of the scan, exhibiting no systematic bias with respect to the *z*-position of the emitter.

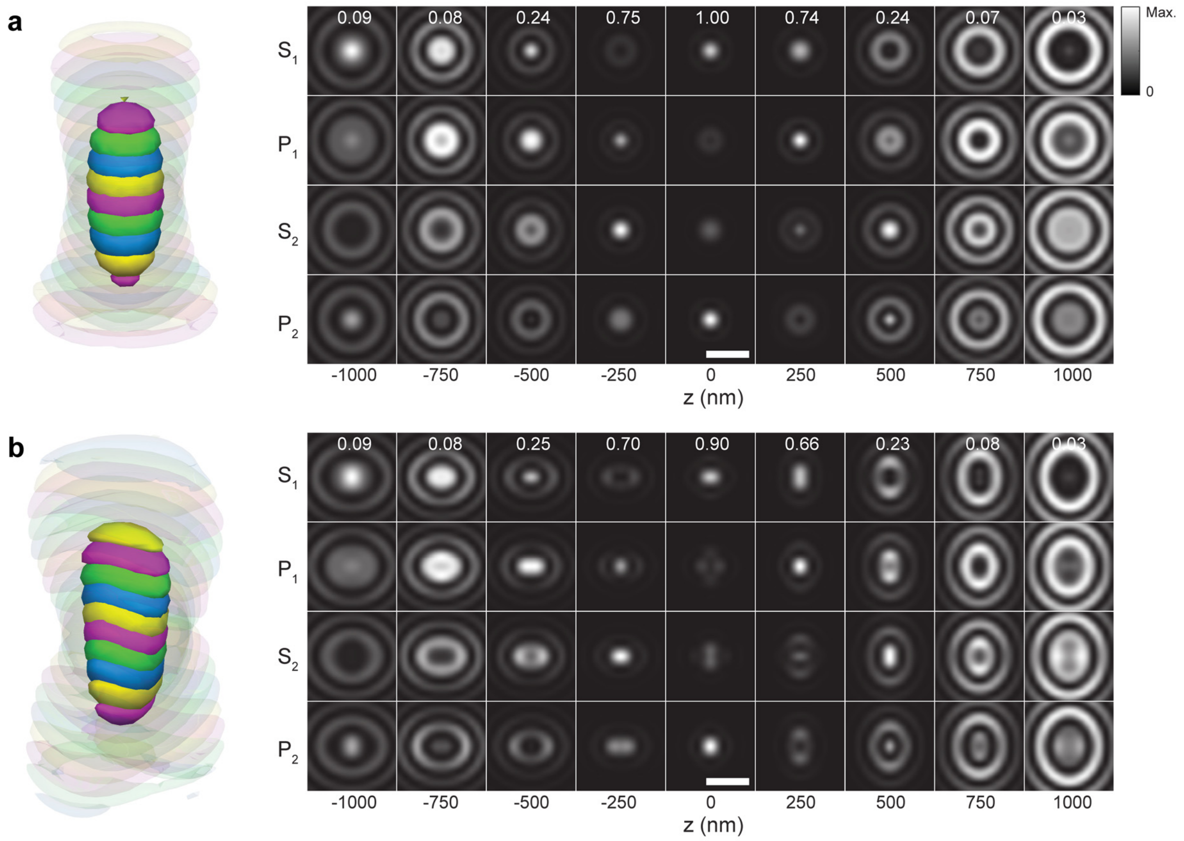

Supplementary Fig. 19: Simulated symmetric and astigmatic 4Pi PSFs. A matrix of *x*-*y* slices through the simulated symmetric (a) and the astigmatic (b) 4Pi PSFs used for the analysis shown in Fig. 4, at various *z*-positions relative to the focal plane, for the four image channels. Details of the PSF calculations are given in Supplementary Note 4. A 3D rendering of each PSF is shown to the left of the image matrix. For each z position, all four image channels were scaled to the same maximum value. The value of the maximum, relative to the maximum of the symmetric 4Pi PSF at z=0, is shown at the top of each column. Scale bar: 500 nm.

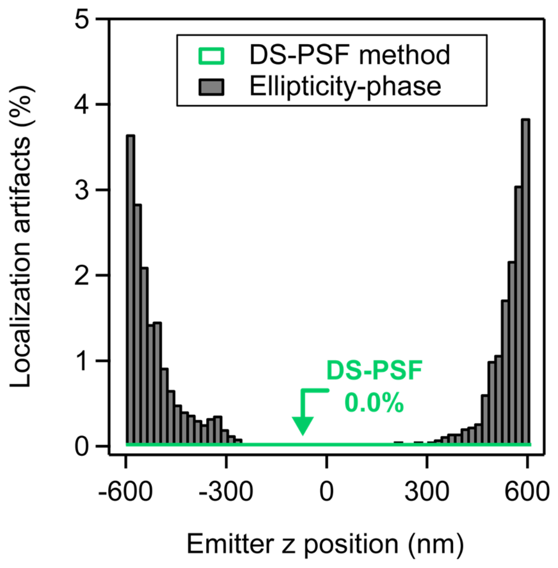

Supplementary Fig. 20: Comparison with Astigmatic 4Pi (ellipticity-phase) analysis. Artifact frequency as a function of the emitter *z*-coordinate, corresponding to the simulated data sets evaluated in Fig. 4. Simulated fluorophore images were generated based on the astigmatic 4Pi PSF and the symmetric 4Pi PSF. Data based on the astigmatic PSF were analyzed with the ellipticity-phase method, and data based on the symmetric PSF were analyzed with the dynamic spline PSF method. The simulated datasets were based on experimentally realistic photon counts with a mean of 8000 photons per event, and the size of the emitter cutout region was 13x13 pixels (138 nm pixel size). Both methods obtain a low rate of localization artifacts, with the ellipticity-phase analysis reaching an artifact frequency of ~4% for positions 600 nm from the focal plane. The artifact frequency for the dynamic spline PSF fit was 0% over the range tested.

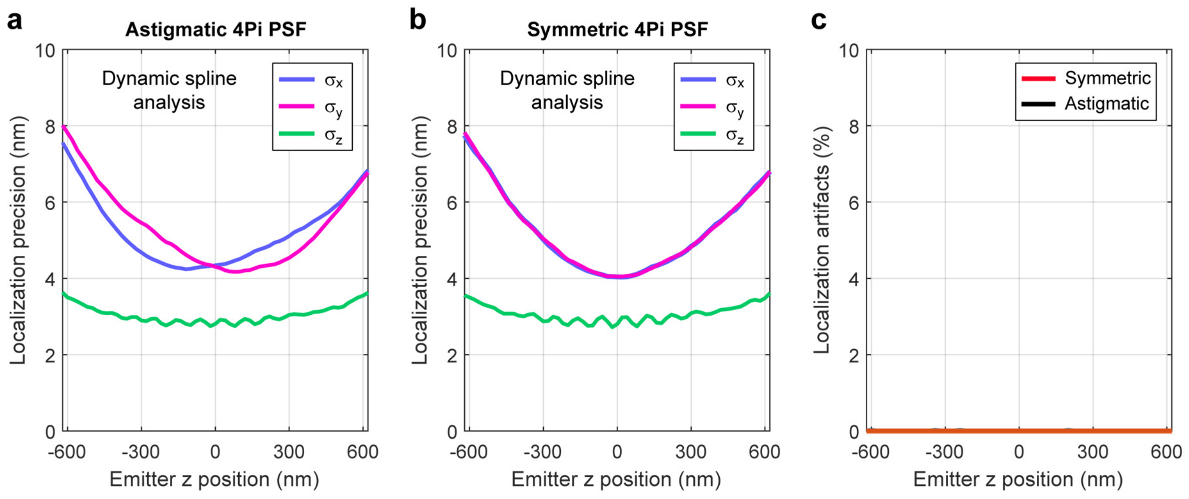

Supplementary Fig. 21: Spline analysis with Astigmatic or Symmetric 4Pi PSF. Localization precision (a,b) and artifact rate (c) for a simulation of emitters at different *z* positions, using either the astigmatic or the symmetric 4Pi PSF (Fig. 4) to generate and fit the data. Here, both datasets were analyzed with the dynamic spline method, instead of using the ellipticity-phase analysis for the astigmatic PSF. The results show that the localization precision is comparable for the two PSFs with the new analysis, with the symmetric 4Pi PSF giving slightly better results due to its more compact shape. The artifact fraction was < 0.1% over the entire *z*-coordinate range tested. In the simulated data, there were on average 8000 photons per localization, and 10 background photons per pixel.

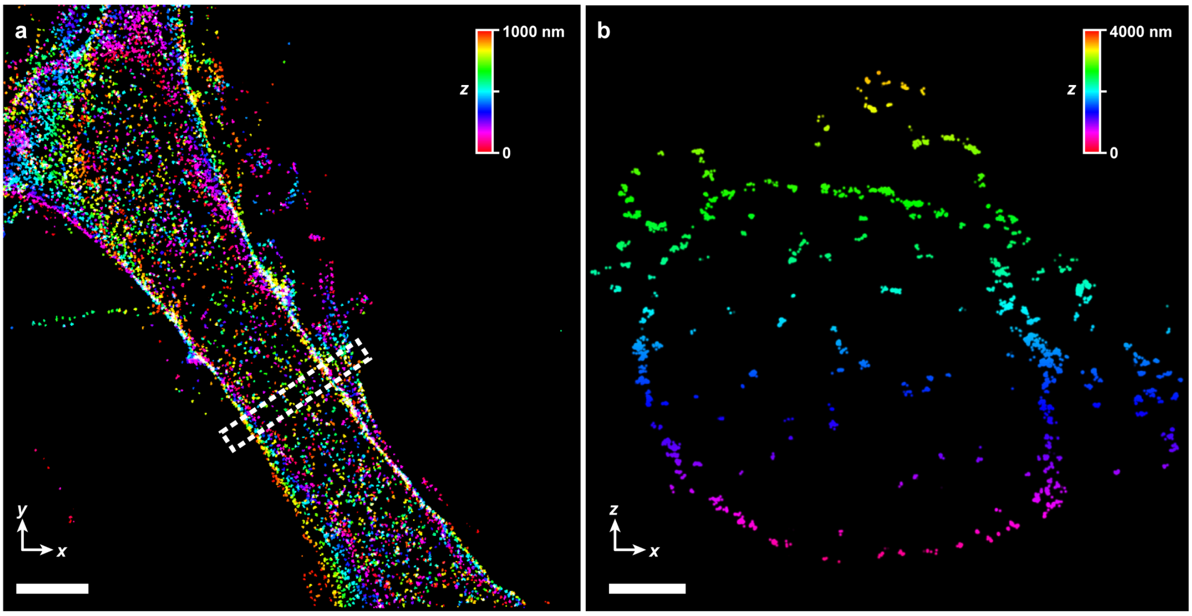

Supplementary Fig. 22: Beta-II spectrin in a thick neuronal cell. A primary neuronal cell, several micrometers in thickness, labeled with antibodies against beta-II spectrin. **(a)** An average projection through a top view (*x*-*y*) of the cell, with the viewplane positioned in the center of the sample. The image shows a high density of spectrin at the surface, and a low density inside the cytoplasm. Scale bar: 2 µm. **(b)** A cross-section view (*x*-*z*) through the boxed region in (a), showing the circular profile of the cell. Several neuronal processes are visible, clustered around the surface of the larger cell. This dataset was obtained by shifting the sample with respect to the objective lenses during the recording, in ten steps separated by 400 nm, for a total imaging depth of approximately 5 micrometers. Notably, localization artifacts are not evident in the image, despite the large extent of the sample in the *z*-dimension. Scale bar: 500 nm.

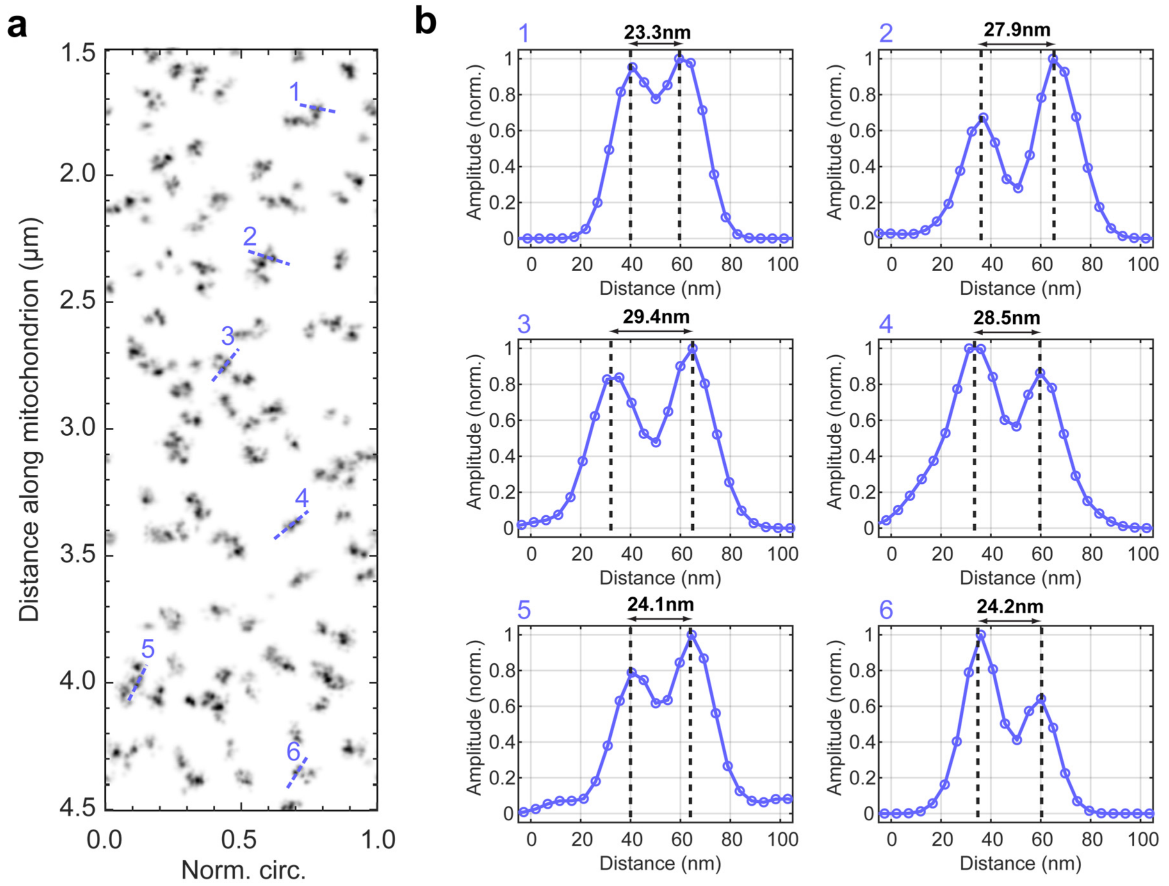

Supplementary Fig. 23: Measurements of Mic60 organization in a U-2 OS cell. (a) A 3 µm long section of an unwrapped view of Mic60 in a U-2 OS cell, showing the location of six line profiles (blue, dotted lines) that cross neighboring Mic60 spots in different Mic60 puncta. **(b)** Corresponding line profiles through the unwrapped views and measured distances between peaks. Note that the unwrapping of a curved surface onto a rectangle slightly distorts the scale (average circumference within the section was used).

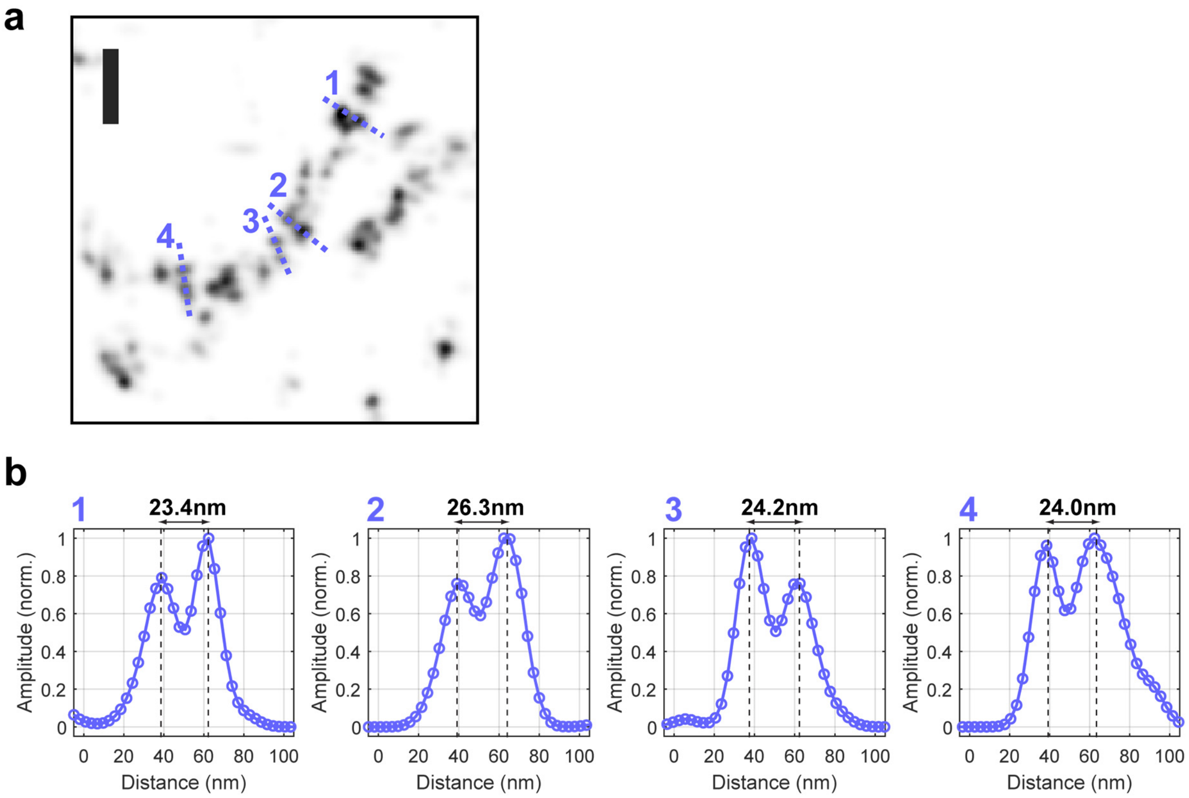

Supplementary Fig. 24: Measurements of Mic60 organization in a COS-7 cell. (a) Zoomed view of an unwrapped section of mitochondrial Mic60 in a COS-7 cell showing the location of four line profiles (blue, dotted lines) that are perpendicular to the orientation of the apparent Mic60 stripe. Scale bar: 100 nm **(b)** Corresponding line profiles through the unwrapped views and measured distances between peaks. Note that the unwrapping of a curved surface onto a rectangle slightly distorts the scale (average circumference within the section was used).

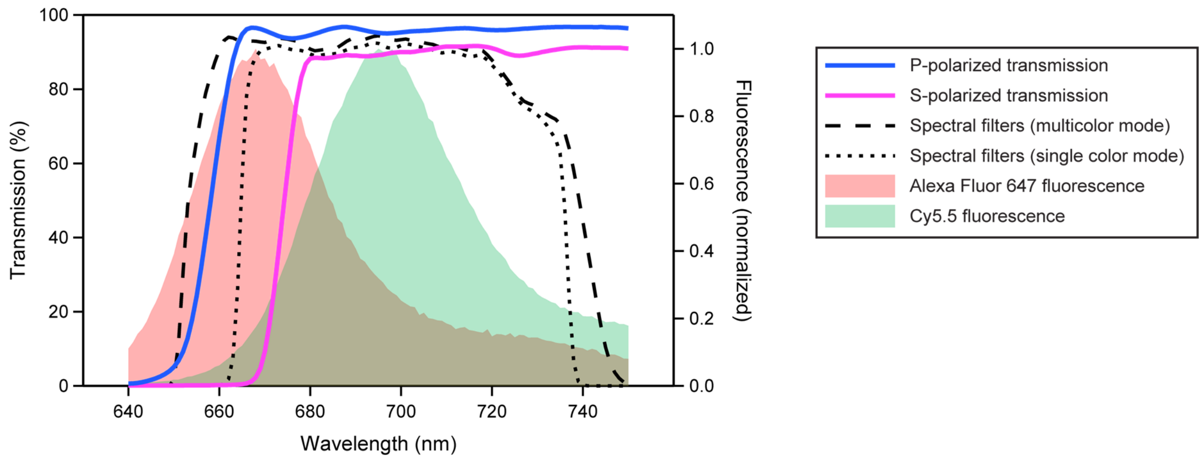

Supplementary Fig. 25: Polarization-specific spectral filter for multicolor imaging. Discrimination of Alexa Fluor 647 and Cy5.5 fluorophores is achieved using a spectral long-pass filter which has a different cutoff wavelength for S- and P- polarized light. The plot shows the fluorescence emission spectra of the two dyes (shaded areas) whose emission maxima are separated by approximately 30 nm. A dichroic mirror placed in each detection path (Extended Data Fig. 1), placed at an angle of ~34 degrees with respect to the fluorescence beam, has a different transmission spectrum for each polarization (blue and magenta solid lines). Specifically, the S-polarized cutoff wavelength is red-shifted by ~15 nm with respect to the cutoff for P-polarized light. This filter configuration introduces a significant difference in the ratios of detected S- and P-polarized photons for Alexa Fluor 647 and Cy5.5, allowing the two fluorophores to be distinguished by this parameter. Also shown are the combined spectra for the other dichroic mirrors and spectral filters in the system (dashed lines), for the single color and multicolor configurations (see Methods).

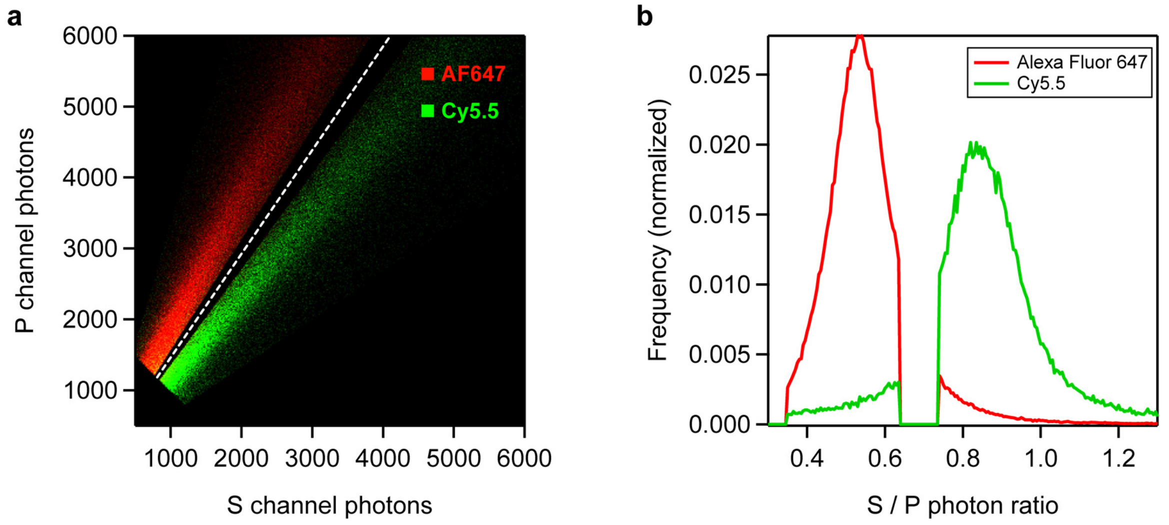

Supplementary Fig. 26: Color discrimination and crosstalk estimation. Control experiment using samples labeled with a single color (Alexa 647 or Cy5.5) and imaged with the two-color imaging configuration (dichroic mirrors DM3 in the detection path, Extended Data Fig. 1). **(a)** Two-dimensional histogram showing the number of photons detected in the P-polarized channels ($p_1 + p_2$) vs. the number detected in the S-polarized channels ($s_1 + s_2$) for each localization event, for the two control samples (Alexa 647 and Cy5.5). Events for each fluorophore appear in two groups, separated by the ratio of S to P photons. A white dashed line indicates the threshold value used for initial identification of the two fluorophores in the multicolor analysis. **(b)** Histogram of S/P photon ratio for the two control samples, showing the expected mis-identification fraction (crosstalk). Events with an S/P ratio < 0.7 are identified as Alexa 647, and with > 0.7 as Cy5.5. Events with high ambiguity in color identity (S/P ratio between 0.64 and 0.74) were filtered out to reduce crosstalk. Overall, 93% of localization events are correctly identified.

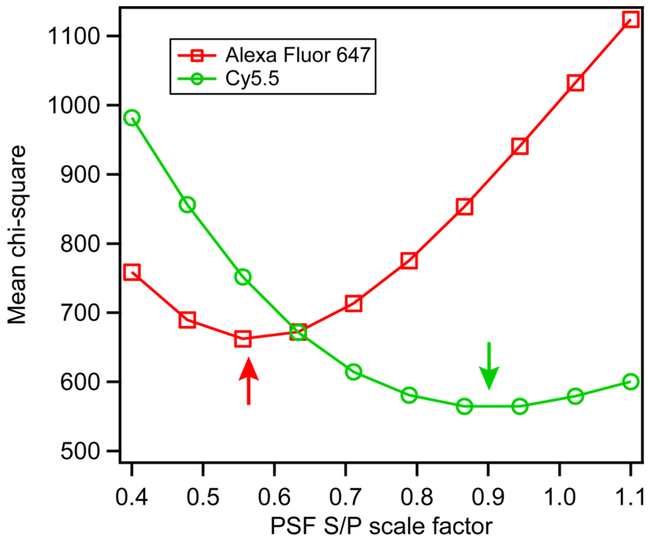

Supplementary Fig. 27: Multicolor PSF S/P scale factor determination. The correct scaling of the PSF S- and P-channel amplitudes may be determined from the localization data. In this example, localizations from the two-color dataset shown in Fig. 6 (Mic60 & DNA) were initially assigned colors based on the ratio of detected S-channel photons to P-channel photons, using a simple threshold of 0.7 (see Supplementary Fig. 26). Next, a series of test PSFs were generated by rescaling the S-channel of the PSF spline relative to the P-channel, over a range of 0.4 to 1.1. Each color group of localizations was fit with the test PSFs, and for each group the mean chi-square of the fits is plotted vs. the channel scaling factor. The minimum of each curve (arrows) reveals the PSF S- to P-channel amplitude ratio for each dye which best corresponds to the experimental data. In this case, the optimal scaling factor was 0.57 for Alexa Fluor 647, and 0.91 for Cy5.5, determined with a parabolic fit.

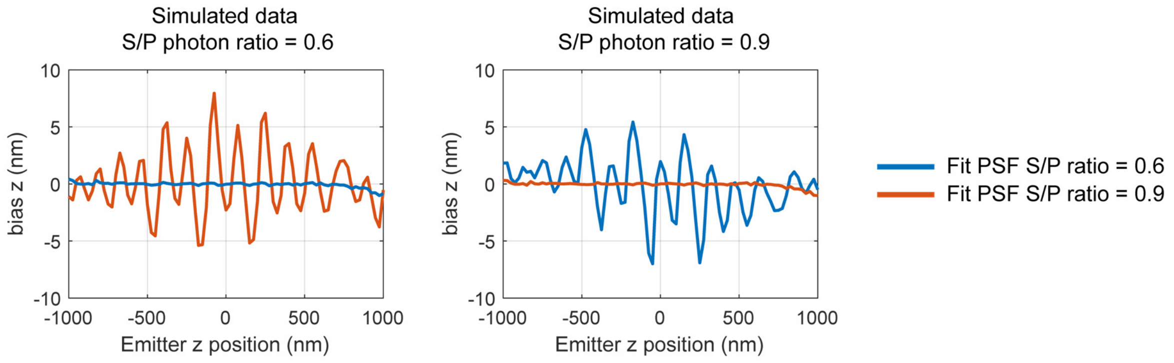

Supplementary Fig. 28: *z*-coordinate bias due to PSF model scaling (simulation). Simulation of the error in the fit results which would be introduced if the incorrectly scaled PSF is used for fitting in multicolor 4Pi-STORM analysis. Simulated emitter images were generated for Alexa Fluor 647 and Cy5.5, having an S- to P- polarized photon ratio of 0.6 and 0.9, respectively. Both sets of data were then fit with the correctly scaled PSF (ratio=0.6 for Alexa Fluor 647 and ratio=0.9 for Cy5.5) and the incorrectly scaled PSF. For each fluorophore, the error in the estimated *z*-coordinate is plotted as a function of the *z*-coordinate of the emitter.

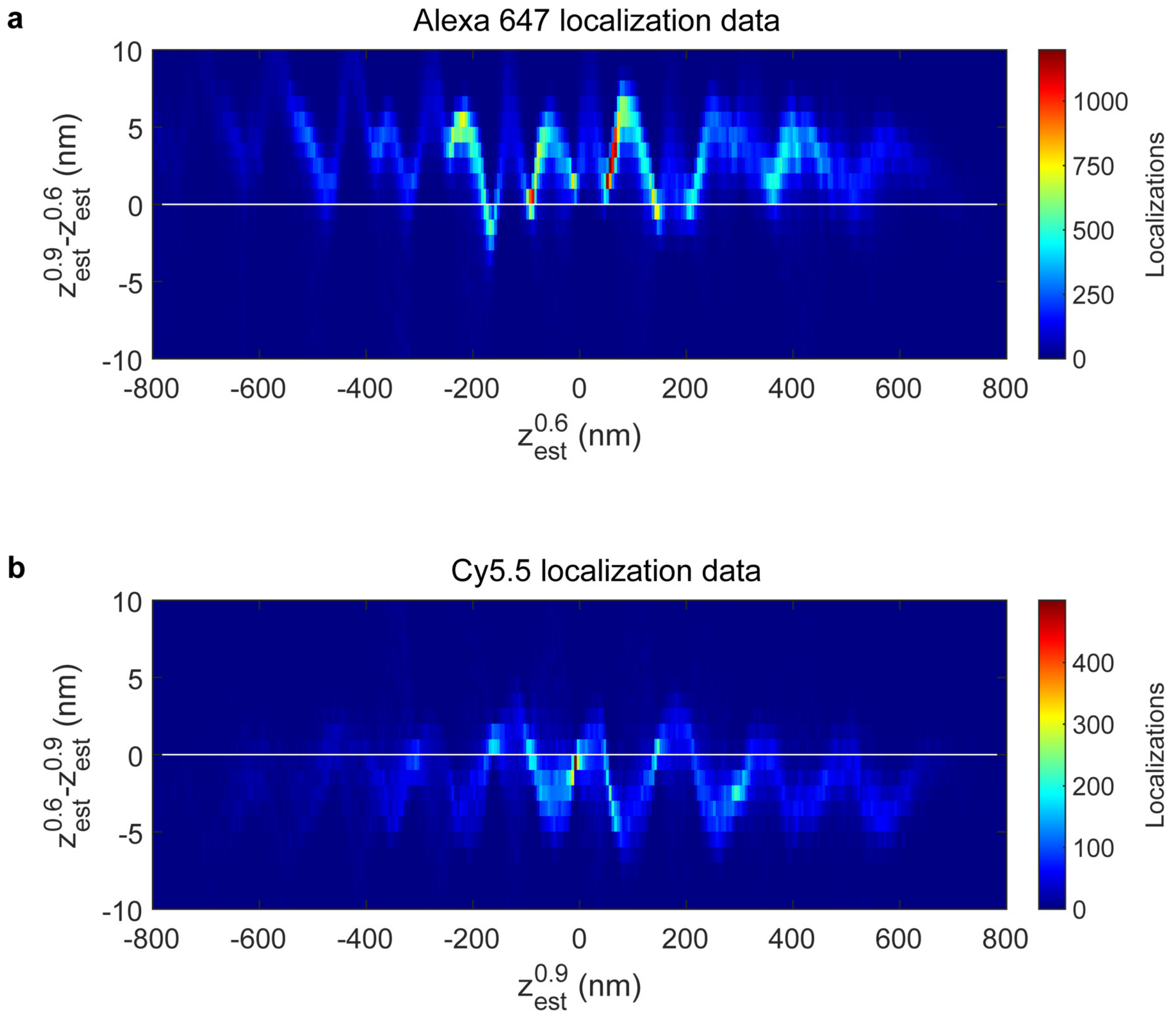

Supplementary Fig. 29: *z*-coordinate bias due to PSF model scaling (experiment). Experimental test of the error introduced when a PSF model with the incorrect S/P channel amplitude ratio is used to fit the emitter images. Localizations from a two-color 4Pi-STORM dataset were sorted into two groups, corresponding to the two fluorophores (Alexa Fluor 647 and Cy5.5), based on the measured ratio of S- to P- polarized detected photons. Next, each group was fit with both the correctly scaled PSF (ratio=0.6 for Alexa 647 and ratio=0.9 for Cy5.5) and the incorrectly scaled PSF. For each group, the discrepancy in the *z*-coordinate between the two fits is plotted as a function of the estimated *z*-coordinate of the emitter obtained by the fit with the correctly scaled PSF model. This amounts to a measurement of the *z*-bias error which would be introduced by using the wrong PSF. **(a)** *z*-bias error for Alexa Fluor 647, as a function of emitter *z* position. **(b)** *z*-bias error for Cy5.5, as a function of emitter z position.

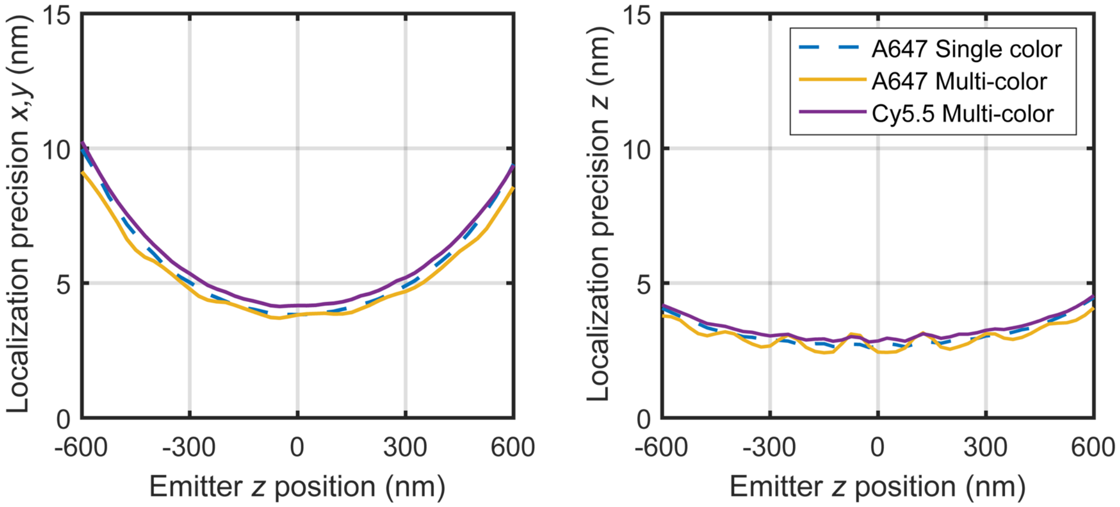

Supplementary Fig. 30: Single color vs. multicolor localization precision. Simulation of localization precision for the fluorophores used in the single color and multicolor imaging configurations. Images of fluorescent emitters were simulated over a range of *z* positions, using mean photon counts and background levels obtained for Alexa Fluor 647 and Cy5.5 in experimental datasets (samples 6 and 7, Supplementary Table 1). On average, the simulated localization uncertainty was 10% higher for Cy5.5 as compared to Alexa Fluor 647, due to the lower number of photons detected. Also, for multicolor imaging of Alexa Fluor 647, the simulated *z* localization precision is observed to modulate with an amplitude of approx. 1 nm close to the focal plane. This variation arises due to the uneven distribution of fluorescence between the S- and P- channels in this configuration.

Supplementary Tables

Supplementary Table 1: 4Pi-STORM localization data statistics

Sample number	Sample description	Figure	Number of localizations (1)	Mean photons per event (2)	Mean event duration (frames) (3)	Mean background photons per frame (4)
1	Nup96 in U2-OS cell	2	27267	11923	1.4	17
2	Alexa Fluor 647 - DNA	3	14330	8078	1.5	7
3	Beta-II Spectrin in Neuron	5	391994	10071	2.2	26
4	Beta-II Spectrin in Neuron (thick sample)	Suppl. Fig. 22	242090	9246	1.8	30
5	Mic60 in U2-OS cell	6	240711	11989	1.5	52
6	Mic60 in COS-7 cell	6	302018	10002	1.8	31
7	Mic60 (AF647) and DNA (Cy5.5) in COS-7 cell	6	590623 (AF647) 138855 (Cy5.5)	10066 (AF647) 8071 (Cy5.5)	1.6 (AF647) 1.7 (Cy5.5)	26 (P) 16 (S)

Notes:

(1) Number of localizations in the full dataset, after localization filters have been applied (see Supplementary Table 2).

(2) Photons per localization event, calculated as the total of all photons detected per on-off fluorophore switching event, summed over the detection channels and the duration of the event. This value is calculated based on the localization event list after localization filters have been applied.

(3) Mean duration of the switching events, measured in camera exposures. The camera frame rate was 101.8 Hz for all measurements, and the exposure time was 9.3 ms.

(4) Mean background signal level per pixel, per detection channel, per camera exposure. Note that the background level is different in the S- and P-polarized detection channels for the multicolor dataset.

Supplementary Table 2: 4Pi-STORM localization filter parameters

Filter parameter	Min. value	Max. value	Unit
Gaussian peak fit width, summed channels (1)	0.8	3.0	pixels
Total photons per event, summed channels (2)	1500.0		photons
Switching event duration		5	camera frames
S / P photon ratio (multicolor only)	0.35	1.5	

Notes:

(1) The four detection channels were transformed to a common coordinate system and summed (see Methods). Each peak was fit with a 2D Gaussian function. This filter is applied to the width (standard deviation) of the Gaussian peak fit results.

(2) Photons per localization event, calculated as the total of all photons detected per on-off fluorophore switching event, summed over the detection channels and the duration of the event.

Supplementary References

1. Balzarotti, F. et al. Nanometer resolution imaging and tracking of fluorescent molecules with minimal photon fluxes. *Science* **355**, 606-612 (2017).
2. Richards, B., Wolf, E. & Gabor, D. Electromagnetic diffraction in optical systems, II. Structure of the image field in an aplanatic system. *Proceedings of the Royal Society of London. Series A. Mathematical and Physical Sciences* **253**, 358-379 (1959).
3. Gibson, S.F. & Lanni, F. Experimental test of an analytical model of aberration in an oil-immersion objective lens used in three-dimensional light microscopy. *J. Opt. Soc. Am. A* **9**, 154-166 (1992).
4. Leutenegger, M., Rao, R., Leitgeb, R.A. & Lasser, T. Fast focus field calculations. *Optics Express* **14**, 11277-11291 (2006).
5. Aguet, F., Geissbühler, S., Märki, I., Lasser, T. & Unser, M. Super-resolution orientation estimation and localization of fluorescent dipoles using 3-D steerable filters. *Optics Express* **17**, 6829-6848 (2009).
6. Huang, F. et al. Ultra-High Resolution 3D Imaging of Whole Cells. *Cell* **166**, 1028-1040 (2016).
7. Zhang, Y. et al. Nanoscale subcellular architecture revealed by multicolor three-dimensional salvaged fluorescence imaging. *Nature Methods* **17**, 225-231 (2020).
