# Supplementary material for: Optimal precision and accuracy in 4Pi-STORM using dynamic spline PSF models
Source: Nat Methods. 2022 May 16;19(5):603–12. doi: 10.1038/s41592-022-01465-8 (PMC9119851; doi:10.1038/s41592-022-01465-8)
Supplement: Supplementary file 2 — Reporting Summary [file 41592_2022_1465_MOESM2_ESM.pdf]

nature research

Corresponding author(s): Mark Bates and Stefan W. Hell

Last updated by author(s): Jan 30, 2022

Reporting Summary

Nature Research wishes to improve the reproducibility of the work that we publish. This form provides structure for consistency and transparency in reporting. For further information on Nature Research policies, see our Editorial Policies and the Editorial Policy Checklist.

Statistics

For all statistical analyses, confirm that the following items are present in the figure legend, table legend, main text, or Methods section.

n/a	Confirmed	
☒	☐	The exact sample size (*n*) for each experimental group/condition, given as a discrete number and unit of measurement
☒	☐	A statement on whether measurements were taken from distinct samples or whether the same sample was measured repeatedly
☒	☐	The statistical test(s) used AND whether they are one- or two-sided<br>*Only common tests should be described solely by name; describe more complex techniques in the Methods section.*
☒	☐	A description of all covariates tested
☒	☐	A description of any assumptions or corrections, such as tests of normality and adjustment for multiple comparisons
☐	☒	A full description of the statistical parameters including central tendency (e.g. means) or other basic estimates (e.g. regression coefficient) AND variation (e.g. standard deviation) or associated estimates of uncertainty (e.g. confidence intervals)
☒	☐	For null hypothesis testing, the test statistic (e.g. *F*, *t*, *r*) with confidence intervals, effect sizes, degrees of freedom and *P* value noted<br>*Give P values as exact values whenever suitable.*
☒	☐	For Bayesian analysis, information on the choice of priors and Markov chain Monte Carlo settings
☒	☐	For hierarchical and complex designs, identification of the appropriate level for tests and full reporting of outcomes
☒	☐	Estimates of effect sizes (e.g. Cohen's *d*, Pearson's *r*), indicating how they were calculated

Our web collection on statistics for biologists contains articles on many of the points above.

Software and code

Policy information about availability of computer code

Data collection	LabVIEW 2016
Data analysis	IDL 8.5.1, Matlab 2018B, and custom software available at www.github.com/gpufit/gpufit, and www.github.com/gpufit/gpuspline

For manuscripts utilizing custom algorithms or software that are central to the research but not yet described in published literature, software must be made available to editors and reviewers. We strongly encourage code deposition in a community repository (e.g. GitHub). See the Nature Research guidelines for submitting code & software for further information.

Data

Policy information about availability of data

All manuscripts must include a data availability statement. This statement should provide the following information, where applicable:

- Accession codes, unique identifiers, or web links for publicly available datasets
- A list of figures that have associated raw data
- A description of any restrictions on data availability

A sample dataset which may be used for demonstrating the analysis methods described above, and mechanical drawings (CAD files) specifying custom microscope components, are provided with the manuscript as Supplementary Data files (see Supplementary Notes 9 10). All data that support the findings of this study are available from the corresponding authors M.B. and S.W.H. upon request.

Field-specific reporting

Please select the one below that is the best fit for your research. If you are not sure, read the appropriate sections before making your selection.

☒ Life sciences ☐ Behavioural & social sciences ☐ Ecological, evolutionary & environmental sciences

For a reference copy of the document with all sections, see nature.com/documents/nr-reporting-summary-flat.pdf

Life sciences study design

All studies must disclose on these points even when the disclosure is negative.

Sample size	No sample-size calculation was performed. The manuscript reports the demonstration of an imaging method, but draws no biological conclusions, and does not examine or compare different biological conditions. This is not a life science study with comparative analyses of a certain sample size.
Data exclusions	No data was excluded from the analysis.
Replication	All attempts at replication were successful. All experiments were repeated three or more times with similar results.
Randomization	No randomization was performed. This is not a life science study with comparative analyses of biological situations.
Blinding	No blinding was performed. There is no comparison of different biological situations performed in this work.

Reporting for specific materials, systems and methods

We require information from authors about some types of materials, experimental systems and methods used in many studies. Here, indicate whether each material, system or method listed is relevant to your study. If you are not sure if a list item applies to your research, read the appropriate section before selecting a response.

Materials & experimental systems

n/a	Involved in the study	
☐	☒	Antibodies
☐	☒	Eukaryotic cell lines
☒	☐	Palaeontology and archaeology
☐	☒	Animals and other organisms
☒	☐	Human research participants
☒	☐	Clinical data
☒	☐	Dual use research of concern

Methods

n/a	Involved in the study	
☒	☐	ChIP-seq
☒	☐	Flow cytometry
☒	☐	MRI-based neuroimaging

Antibodies

Antibodies used	Mic60 (10179-1-AP, Proteintech, Rosemont, IL, USA, dilution 1:100); double-stranded DNA (ab27156, Abcam, dilution 1:3000). These primary antibodies were detected using Fab fragments coupled to Alexa Fluor 647 (A21246, Thermo Fisher, dilution 1:500) or secondary antibodies coupled to Cy5.5 (715-005-150, Jackson Immunoresearch, custom labeled, dilution 1:200). Beta-II spectrin antibody (612563, BD Biosciences, 1:200 dilution) detected by anti-mouse AlexaFluor 647 Fab Fragment (A21237, Thermo Fisher, 1:500 dilution).
Validation	Commercially available antibodies were applied to create specimens for testing the described microscopy technique. Validations were performed by the respective manufacturers. Mic60: This antibody was successfully used for more than 40 publications (according to the manufacturer). Antibody was e.g. knockout-validated by Western blotting and used for immunolabeling of mitochondria in: Stephan et al., 2020, EMBO J. dsDNA: This antibody was successfully used for more than 80 publications (according to the manufacturer). Antibody was e.g. used to label mitochondrial nucleoids in: Brueser et al., 2021, Cell Reports. Beta-II Spectrin: This antibody is routinely tested by the Western blot analysis (according to the manufacturer). This antibody has also been successfully used in multiple previous publications e.g. Xu et al.; Science, 2013, Zhong et al.; eLife, 2014; D'Este et al., Sci. Rep., 2016; D'Este et al., PNAS, 2017.

Eukaryotic cell lines

Policy information about cell lines

Cell line source(s)	COS-7 cells (ATTC); U-2 OS cells (ATCC); gene edited U-2 OS cells which express a SNAP-tagged version of the nucleoporin Nup107 (CLS Cell Lines Service, U-2OS-ZFN-SNAP-Nup107 clone #294); Nup96 (CLS Cell Lines Service, U-2OS-CRISPR-NUP96-SNAP clone #33).

Authentication	Cell lines were not further authenticated.
Mycoplasma contamination	Cells were tested negative for mycoplasma contamination.
Commonly misidentified lines (See ICLAC register)	No commonly misidentified cell lines were used.

Animals and other organisms

Policy information about studies involving animals; ARRIVE guidelines recommended for reporting animal research

Laboratory animals	Postnatal P0-P2 Wistar rats of either sex (0 to 2 days old)
Wild animals	No wild animals were involved.
Field-collected samples	No samples collected from the field were used.
Ethics oversight	Procedures were performed in accordance with the Animal Welfare Act of the Federal Republic of Germany (Tierschutzgesetz der Bundesrepublik Deutschland, TierSchG) and the Animal Welfare Laboratory Animal Regulations (Tierschutzversuchsverordnung). According to the TierSchG and the Tierschutzversuchsverordnung no ethical approval from the ethics committee was required for the procedure of sacrificing rodents for subsequent extraction of tissues, as performed in this study. The procedure for sacrificing P0-P2 rats performed in this study was supervised by animal welfare officers of the Max Planck Institute for Medical Research (MPImF) and conducted and documented according to the guidelines of the TierSchG (permit number assigned by the MPImF: MPI/T-35/18).

Note that full information on the approval of the study protocol must also be provided in the manuscript.
