# Supplementary material for: Optimal precision and accuracy in 4Pi-STORM using dynamic spline PSF models
Source: Nat Methods. 2022 May 16;19(5):603–12. doi: 10.1038/s41592-022-01465-8 (PMC9119851; doi:10.1038/s41592-022-01465-8)
Supplement: Supplementary file 3 — Supplementary software and data package. [file 41592_2022_1465_MOESM3_ESM.zip › supplementary_data_and_software/code/gpufit/Gpufit_1.2.0_Manual.pdf]

Gpufit Documentation

Release 1.2.0

Gpufit

Oct 13, 2021

CONTENTS

1 Introduction **1**
- 1.1 How to cite Gpufit . . . 2
- 1.2 Hardware requirements . . . 2
- 1.3 Software requirements . . . 2

2 Installation and Testing **3**
- 2.1 Gpufit binary distribution . . . 3
- 2.2 Building from source code . . . 3
  - 2.2.1 Prerequisites . . . 4
  - 2.2.2 Source code availability . . . 4
  - 2.2.3 Compiler configuration via CMake . . . 4
  - 2.2.4 Using the CMake Graphical User Interface . . . 5
  - 2.2.5 Running CMake from the command line . . . 5
  - 2.2.6 Common issues encountered during CMake configuration . . . 5
  - 2.2.7 Compiling Gpufit on Windows . . . 6
  - 2.2.8 Compiling Gpufit on Linux . . . 6
  - 2.2.9 MacOS . . . 7
- 2.3 Using cuBLAS . . . 7
- 2.4 Running the performance test . . . 7

3 Gpufit API description **9**
- 3.1 C Interface . . . 9
  - 3.1.1 gpufit() . . . 9
  - 3.1.2 gpufit_constrained() . . . 13
  - 3.1.3 gpufit_cuda_interface() . . . 14
  - 3.1.4 gpufit_constrained_cuda_interface() . . . 15
  - 3.1.5 gpufit_portable_interface() . . . 16
  - 3.1.6 gpufit_constrained_portable_interface() . . . 17
  - 3.1.7 gpufit_get_last_error() . . . 18
  - 3.1.8 gpufit_cuda_available() . . . 18
  - 3.1.9 gpufit_get_cuda_version() . . . 18

4 Fit Model functions **19**
- 4.1 Note: Handling of independent variables . . . 19
- 4.2 Linear regression . . . 19
- 4.3 1D Gaussian function . . . 20
- 4.4 2D Gaussian function (cylindrical symmetry) . . . 20
- 4.5 2D Gaussian function (elliptical) . . . 21
- 4.6 2D Gaussian function (elliptical, rotated) . . . 21
- 4.7 2D Cauchy function (elliptical) . . . 22
- 4.8 1D Spline function . . . 22
- 4.9 2D Spline function . . . 23
- 4.10 3D Spline function . . . 23
- 4.11 3D Multichannel Spline function . . . 24

4.12 3D Multichannel Spline function with variable phase . . . 24

5 Estimator functions **25**
5.1 Least squares estimator . . . 25
5.2 Maximum likelihood estimator for data subject to Poisson statistics . . . 25

6 Examples in C++ **26**
6.1 Simple example (minimal call to `gpufit()`) . . . 26
6.2 Example of 2D Gaussian fits . . . 27
6.2.1 Output statistics . . . 30
6.3 Linear Regression Example . . . 31

7 Customization **33**
7.1 Add a new fit model function . . . 33
7.2 Add a new fit estimator . . . 34
7.3 Future releases . . . 36

8 External bindings **37**
8.1 Optional parameters with default values . . . 37
8.2 Python . . . 37
8.2.1 Installation . . . 38
8.2.2 Python Interface . . . 38
8.2.3 Python Examples . . . 40
8.3 Matlab . . . 42
8.3.1 Matlab Interface . . . 42
8.3.2 Matlab Examples . . . 44
8.4 Java . . . 45
8.4.1 Installation . . . 46
8.4.2 Java Interface . . . 46
8.4.3 Java Example . . . 48

9 Appendix **51**
9.1 Levenberg-Marquardt algorithm . . . 51
9.2 Performance comparison to other GPU benchmarks . . . 51

10 Gpufit software license **54**

CHAPTER

ONE

INTRODUCTION

Gpufit is a GPU-accelerated CUDA implementation of the Levenberg-Marquardt algorithm. It was developed to meet the need for a high performance, general- purpose nonlinear curve fitting software library which is publicly available and open source.

Optimization algorithms are ubiquitous tools employed in many field of science and technology. One such algorithm for numerical, non-linear optimization is the Levenberg-Marquardt algorithm (LMA). The LMA combines elements of the method of steepest descent and Newton's method, and has become a standard algorithm for least-squares fitting. Box constraints on parameter values can be added as suitable projections during the optimization steps.

Although the LMA is, in itself, an efficient optimization algorithm, applications requiring many iterations of this procedure may encounter limitations due to the sheer number of calculations involved. The time required for the convergence of a fit, or a set of fits, can determine an application's feasibility, e.g. in the context of real-time data processing and feedback systems. Alternatively, in the case of very large datasets, the time required to solve a particular optimization problem may prove impractical.

In recent years, advanced graphics processing units (GPUs) and the development of general purpose GPU programming have enabled fast and parallelized computing by shifting calculations from the CPU to the GPU. The large number of independent computing units available on a modern GPU enables the rapid execution of many instructions in parallel, with an overall computation power far exceeding that of a CPU. Languages such as CUDA C and OpenCL allow GPU- based programs to be developed in a manner similar to conventional software, but with an inherently parallelized structure. These developments have led to the creation of new GPU-accelerated tools, such as the Gpufit.

Gpufit supports cubic spline functions that can be used to approximate arbitrary (smooth) fit model functions. In order to use them a spline representation of the model function must be provided (as an array of suitable spline coefficients). See Gpuspline on Github for details on how to compute these spline representations.

This manual describes how to install and build the Gpufit library and its external bindings. Furthermore it details how to extend Gpufit by adding custom model functions as well as custom fit estimator functions.

The documentation includes:

- Instructions for building and installing Gpufit
- A detailed description of the C interface
- A description of the built-in model functions
- A description of the built-in goodness-of-fit estimator functions
- A detailed description of the external bindings to Matlab and Python
- Usage examples for C, Matlab, and Python
- Instructions for adding custom model functions or custom estimator functions

The current version of the Gpufit library is 1.2.0 (see homepage). This manual was compiled on Oct 13, 2021.

1.1 How to cite Gpufit

Gpufit was created by Mark Bates, Adrian Przybylski, Björn Thiel, and Jan Keller-Findeisen at the Max Planck Institute for Biophysical Chemistry, in Göttingen, Germany.

The development and maintenance of open-source software projects, such as Gpufit, requires significant time and resources from the project team. If you use Gpufit in your research, **please cite our publication**. A paper describing the Gpufit software was published in the journal Scientific Reports, and is available from the Scientific Reports website (open-access), [here](https://www.nature.com/articles/s41598-017-15313-9).

The citation for the Gpufit paper is as follows:

```
Gpufit: An open-source toolkit for GPU-accelerated curve fitting
Adrian Przybylski, Björn Thiel, Jan Keller-Findeisen, Bernd Stock, and Mark Bates
Scientific Reports, vol. 7, 15722 (2017); doi: https://doi.org/10.1038/s41598-017-15313-9
```

1.2 Hardware requirements

Because the fit algorithm is implemented in CUDA C, a CUDA-compatible graphics card is required to run Gpufit. The minimum supported compute capability is 2.0. More advanced GPU hardware will result in higher fitting performance.

1.3 Software requirements

In addition to a compatible GPU, the graphics card driver installed on the host computer must be compatible with the version of the CUDA toolkit which was used to compile Gpufit. This may present an issue for older graphics cards or for computers running outdated graphics drivers.

At the time of its initial release in 2017, Gpufit was compiled with CUDA toolkit version 8.0. Therefore, the Nvidia graphics driver installed on the host PC must be at least version 367.48 (released July 2016) in order to be compatible with the binary files generated in this build.

When compatibility issues arise, there are two possible solutions. The best option is to update the graphics driver to a version which is compatible with the CUDA toolkit used to build Gpufit. The second option is to re-compile Gpufit from source code, using an earlier version of the CUDA toolkit which is compatible with the graphics driver in question. However, this solution is likely to result in slower performance of the Gpufit code, since older versions of the CUDA toolkit are not as efficient.

Note that all CUDA-supported graphics cards should be compatible with CUDA toolkit version 6.5. This is the last version of CUDA which supported GPUs with compute capability 1.x. In other words, an updated Nvidia graphics driver should be available for all CUDA-enabled GPUs which is compatible with toolkit version 6.5.

If you are unsure if your graphics card is CUDA-compatible, a lists of CUDA supported GPUs can be found here.

CHAPTER

TWO

INSTALLATION AND TESTING

The Gpufit library can be used in several ways. When using a pre-compiled binary version of Gpufit, the Gpufit functions may be accessed directly via a dynamic linked library (e.g. Gpufit.dll) or via the external bindings to Gpufit (e.g. the Matlab or Python bindings). For more information on the Gpufit interface, see *Gpufit API description* (page 9), or for details of the external bindings see *External bindings* (page 37).

This section describes how to compile Gpufit, including generating its external bindings, from source code. Building from source is necessary when a fit model function is added or changed, or if a new fit estimator is required. Building the library may also be useful for compiling the code using a specific version of the CUDA toolkit, or for a particular CUDA compute capability.

2.1 Gpufit binary distribution

A binary distribution of the Gpufit library is available for **Windows**. Use of this distribution requires only a CUDA-capable graphics card, and an updated Nvidia graphics driver. The binary package contains:

- The Gpufit SDK, which consists of the 32-bit and 64-bit DLL files, and the Gpufit header file which contains the function definitions. The Gpufit SDK is intended to be used when calling Gpufit from an external application written in e.g. C code.
- The performance test application, which serves to test that Gpufit is correctly installed, and to check the performance of the CPU and GPU hardware.
- Matlab 32 bit and 64 bit bindings, with Matlab examples.
- Python version 2.x and version 3.x bindings (compiled as wheel files) and Python examples.
- Java 8 32 bit and 64 bit bindings, with Java examples.
- This manual in PDF format.

To re-build the binary distribution, see the instructions located in package/README.md.

2.2 Building from source code

This section describes how to build Gpufit from source code. Note that as of the initial release of Gpufit, the source code has been tested only with the Microsoft Visual Studio compiler.

2.2.1 Prerequisites

The following tools are required in order to build Gpufit from source.

Required

- CMake 3.11 or later
- A C/C++ Compiler
  - Linux: GCC 4 (tested with 4-6)
  - Windows: Visual Studio 2013 (tested with 2013 - 2019)
- CUDA Toolkit 6.5 or later (tested with 6.5-11.4)[1]

Optional

- Boost 1.58 or later (required if you want to build the tests)
- MATLAB if building the MATLAB bindings (minimum version Matlab 2012a)
- Python if building the Python bindings (Python version 2.x or 3.x). Note that the "wheel" package is required when building the Python binding.
- Java if building the Java bindings (minimum Java JDK version 8)
- PDF Latex installation (like Miktex) if converting the documentation from Latex to PDF.

2.2.2 Source code availability

The source code is available in an open repository hosted at Github, at the following URL.

```
https://github.com/gpufit/Gpufit.git
```

To obtain the code, Git may be used to clone the repository, or a current snapshot may be downloaded directly from Github as Gpufit-master.zip.

2.2.3 Compiler configuration via CMake

CMake is an open-source tool designed to build, test, and package software. It is used to control the software compilation process using compiler independent configuration files, and generate native makefiles and workspaces that can be used in the compiler environment. In this section we provide a simple example of how to use CMake in order to generate the input files for the compiler (e.g. the Visual Studio solution file), which can then be used to compile Gpufit.

First, identify the directory which contains the Gpufit source code (for example, on a Windows computer the Gpufit source code may be stored in *C:\Sources\Gpufit*). Next, create a build directory outside the source code source directory (e.g. *C:\Sources\Gpufit-build-64*). Finally, run cmake to configure and generate the compiler input files.

[1] Note that it is recommended to use the newest available stable release of the CUDA Toolkit which is compatible with the compiler (e.g. Visual Studio 2015 is required in order to use CUDA Toolkit 8.0). Some older graphics cards may only be supported by CUDA Toolkit version 6.5 or earlier. Also, when using CUDA Toolkit version 6.5, please use the version with support for GTX9xx GPUs, available here.

2.2.4 Using the CMake Graphical User Interface

There is a graphical user interface available for CMake, which simplifies the configuration and generation steps. For further details, see Running CMake. The following steps outline how to use the basic features of the CMake GUI.

First, select the source code directory (the top level directory where the Gpufit source code is located), and the build directory (where the binaries will be built). For this example, the source directory might be *C:\Sources\Gpufit*, and the build directory might be *C:\Sources\Gpufit-build-64*.

Next, click the "Configure" button, and select the desired compiler from the drop down list (e.g. Visual Studio 12 2013). Under *Optional platform for Generator*, select the desired architecture (e.g. *x64* to compile 64-bit binaries).

Once configuration is complete, CMake will have automatically found the Matlab installation, and the installation directories will be listed in the *NAME* and *VALUE* columns. If the Matlab installation was not found, the entries in the *VALUE* column can be manually edited.

Next, click on *Generate* to generate the Visual Studio solution files, which will be used to build the Gpufit package.

2.2.5 Running CMake from the command line

The following commands, executed from the command prompt, assume that the cmake executable (e.g. *C:\Program Files\CMake\bin\cmake.exe*) is automatically found via the PATH environment variable (if not, the full path to cmake.exe must be specified). This example also assumes that the source and build directories have been set up as specified above.

```
cd C:\Sources\Gpufit-build-64
cmake -G "Visual Studio 12 2013 Win64" C:\Sources\Gpufit
```

Note that in this example the *-G* flag has been used to specify the 64-bit version of the Visual Studio 12 compiler. This flag should be changed depending on the compiler used, and the desired architecture (e.g. 32- or 64-bit). Further details of the CMake command line arguments can be found here.

There is also a graphical user interface available for CMake, which simplifies the configuration and generation steps. For further details, see Running CMake.

2.2.6 Common issues encountered during CMake configuration

It's strongly recommended to use the latest available CMake version. Especially the find CUDA, Matlab, Python capabilities of CMake get updated often.

Boost NOT found - skipping tests!

If you want to build the tests and Boost is not found automatically, set the CMake variable BOOST_ROOT to the corresponding directory, and configure again.

JAVA JNI NOT found - skipping Gpufit Java binding!

If you want to build the Java binding and CMake cannot find Java, you can set the CMake variable JAVA_HOME to specify a Java installation explicitely.

Specify CUDA_ARCHITECTURES set

If you need a specific CUDA architecture, set CUDA_ARCHITECTURES according to CUDA_SELECT_NVCC_ARCH_FLAGS.

CMake finds last installed CUDA toolkit version by default

If there are multiple CUDA toolkits installed on the computer, CMake 3.7.1 seems to find by default the lowest installed version. In this case set the desired CUDA version manually (e.g. by editing the CUDA_TOOLKIT_ROOT_DIR variable in CMake).

Specify CUDA version to use

Set CUDA_BIN_PATH before running CMake or CUDA_TOOLKIT_ROOT_DIR after first CMAKE configuration to the installation folder of the desired CUDA version.

Required CUDA version

When using Microsoft Visual Studio 2015, the minimum required CUDA Toolkit version is 8.0.

No suitable Matlab and/or MX_Library version found - skipping Gpufit Matlab binding!

CMake might not be able to locate Matlab, in which case this message will be shown. Try to set the Matlab_ROOT_DIR variable manually (for example "C:/Program Files/MATLAB/R2020b" or "/usr/local/Matlab/2020b" on Linux) and run CMake again.

Python launcher

Set Python_WORKING_DIRECTORY to a valid directory, it will be added to the Python path.

Matlab launcher

Set Matlab_WORKING_DIRECTORY to a valid directory, it will be added to the Matlab path.

Documentation build issues

Note that the several Python packages are required to run the "documentation_create_latex.bat" script on Windows systems. Please ensure that the "sphinx" and "sphinx_rtd_style" packages are installed in your Python distribution.

PDFLATEX not found

When using Miktex, if the PDFLATEX package is not automatically found, the path to pdflatex.exe can be specified to CMake with the MIKTEX_BINARY_PATH variable (available under Advanced options).

2.2.7 Compiling Gpufit on Windows

After configuring and generating the solution files using CMake, go to the desired build directory and open Gpufit.sln using Visual Studio. Select the "Debug" or "Release" build options, as appropriate. Select the build target "ALL_BUILD", and build this target. If the build process completes without errors, the Gpufit binary files will be created in the corresponding "Debug" or "Release" folders in the build directory.

The unit tests can be executed by building the target "RUN_TESTS" or by starting the created executables in the output directory from the command line.

2.2.8 Compiling Gpufit on Linux

A successful build has been verified on Ubuntu 18.04 LTS with gcc 5.5 and CUDA 9.1 following the instructions on the NVidia website. CMake needs to be at least version 3.11. To perform the tests, a development version of Boost should be installed (e.g. *libboost-all-dev*).

The following commands were executed.

```
git clone https://github.com/gpufit/Gpufit.git Gpufit
mkdir Gpufit-build
cd Gpufit-build
cmake -DCMAKE_BUILD_TYPE=RELEASE ../Gpufit
make
```

In case, during make there is an error "unsupported GNU version! gcc versions later than X are not supported", it means that CUDA needs an older version of gcc. Provided that such a version is installed on the system you can choose it with the -DCMAKE_C_COMPILER option to cmake. For example, for CUDA 9 one should add -DCMAKE_C_COMPILER=gcc-5 in the call to cmake.

The tests can be run for example by "make test". Run the performance comparison with

```
./Gpufit_Cpufit_performance_comparison
```

To install the Python package

```
cd pyGpufit/dist
pip install pyGpufit-X.Y.Z-py2.py3-none-any.whl
```

To run the Matlab package you need to tell Matlab to use a newer version of the C++ standard library

```
export LD_PRELOAD=/usr/lib/x86_64-linux-gnu/libstdc++.so.6
matlab
```

Then in Matlab add the matlab output directory to the path and execute some examples.

```
addpath('XX/Gpufit-build/matlab');
cd('XX/Gpufit/Gpufit/matlab/examples');
gauss2d();
```

2.2.9 MacOS

Gpufit has not yet been officially tested on a computer running MacOS with a CUDA capable graphics card. However, satisfying the *Prerequisites* (page 4) and using CMake, we estimate that the library should build in principle and one should also be able to run the examples on MacOS.

2.3 Using cuBLAS

Optionally, Gpufit may be configured to make use of the cuBLAS library. This library is used for solving the equation system during the fit process.

Several benefits are obtained by using cuBLAS:

- Moderately faster fitting performance.
- Improved numerical stability.
- The number of fit model parameters may be as high as the number of threads per GPU thread block (1024). Without cuBLAS, the maximum number of model parameters is limited to 31.

To include cuBLAS functionality in Gpufit, the USE_CUBLAS flag may be set during the CMAKE configuration step. Note that the Gpufit cuBLAS feature is only available for 64-bit architectures, and for code built with CUDA toolkit version >= 7.0. Finally, it is important to note that cuBLAS is not statically linked in the Gpufit DLL, but rather it is dynamically linked. Hence, when distributing applications, the cuBLAS DLL (e.g. cublas64_80.dll for CUDA toolkit version 8) must be packaged along with Gpufit.dll.

2.4 Running the performance test

The Gpufit performance test is a program which verifies the correct function of Gpufit, and tests the fitting speed in comparison with the same algorithm executed on the CPU.

If Gpufit was built from source, running the build target GPUFIT_CPUFIT_Performance_Comparison will run the test, which executes the fitting process multiple times, varying the number of fits per function call. The execution time is measured in each case and the relative speed improvement between the GPU and the CPU is calculated. A successful run of the performance test also indicates also that Gpufit is functioning correctly.

The performance comparison is also included in the Gpufit binary distribution as a console application. An example of the program's output is shown in Fig. 2.1.

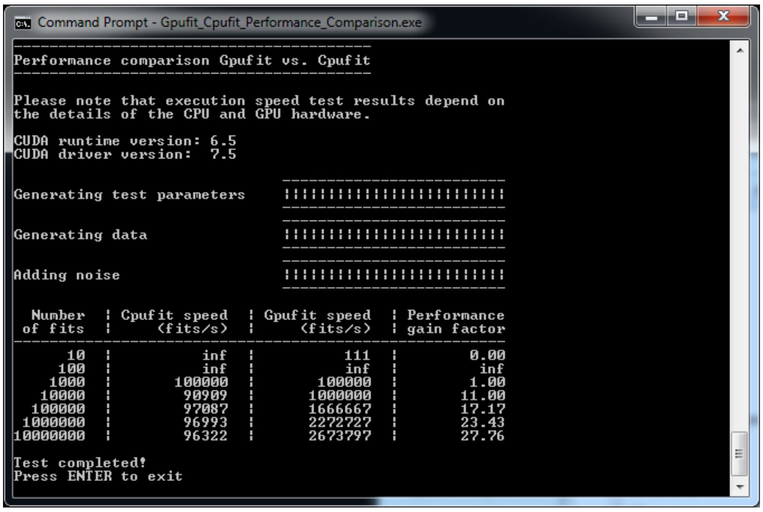

Fig. 2.1: Output of the Gpufit vs Cpufit performance comparison

CHAPTER

THREE

GPUFIT API DESCRIPTION

The Gpufit source code compiles to a dynamic-link library (DLL), providing a C interface. In the sections below, the C interface and its arguments are described in detail.

3.1 C Interface

The C interface is defined in the Gpufit header file: gpufit.h.

3.1.1 gpufit()

This is the main fit function. A single call to the `gpufit()` function executes a block of N fits. The inputs to `gpufit()` are scalars and pointers to arrays, and the outputs are also array pointers.

The inputs to the `gpufit()` function are:

- the number of fits (N),
- the number of data points per fit (each fit has equal size),
- the fit data,
- an array of weight values that are used to weight the individual data points in the fit (optional),
- an ID number which specifies the fit model function,
- an array of initial parameters for the model functions,
- a tolerance value which determines when the fit has converged,
- the maximum number of iterations per fit,
- an array of flags which allow one or more fit parameters to be held constant,
- an ID number which specifies the fit estimator (e.g. least squares, etc.),
- the size of the user info data,
- the user info data, which may have multiple uses, for example to pass additional parameters to the fit functions, or to include independent variables (e.g. X values) with the fit data.

The outputs of `gpufit()` are:

- the best fit model parameters for each fit,
- an array of flags indicating, for example, whether each fit converged,
- the final value of χ^2 for each fit,
- the number of iterations needed for each fit to converge.

The `gpufit()` function call is defined below.

```
int gpufit
(
    size_t n_fits,
    size_t n_points,
    float * data,
    float * weights,
    int model_id,
    float * initial_parameters,
    float tolerance,
    int max_n_iterations,
    int * parameters_to_fit,
    int estimator_id,
    size_t user_info_size,
    char * user_info,
    float * output_parameters,
    int * output_states,
    float * output_chi_squares,
    int * output_n_iterations
) ;
```

Description of input parameters

n_fits Number of fits to be performed

type size_t

n_points Number of data points per fit

Gpufit is designed such that each fit must have the same number of data points per fit.

type size_t

data Pointer to data values

A pointer to the data values. The data must be passed in as a 1D array of floating point values, with the data for each fit concatenated one after another. In the case of multi-dimensional data, the data must be flattened to a 1D array. The number of elements in the array is equal to the product n_fits * n_points.

type float *

length n_points * n_fits

weights Pointer to weights

The weights array includes unique weighting values for each fit. It is used only by the least squares estimator (LSE). The size of the weights array and its organization is identical to that for the data array. For statistical weighting, this parameter should be set equal to the inverse of the variance of the data (i.e. weights = 1.0 / variance). The weights array is an optional input.

type float *

length n_points * n_fits

special Use a NULL pointer to indicate that no weights are provided. In this case all data values will be weighted equally.

model_id Model ID

Determines the model which is used for all fits in this call. See *Fit Model functions* (page 19) for more details.

As defined in constants.h:

0 GAUSS_1D

1 GAUSS_2D

2 GAUSS_2D_ELLIPTIC

3 GAUSS_2D_ROTATED

4 CAUCHY_2D_ELLIPTIC

5 LINEAR_1D

type int

initial_parameters Pointer to initial parameter values

A 1D array containing the initial model parameter values for each fit. If the number of parameters of the fit model is defined by *n_parameters*, then the size of this array is *n_fits * n_parameters*.

The parameter values for each fit are concatenated one after another. If there are *M* parameters per fit, the parameters array is organized as follows: [(parameter 1), (parameter 2), . . . , (parameter M), (parameter 1), (parameter 2), . . . , (parameter M), . . .].

type float *

length n_fits * n_parameters

tolerance Fit tolerance threshold

The fit tolerance determines when the fit has converged. After each fit iteration, the change in the absolute value of χ^2 is calculated. The fit has converged when one of two conditions are met. First, if the change in the absolute value of χ^2 is less than the tolerance value, the fit has converged. Alternatively, if the change in χ^2 is less than the product of tolerance and the absolute value of χ^2 [tolerance * abs(χ^2)], then the fit has converged.

Setting a lower value for the tolerance results in more precise values for the fit parameters, but requires more fit iterations to reach convergence.

A typical value for the tolerance settings is between 1.0E-3 and 1.0E-6.

type float

max_n_iterations Maximum number of iterations

The maximum number of fit iterations permitted. If the fit has not converged after this number of iterations, the fit returns with a status value indicating that the maximum number of iterations was reached.

type int

parameters_to_fit Pointer to array indicating which model parameters should be held constant during the fit

This is an array of ones or zeros, with a length equal to the number of parameters of the fit model function. Each entry in the array is a flag which determines whether or not the corresponding model parameter will be held constant during the fit. To allow a parameter to vary during the fit, set the entry in *parameters_to_fit* equal to one. To hold the value constant, set the entry to zero.

An array of ones, e.g. [1,1,1,1,1,. . .] will allow all parameters to vary during the fit.

type int *

length n_parameters

estimator_id Estimator ID

Determines the fit estimator which is used. See *Estimator functions* (page 25) for more details.

As defined in constants.h:

0 LSE

1 MLE

type int

user_info_size Size of user information data

Size of the user information data array, in bytes.

type size_t

user_info Pointer to user information data

This parameter is intended to provide flexibility to the Gpufit interface. The user information data is a generic block of memory which is passed in to the `gpufit()` function, and which is accessible in shared GPU memory by the fit model functions and the estimator functions. Possible uses for the user information data are to pass in values for independent variables (e.g. X values) or to supply additional data to the fit model function or estimator. The documentation of the fit model function or estimator must specify the composition of the user info data. For a coded example which makes use of the user information data, see *Linear Regression Example* (page 31). The user information data is an optional parameter - if no user information is required this parameter may be set to NULL.

type char *

length user_info_size

special Use a NULL pointer to indicate that no user information is available. The interpretation of the user info depends completely on the used fit model function or estimator.

Description of output parameters

output_parameters Pointer to array of best-fit model parameters

For each fit, this array contains the best-fit model parameters. The array is organized identically to the input parameters array.

type float *

length n_fits * n_parameters

output_states Pointer to array of fit result state IDs

For each fit the result of the fit is indicated by a state ID. The state ID codes are defined below. A state ID of 0 indicates that the fit converged successfully.

As defined in constants.h:

0 The fit converged, tolerance is satisfied, the maximum number of iterations is not exceeded

1 Maximum number of iterations exceeded

2 During the Gauss-Jordan elimination the Hessian matrix is indicated as singular

3 Non-positive curve values have been detected while using MLE (MLE requires only positive curve values)

4 State not read from GPU Memory

type int *

length n_fits

output_chi_squares Pointer to array of χ^2 values

For each fit, this array contains the final χ^2 value, as returned by the estimator function (see *Estimator functions* (page 25)).

type float *

length n_fits

output_n_iterations Pointer to array of iteration counts

For each fit, this array contains the number of fit iterations which were performed.

type int *

length n_fits

return value Status code

The return value of the function call indicates whether an error occurred. As defined in constants.h.

0 No error

-1 Error

3.1.2 gpufit_constrained()

This is very similar to the `gpufit()` function but with the additional possibility to add box constraints on the allowed parameter ranges.

The `gpufit_constrained()` function call is defined below.

```
int gpufit_constrained
(
    size_t n_fits,
    size_t n_points,
    float * data,
    float * weights,
    int model_id,
    float * initial_parameters,
    float * constraints,
    int * constraint_types,
    float tolerance,
    int max_n_iterations,
    int * parameters_to_fit,
    int estimator_id,
    size_t user_info_size,
    char * user_info,
    float * output_parameters,
    int * output_states,
    float * output_chi_squares,
    int * output_n_iterations
) ;
```

In order to not repeat the same information all input and output parameters in `gpufit_constrained()` that also exist in `gpufit()` have exactly the same definition and interpretation. Below only the additional input parameter regarding the constraints are explained.

Description of constraints input parameters

constraints Pointer to model parameter constraint intervals

A 1D array containing the model parameter constraint lower and upper bounds for all parameters (including fixed parameters) and for all fits. Order is lower, upper bound first, then parameters, then number of fits.

type float *

length n_fits * n_parameters * 2

constraint_types Pointer to constraint types for each parameter

A 1D array containing the constraint types for each parameter (including fixed parameters). The constraint type is defined by an *int* with 0 - no constraint, 1 - only constrain lower bound, 2 - only constrain upper bound, 3 - constrain both lower and upper bounds.

type int *

length n_parameters

3.1.3 gpufit_cuda_interface()

This function performs the fitting without transferring the input and output data between CPU and GPU memory. The allocation of GPU memory for input and output data is skipped, as well. The structures of input and output arrays are equal to the main interface function `gpufit()`. There are no separate arrays for initial and best-fit parameter values. The argument `gpu_fit_parameters` points to initial parameter values at start of the routine and to best-fit parameter values at the end.

```
int gpufit_cuda_interface
(
    size_t n_fits,
    size_t n_points,
    float * gpu_data,
    float * gpu_weights,
    int model_id,
    float tolerance,
    int max_n_iterations,
    int * parameters_to_fit,
    int estimator_id,
    size_t user_info_size,
    char * gpu_user_info,
    float * gpu_fit_parameters,
    int * gpu_output_states,
    float * gpu_output_chi_squares,
    int * gpu_output_n_iterations
) ;
```

Description of input parameters

n_fits Number of fits to be performed

type size_t

n_points Number of data points per fit

type size_t

gpu_data Pointer to data values stored on GPU

type float *

length n_points * n_fits

gpu_weights Pointer to weights stored on GPU

type float *

length n_points * n_fits

special Use a NULL pointer to indicate that no weights are provided. In this case all data values will be weighted equally.

model_id Model ID

type int

tolerance Fit tolerance threshold

type float

max_n_iterations Maximum number of iterations

type int

parameters_to_fit Pointer to array indicating which model parameters should be held constant during the fit

type int *

length n_parameters

estimator_id Estimator ID

type int

user_info_size Size of user information data

type size_t

gpu_user_info Pointer to user information data stored on GPU

type char *

length user_info_size

special Use a NULL pointer to indicate that no user information is available.

Description of input/output parameters

gpu_fit_parameters Pointer to array of model parameters stored on GPU

input: initial parameter values

output: best-fit parameter values

type float *

length n_fits * n_parameters

Description of output parameters

gpu_output_states Pointer to array of fit result state IDs stored on GPU

type int *

length n_fits

gpu_output_chi_squares Pointer to array of χ^2 values stored on GPU

type float *

length n_fits

gpu_output_n_iterations Pointer to array of iteration counts stored on GPU

type int *

length n_fits

return value Status code

0 No error

-1 Error

3.1.4 gpufit_constrained_cuda_interface()

This function is very similar to the `gpufit_cuda_interface()` function but with the additional possibility to add box constraints on the allowed parameter ranges.

```
int gpufit_constrained_cuda_interface
(
    size_t n_fits,
    size_t n_points,
    float * gpu_data,
    float * gpu_weights,
    int model_id,
    float tolerance,
    int max_n_iterations,
```

(continues on next page)

(continued from previous page)

```
    int * parameters_to_fit,
    float * gpu_constraints,
    int * constraint_types,
    int estimator_id,
    size_t user_info_size,
    char * gpu_user_info,
    float * gpu_fit_parameters,
    int * gpu_output_states,
    float * gpu_output_chi_squares,
    int * gpu_output_n_iterations
) ;
```

In order to not repeat the same information all input and output parameters in `gpufit_constrained_cuda_interface()` that also exist in `gpufit_cuda_interface()` have exactly the same definition and interpretation. Below only the additional input parameter regarding the constraints are explained.

Description of constraint input parameters

gpu_constraints Pointer to model parameter constraint intervals stored on the GPU

A 1D array containing the model parameter constraint lower and upper bounds for all parameters (including fixed parameters) and for all fits. Order is lower, upper bound first, then parameters, then number of fits.

type float *

length n_fits * n_parameters * 2

constraint_types Pointer to constraint types for each parameter

A 1D array containing the constraint types for each parameter (including fixed parameters). The constraint type is defined by an *int* with 0 - no constraint, 1 - only constrain lower bound, 2 - only constrain upper bound, 3 - constrain both lower and upper bounds.

type int *

length n_parameters

3.1.5 gpufit_portable_interface()

This function is a simple wrapper around the `gpufit()` function, providing an alternative means of passing the function parameters.

```
int gpufit_portable_interface(int argc, void *argv[]);
```

Description of parameters

argc The length of the argv pointer array

argv Array of pointers to *gpufit* parameters, as defined above. For reference, the type of each element of the *argv* array is listed below.

argv[0] Number of fits

type size_t *

argv[1] Number of points per fit

type size_t *

argv[2] Fit data

type float *

argv[3] Fit weights

type float *

argv[4] Fit model ID

type int *

argv[5] Initial parameters

type float *

argv[6] Fit tolerance

type float *

argv[7] Maximum number of iterations

type int *

argv[8] Parameters to fit

type int *

argv[9] Fit estimator ID

type int *

argv[10] User info size

type size_t *

argv[11] User info data

type char *

argv[12] Output parameters

type float *

argv[13] Output states

type int *

argv[14] Output χ^2 values

type float *

argv[15] Output number of iterations

type int *

return value This function simply returns the `gpufit()` return status code.

3.1.6 gpufit_constrained_portable_interface()

This function is a simple wrapper around the `gpufit_constrained()` function, providing an alternative means of passing the function parameters.

```
int gpufit_constrained_portable_interface(int argc, void *argv[]);
```

Description of parameters

argc The length of the argv pointer array

argv Array of pointers to *gpufit_constrained* parameters, as defined above.

return value This function simply returns the `gpufit()` return status code.

3.1.7 gpufit_get_last_error()

A function that returns a string representation of the last error.

```
char const * gpufit_get_last_error();
```

return value Error message corresponding to the most recent error, or an empty string if no error occurred.

'CUDA driver version is insufficient for CUDA runtime version' The graphics driver version installed on the computer is not supported by the CUDA Toolkit version which was used to build Gpufit.dll. Update the graphics driver or re-build Gpufit using a compatible CUDA Toolkit version.

'too many resources requested for launch' Exceeded number of available registers per thread block. Adding model functions to models.cuh can increase the number of registers per thread used by the kernel cuda_calc_curve_values(). If this error occurs, comment out unused models in function calculate_model() in file models.cuh.

3.1.8 gpufit_cuda_available()

A function that calls a simple CUDA function to check if CUDA is available.

```
int gpufit_cuda_available();
```

return value Returns 0 if CUDA is not available (no suitable device found, or driver version insufficient). Use the function *gpufit_get_last_error()* to check the error message. Returns 1 if CUDA is available.

3.1.9 gpufit_get_cuda_version()

A function that returns the CUDA runtime version in *runtime_version* and the installed CUDA driver version in *driver_version*.

```
int gpufit_get_cuda_version(int * runtime_version, int * driver_version);
```

runtime_version Pointer to the CUDA runtime version number. Format is Minor version times 10 plus Major version times 1000. (is 0 if the CUDA runtime version is incompatible with the installed CUDA driver version)

driver_version Pointer to the CUDA driver version number. Format is Minor version times 10 plus Major version times 1000. (is 0 if no CUDA enabled graphics card was detected)

return value Status code

The return value of the function call indicates whether an error occurred.

0 No error

-1 Error. Use the function *gpufit_get_last_error()* to check the error message.

CHAPTER

FOUR

FIT MODEL FUNCTIONS

This section describes the fit model functions which are included with the Gpufit library. The model IDs usable in the call of the Gpufit *C Interface* (page 9) are defined in constants.h. Note that additional model functions may be added as described in the documentation, see *Customization* (page 33).

4.1 Note: Handling of independent variables

We note that, in the current version of the Gpufit library, the indepenent variables (e.g. X values) corresponding to the fit data are not passed into the `gpufit()` function. The default behavior of the fit models functions is to assume the coordinates of the data values are uniformly spaced and monotonically increasing (see below).

However, the `user_info` parameter of the Gpufit interface allows arbitrary data to be passed to the model functions and estimators. Hence, this mechanism may be used to supply independent variables to the fit. In the current release, two model functions support this method: the linear regression model and the one-dimensional Gaussian model. This is described in more detail in the sections below, and an example is given in the Examples section of the documentation.

The *Linear regression* (page 19) and *1D Gaussian function* (page 20) models provide an option to pass in custom X coordinate values, using the user information parameter. In this case, the data type of the values must be single precision floating point. The user has two options for how to use this mechanism: one set of X values may be provided and used for all fits, or unique X values may be provided for all fits. The first option allows for faster calculations, since it requires fewer data transfer operations. If no X values are provided, the models assume that the data coordinates start with zero, as described above.

When calling Gpufit by its *C Interface* (page 9), the user information size parameter must be set to the product of the number of values in the user information array and the size of the data type in bytes. The number of the X coordinate values must be equal to the total number of data points, or the number of data points per fit.

4.2 Linear regression

A 1D linear function defined by two parameters (offset and slope). The model ID of this function is `LINEAR_1D`, and it is implemented in linear_1d.cuh.

Optional: The X coordinate of each data point may be specified via the user information data parameter of the Gpufit interface. The user information should then contain X coordinate values of type float in increasing order.

Default X coordinates If user information is not provided, the X coordinate of the first data value is assumed to be (0.0). In this case, for a fit size of M data points, the X coordinates of the data are set equal to the indices of the data array, starting from zero (i.e. $0, 1, 2, ..., M - 1$).

Identical X coordinate values for all fits If the number of values in the user information array is equal to the number of data points per fit, the same X coordinate values are used for all fits.

Unique X coordinate values for each fit If the number of values in the user information array is equal to the total number of data points, unique *X* coordinate values are used for each fit.

$$g(x, \vec{p}) = p_0 + p_1 x$$

x (independent variable) *X* coordinate

The *X* coordinate values may be specified in the user information data. For details, see the linear regression code example, *Linear Regression Example* (page 31).

p_0 offset

p_1 slope

4.3 1D Gaussian function

A 1D Gaussian function defined by four parameters. Its model ID is `GAUSS_1D` and it is implemented in gauss_1d.cuh. The user information data may be used to specify the X coordinate of each data point. Here, `p` is the vector of parameters (p0..p3) and the model function `g` exists for each *X* coordinate of the input data.

Optional: The *X* coordinate of each data point may be specified via the user information data parameter of the Gpufit interface.

Default X coordinates If user information is not provided, the *X* coordinate of the first data value is assumed to be (0.0). In this case, for a fit size of *M* data points, the *X* coordinates of the data are set equal to the indices of the data array, starting from zero (i.e. $0, 1, 2, ..., M - 1$).

Identical X coordinate values for all fits If the number of values in the user information array is equal to the number of data points per fit, the same *X* coordinate values are used for all fits.

Unique X coordinate values for each fit If the number of values in the user information array is equal to the total number of data points, unique *X* coordinate values are used for each fit.

$$g(x, \vec{p}) = p_0 e^{-(x-p_1)^2/\left(2p_2^2\right)} + p_3$$

x (independent variable) *X* coordinate

The X coordinate values may be specified in the user information data. For details on how to do this, see the linear regression code example, *Linear Regression Example* (page 31).

p_0 amplitude

p_1 center coordinate

p_2 width (standard deviation)

p_3 offset

4.4 2D Gaussian function (cylindrical symmetry)

A 2D Gaussian function defined by five parameters. Its model ID is `GAUSS_2D` and it is implemented in gauss_2d.cuh. Here, `p` is the vector of parameters (p0..p4) and the model function `g` exists for each x,y coordinate of the input data.

$$g(x, y, \vec{p}) = p_0 e^{-\left((x-p_1)^2+(y-p_2)^2\right)/\left(2p_3^2\right)} + p_4$$

x,y (independent variables) *X,Y* coordinates

No independent variables are passed to this model function. Hence, the *(X,Y)* coordinates of the first data value are assumed to be $(0.0, 0.0)$. The fit size is *M x M* data points (M*M=number of data points in the interface), the *(X,Y)* coordinates of the data are simply the corresponding 2D array indices of the data array, starting from zero.

p_0 amplitude

p_1 center coordinate x

p_2 center coordinate y

p_3 width (standard deviation; equal width in x and y dimensions)

p_4 offset

4.5 2D Gaussian function (elliptical)

A 2D elliptical Gaussian function defined by six parameters. Its model ID is `GAUSS_2D_ELLIPTIC` and it is implemented in gauss_2d_elliptic.cuh. Here, `p` is the vector of parameters (p0..p5) and the model function `g` exists for each x,y coordinate of the input data.

$$g(x, y, \vec{p}) = p_0 e^{-\frac{1}{2}\left(\frac{(x-p_1)^2}{p_3^2} + \frac{(y-p_2)^2}{p_4^2}\right)} + p_5$$

x,y (independent variables) *X,Y* coordinates

No independent variables are passed to this model function. Hence, the *(X,Y)* coordinates of the first data value are assumed to be $(0.0, 0.0)$. The fit size is *M x M* data points (M*M=number of data points in the interface), the *(X,Y)* coordinates of the data are simply the corresponding 2D array indices of the data array, starting from zero.

p_0 amplitude

p_1 center coordinate x

p_2 center coordinate y

p_3 width x (standard deviation)

p_4 width y (standard deviation)

p_5 offset

4.6 2D Gaussian function (elliptical, rotated)

A 2D elliptical Gaussian function whose principal axis may be rotated with respect to the X and Y coordinate axes, defined by seven parameters. Its model is `GAUSS_2D_ROTATED` and it is implemented in gauss_2d_rotated.cuh. Here, `p` is the vector of parameters (p0..p6) and the model function `g` exists for each x,y coordinate of the input data.

$$g(x, y, \vec{p}) = p_0 e^{-\frac{1}{2}\left(\frac{((x-p_1)\cos p_6-(y-p_2)\sin p_6)^2}{p_3^2} + \frac{((x-p_1)\sin p_6+(y-p_2)\cos p_6)^2}{p_4^2}\right)} + p_5$$

x,y (independent variables) *X,Y* coordinates

No independent variables are passed to this model function. Hence, the *(X,Y)* coordinates of the first data value are assumed to be $(0.0, 0.0)$. The fit size is *M x M* data points (M*M=number of data points in the interface), the *(X,Y)* coordinates of the data are simply the corresponding 2D array indices of the data array, starting from zero.

p_0 amplitude

p_1 center coordinate x

p_2 center coordinate y

p_3 width x (standard deviation)

p_4 width y (standard deviation)

p_5 offset

p_6 rotation angle [radians]

4.7 2D Cauchy function (elliptical)

A 2D elliptical Cauchy function defined by six parameters. Its model ID is `CAUCHY_2D_ELLIPTIC` and it is implemented in cauchy_2d_elliptic.cuh. Here, `p` is the vector of parameters (p0..p5) and the model function `g` exists for each x,y coordinate of the input data.

$$g(x,y,\vec{p}) = p_0 \frac{1}{\left(\frac{x-p_1}{p_3}\right)^2+1} \frac{1}{\left(\frac{y-p_2}{p_4}\right)^2+1} + p_5$$

x,y (independent variables) *X,Y* coordinates

No independent variables are passed to this model function. Hence, the *(X,Y)* coordinates of the first data value are assumed to be $(0.0, 0.0)$. The fit size is *M x M* data points (M*M=number of data points in the interface), the *(X,Y)* coordinates of the data are simply the corresponding 2D array indices of the data array, starting from zero.

p_0 amplitude

p_1 center coordinate x

p_2 center coordinate y

p_3 width x (standard deviation)

p_4 width y (standard deviation)

p_5 offset

4.8 1D Spline function

A 1D cubic spline function defined by 3 parameters and a set of cubic spline coefficients. See Gpuspline on Github for details on how to compute the set of cubic spline coefficients from a data set so that it can be used here. The model ID is `SPLINE_1D` and it is implemented in spline_1d.cuh. Here, `p` is the vector of parameters (p0..p2) and `c` the vector of spline coefficients. The model function `g` exists for each x coordinate of the input data.

$$g_i(x,\vec{p},\vec{c_i}) = p_2 + p_0 \sum_{m=0}^{3} c_{i,m}(x-i-p_1)^m$$

x (independent variables) *X* coordinates

No independent variables are passed to this model function. Hence, the *X* coordinate of the first data value is assumed to be 0.0. The fit size is *M* data points (M=number of data points in the interface), the *X* coordinates of the data are simply the corresponding array indices of the data array, starting from zero.

p_0 amplitude

p_1 center coordinate

p_2 offset

4.9 2D Spline function

A 2D cubic spline function defined by 4 parameters and a set of cubic spline coefficients. See Gpuspline on Github for details on how to compute the set of cubic spline coefficients from a data set so that it can be used here. The model ID is `SPLINE_2D` and it is implemented in spline_2d.cuh. Here, `p` is the vector of parameters (p0..p3) and `c` the vector of spline coefficients. The model function `g` exists for each x,y coordinate of the input data.

$$g_{i,j}(x, y, \vec{p}, \vec{c}_{i,j}) = p_3 + p_0 \sum_{m=0}^{3} \sum_{n=0}^{3} c_{i,j,m,n} (x - i - p_1)^m (y - j - p_2)^n$$

x,y (independent variables) *X,Y* coordinates

> No independent variables are passed to this model function. Hence, the *(X,Y)* coordinates of the first data value are assumed to be $(0.0, 0.0)$. The fit size is *M x N* data points (M*N=number of data points in the interface), the *(X,Y)* coordinates of the data are simply the corresponding 2D array indices of the data array, starting from zero.

p_0 amplitude

p_1 center coordinate x

p_2 center coordinate y

p_3 offset

4.10 3D Spline function

A 3D cubic spline function defined by 5 parameters and a set of cubic spline coefficients. See Gpuspline on Github for details on how to compute the set of cubic spline coefficients from a data set so that it can be used here. The model ID is `SPLINE_3D` and it is implemented in spline_3d.cuh. Here, `p` is the vector of parameters (p0..p4) and `c` the vector of spline coefficients. The model function `g` exists for each x,y,z coordinate of the input data.

$$g_{i,j,k}(x, y, z, \vec{p}, \vec{c}_{i,j,k}) = p_4 + p_0 \sum_{m=0}^{3} \sum_{n=0}^{3} \sum_{o=0}^{3} c_{i,j,k,m,n,o} (x - i - p_1)^m (y - j - p_2)^n (z - k - p_3)^o$$

x,y,z (independent variables) *X,Y,Z* coordinates

> No independent variables are passed to this model function. Hence, the *(X,Y,Z)* coordinates of the first data value are assumed to be $(0.0, 0.0, 0.0)$. The fit size is *M x N x O* data points (M*N*O=number of data points in the interface), the *(X,Y,Z)* coordinates of the data are simply the corresponding 3D array indices of the data array, starting from zero.

p_0 amplitude

p_1 center coordinate x

p_2 center coordinate y

p_3 center coordinate z

p_4 offset

4.11 3D Multichannel Spline function

A 3D cubic spline function with multiple channels defined by 5 parameters and a set of cubic spline coefficients. See Gpuspline on Github for details on how to compute the set of cubic spline coefficients from a data set so that it can be used here. The model ID is `SPLINE_3D_MULTICHANNEL` and it is implemented in spline_3d_multichannel.cuh. Here, `p` is the vector of parameters (p0..p4) and `c` the vector of spline coefficients. The model function `g` exists for each x,y,z coordinate of the input data.

$$g_{ch,i,j,k}(x,y,z,\vec{p},\vec{c}_{ch,i,j,k}) = p_4 + p_0 \sum_{m=0}^{3}\sum_{n=0}^{3}\sum_{o=0}^{3} c_{ch,i,j,k,m,n,o}(x-i-p_1)^m(y-j-p_2)^n(z-k-p_3)^o$$

x,y,z (independent variables) *X,Y,Z* coordinates

No independent variables are passed to this model function. Hence, the *(X,Y,Z)* coordinates of the first data value are assumed to be $(0.0, 0.0, 0.0)$. The fit size is *M x N x O* data points (M*N*O=number of data points in the interface), the *(X,Y,Z)* coordinates of the data are simply the corresponding 3D array indices of the data array, starting from zero.

p_0 amplitude

p_1 center coordinate x

p_2 center coordinate y

p_3 center coordinate z

p_4 offset

4.12 3D Multichannel Spline function with variable phase

A 3D cubic spline function with multiple channels and a variable phase defined by 6 parameters and a set of cubic spline coefficients. See Gpuspline on Github for details on how to compute the set of cubic spline coefficients from a data set so that it can be used here. The model ID is `SPLINE_3D_PHASE_MULTICHANNEL` and it is implemented in spline_3d_phase_multichannel.cuh. Here, `p` is the vector of parameters (p0..p5) and `c` the vector of spline coefficients. The model function `g` exists for each x,y,z coordinate of the input data.

$$g_{ch,i,j,k}(x,y,z,\vec{p},\vec{c}_{0,ch,i,j,k},\vec{c}_{1,ch,i,j,k},\vec{c}_{2,ch,i,j,k}) = p_4 + p_0\ (f_0 + cos(p5)\ f_1 + sin(p_5)\ f_2)$$

$$f_0 = \sum_{m=0}^{3}\sum_{n=0}^{3}\sum_{o=0}^{3} c_{0,ch,i,j,k,m,n,o}(x-i-p_1)^m(y-j-p_2)^n(z-k-p_3)^o$$

$$f_1 = \sum_{m=0}^{3}\sum_{n=0}^{3}\sum_{o=0}^{3} c_{1,ch,i,j,k,m,n,o}(x-i-p_1)^m(y-j-p_2)^n(z-k-p_3)^o$$

$$f_2 = \sum_{m=0}^{3}\sum_{n=0}^{3}\sum_{o=0}^{3} c_{2,ch,i,j,k,m,n,o}(x-i-p_1)^m(y-j-p_2)^n(z-k-p_3)^o$$

x,y,z (independent variables) *X,Y,Z* coordinates

No independent variables are passed to this model function. Hence, the *(X,Y,Z)* coordinates of the first data value are assumed to be $(0.0, 0.0, 0.0)$. The fit size is *M x N x O* data points (M*N*O=number of data points in the interface), the *(X,Y,Z)* coordinates of the data are simply the corresponding 3D array indices of the data array, starting from zero.

p_0 amplitude

p_1 center coordinate x

p_2 center coordinate y

p_3 center coordinate z

p_4 offset

p_5 phase

CHAPTER

FIVE

ESTIMATOR FUNCTIONS

5.1 Least squares estimator

The least squares estimator computes the weighted sum of the squared deviation between the data values and the model at the positions of the data points. The ID for this estimator is `LSE`. It's implemented in lse.cuh.

Least squares estimation is a common method, and the standard Levenberg-Marquardt algorithm described by Marquardt makes use of minimal least squares. The estimator is described as follows.

$$\chi^2(\vec{p}) = \sum_{n=0}^{N-1} (f_n(\vec{p}) - z_n)^2 \cdot w_n$$

n The index of the data points $(0, .., N-1)$

f_n The model function values at data position n

z_n Data values at data position n

vec{p} Fit model function parameters

w_n Weight values for data at position n

5.2 Maximum likelihood estimator for data subject to Poisson statistics

The maximum likelihood estimator (MLE) for Poisson distributed noise is relatively simple to implement. In the case of data with Poisson noise is provides a more precise estimate when compared to an LSE estimator. The ID for this estimator is `MLE`. It's implemented in mle.cuh.

The estimator is described as follows.

$$\chi^2(\vec{p}) = 2 \sum_{n=0}^{N-1} (f_n(\vec{p}) - z_n) - 2 \sum_{n=0, z_n \neq 0}^{N-1} z_n ln \left(\frac{f_n(\vec{p})}{z_n} \right)$$

n The index of the data points $(0, .., N-1)$

f_n The model function values at data position n

z_n Data values at data position n

vec{p} Actual model function parameters

Note that this estimator does not provide any means to weight the data values. Rather, noise in the data is assumed to be purely Poissonian.

CHAPTER

SIX

EXAMPLES IN C++

Example programs, written in C++, are part of the Gpufit project and can be built and run through the project environment. Here the example programs are described and the important steps in each program are highlighted.

Please note, that additionally, the C++ boost tests, and the Gpufit/Cpufit performance comparison test, may also be of value as example code demonstrating the use of Gpufit. However, a detailed description of these tests programs is not provided.

6.1 Simple example (minimal call to `gpufit()`)

This example demonstrates a simple, minimal program containing all of the required parameters for a call to the Gpufit function. The example is contained in the file Simple_Example.cpp and it can be built and executed within the project environment. Please note that it this code does not actually do anything other than make a single call to gpufit().

In the first section of the code, the *model ID* is set, memory space for initial parameters and data values is allocated, the *fit tolerance* is set, the *maximum number of iterations* is set, the *estimator ID* is set, and the *parameters to fit array* is initialized. Note that in most applications, the data array will already exist and it will be unnecessary to allocate additional space for data. In this example, the *parameters to fit* array is initialized to all ones, indicating that all model parameters should be adjusted in the fit.

```
// number of fits, number of points per fit
size_t const n_fits = 10;
size_t const n_points_per_fit = 10;

// model ID and number of model parameters
int const model_id = GAUSS_1D;
size_t const n_model_parameters = 5;

// initial parameters
std::vector< float > initial_parameters(n_fits * n_model_parameters);

// data
std::vector< float > data(n_points_per_fit * n_fits);

// tolerance
float const tolerance = 0.001f;

// maximum number of iterations
int const max_number_iterations = 10;

// estimator ID
int const estimator_id = LSE;

// parameters to fit (all of them)
std::vector< int > parameters_to_fit(n_model_parameters, 1);
```

In the next section of code, sufficient memory is allocated for the *fit results*, *output states*, *chi-square*, and *number of iterations* arrays.

```
// output parameters
std::vector< float > output_parameters(n_fits * n_model_parameters);
std::vector< int > output_states(n_fits);
std::vector< float > output_chi_square(n_fits);
std::vector< int > output_number_iterations(n_fits);
```

Finally, a call to the C interface of Gpufit is made. In this example, the optional inputs *weights* and *user_info* are not used. The program then checks the return status from Gpufit. If an error occurred, the last error message is obtained and an exception is thrown.

```
// call to gpufit (C interface)
int const status = gpufit
    (
        n_fits,
        n_points_per_fit,
        data.data(),
        0,
        model_id,
        initial_parameters.data(),
        tolerance,
        max_number_iterations,
        parameters_to_fit.data(),
        estimator_id,
        0,
        0,
        output_parameters.data(),
        output_states.data(),
        output_chi_square.data(),
        output_number_iterations.data()
    );

// check status
if (status != STATUS_OK)
{
    throw std::runtime_error(gpufit_get_last_error());
}
```

In summary, the above example illustrates the basic details of the parameters which are passed to the `gpufit()` function, such as the size of the input and output variables, etc. This example could be adapted for real applications by:

- choosing a model ID
- choosing an estimator ID
- setting the fit tolerance and maximum number of iterations
- using a data variable containing the data values to be fit
- providing initial parameters with suitable estimates of the true parameters
- processing the output data

In the following sections, examples are provided in which Gpufit is used to fit simulated datasets.

6.2 Example of 2D Gaussian fits

This example demonstrates the use of Gpufit to fit a dataset consisting of 2D Gaussian peaks. The example is contained in the file Gauss_Fit_2D_Example.cpp and it can be built and executed within the project environment. The optional inputs to gpufit(), *weights* and *user_info*, are not used.

This example features:

- Noisy data and random initial guesses for the fit parameters
- Use of the maximum likelihood estimator which is appropriate for data subject to Poisson noise

In this example, a set of simulated data is generated, consisting of 10^4 individual Gaussian peaks, with a size of 30 x 30 points. Random noise is added to the data. The model function and the model parameters are described in *2D Gaussian function (cylindrical symmetry)* (page 20).

In this example the true parameters used to generate the Gaussian data are defined in the following code block.

```
// true parameters
std::vector< float > true_parameters{ 10.f, 14.5f, 14.5f, 3.f, 10.f}; // amplitude, center x/y positions,␣
↪width, offset
```

These parameters define a 2D Gaussian peak centered at the middle of the grid (position 14.5, 14.5), with a width (standard deviation) of 3.0, an amplitude of 10 and a background of 10. Note that, since we are not providing the independent variables (X values) in the call to Gpufit, the X and Y coordinates of the first data point are assumed to be 0.0, and increasing linearly from this point (i.e. $0, 1, 2, \ldots$).

The guesses for the initial parameters are drawn from the true parameters with a uniformly distributed deviation of about 20%. The initial guesses for the center coordinates are chosen with a deviation relative to the width of the Gaussian.

```
// initial parameters (randomized)
std::vector< float > initial_parameters(n_fits * n_model_parameters);
for (size_t i = 0; i < n_fits; i++)
{
    for (size_t j = 0; j < n_model_parameters; j++)
    {
        if (j == 1 || j == 2)
        {
            initial_parameters[i * n_model_parameters + j] = true_parameters[j] + true_parameters[3] * (-0.
↪2f + 0.4f * uniform_dist(rng));
        }
        else
        {
            initial_parameters[i * n_model_parameters + j] = true_parameters[j] * (0.8f + 0.4f*uniform_
↪dist(rng));
        }
    }
}
```

The 2D grid of X and Y values (each ranging from 0 to 29 with an increment of 1) is computed using a double for loop.

```
// generate x and y values
std::vector< float > x(n_points_per_fit);
std::vector< float > y(n_points_per_fit);
for (size_t i = 0; i < size_x; i++)
{
    for (size_t j = 0; j < size_x; j++) {
        x[i * size_x + j] = static_cast<float>(j);
        y[i * size_x + j] = static_cast<float>(i);
    }
}
```

Next, a 2D Gaussian peak function (without noise) is calculated, once, using the true parameters.

```
void generate_gauss_2d(
    std::vector<float> const & x_coordinates,
    std::vector<float> const & y_coordinates,
    std::vector<float> const & gauss_params,
    std::vector<float> & output_values)
{
    // Generates a Gaussian 2D function at a set of X and Y coordinates.  The Gaussian is defined by
    // an array of five parameters.

    // x_coordinates: Vector of X coordinates.
    // y_coordinates: Vector of Y coordinates.
    // gauss_params:  Vector of function parameters.
    // output_values: Output vector containing the values of the Gaussian function at the
    //                corresponding X, Y coordinates.
```

(continues on next page)

(continued from previous page)

```
    // gauss_params[0]: Amplitude
    // gauss_params[1]: Center X position
    // guass_params[2]: Center Y position
    // gauss_params[3]: Gaussian width (standard deviation)
    // gauss_params[4]: Baseline offset

    // This code assumes that x_coordinates.size == y_coordinates.size == output_values.size

    for (size_t i = 0; i < x_coordinates.size(); i++)
    {

        float arg = -((x_coordinates[i] - gauss_params[1]) * (x_coordinates[i] - gauss_params[1])
                + (y_coordinates[i] - gauss_params[2]) * (y_coordinates[i] - gauss_params[2]))
                / (2.f * gauss_params[3] * gauss_params[3]);

        output_values[i] = gauss_params[0] * exp(arg) + gauss_params[4];

    }
}
```

The variable temp_gauss is used to store the values of the Gaussian peak. This variable is then used as a template to generate a set of Gaussian peaks with random, Poisson-distributed noise.

```
// generate data with noise
std::vector< float > temp_gauss(n_points_per_fit);
// compute the model function
generate_gauss_2d(x, y, true_parameters.begin(), temp_gauss);

std::vector< float > data(n_fits * n_points_per_fit);
for (size_t i = 0; i < n_fits; i++)
{
    // generate Poisson random numbers
    for (size_t j = 0; j < n_points_per_fit; j++)
    {
        std::poisson_distribution< int > poisson_dist(temp_gauss[j]);
        data[i * n_points_per_fit + j] = static_cast<float>(poisson_dist(rng));
    }
}
```

Thus, in this example, the data for each fit differs only in the random noise. This, and the randomized initial guesses for each fit, result in each fit returning slightly different best-fit parameters.

Next, the model and estimator IDs are set, corresponding to the 2D Gaussian fit model function, and the MLE estimator.

```
// estimator ID
int const estimator_id = MLE;

// model ID
int const model_id = GAUSS_2D;
```

Next, the gpufit function is called via the *C Interface* (page 9). Parameters weights, user_info and user_info_size are set to 0, indicating that they are not used in this example.

```
// call to gpufit (C interface)
int const status = gpufit
    (
        n_fits,
        n_points_per_fit,
        data.data(),
        0,
        model_id,
        initial_parameters.data(),
        tolerance,
        max_number_iterations,
        parameters_to_fit.data(),
        estimator_id,
        0,
```

(continues on next page)

(continued from previous page)

```
        0,
        output_parameters.data(),
        output_states.data(),
        output_chi_square.data(),
        output_number_iterations.data()
    );

// check status
if (status != STATUS_OK)
{
    throw std::runtime_error(gpufit_get_last_error());
}
```

After the fits are complete, the return value is checked to ensure that no error occurred.

6.2.1 Output statistics

The last part of this example obtains statistics describing the fit results, and testing whether the fits converged, etc.

The output_states variable contains a state code which indicates whether the fit converged, or if an error occured (see the Gpufit API documentation, *Description of output parameters* (page 12), for details). In this example, a histogram of all possible fit states is obtained by iterating over the state of each fit.

```
// get fit states
std::vector< int > output_states_histogram(5, 0);
for (std::vector< int >::iterator it = output_states.begin(); it != output_states.end(); ++it)
{
    output_states_histogram[*it]++;
}
```

In computing the mean and standard deviation of the results, only the converged fits are taken into account. The following code contains an example of the calculation of the means of the output parameters, iterating over all fits and all model parameters.

```
// compute mean of fitted parameters for converged fits
std::vector< float > output_parameters_mean(n_model_parameters, 0);
for (size_t i = 0; i != n_fits; i++)
{
    if (output_states[i] == STATE_CONVERGED)
    {
        for (size_t j = 0; j < n_model_parameters; j++)
        {
            output_parameters_mean[j] += output_parameters[i * n_model_parameters + j];
        }
    }
}
// normalize
for (size_t j = 0; j < n_model_parameters; j++)
{
    output_parameters_mean[j] /= output_states_histogram[0];
}
```

In summary, the above example illustrates a simple call to `gpufit()` using a real dataset. When the fit is complete, the fit results are obtained and the output states are checked. Additionally, this example calculates some basic statistics describing the results. The code also illustrates how the input and output parameters are organized in memory.

6.3 Linear Regression Example

This example demonstrates the use of Gpufit to compute linear fits to a randomly generated dataset. The example is contained in the file Linear_Regression_Example.cpp and it can be built and executed within the project environment. This example illustrates how independent variables may be used in the fitting process, by taking advantage of the user_info parameter. In this example, a set of 10^4 individual fits are calculated. Each simulated dataset consists of 20 randomly generated data values. The X coordinates of the data points do not have a uniform spacing, but increase non-linearly. The user information data is used to pass the X values to `gpufit()`. The fits are unweighted, and the model function and model parameters are described in *Linear regression* (page 19).

For details of how user_info is used to store the values of the independent variable for this fit model function, see the section of the Gpufit documentation describing the model functions, *Fit Model functions* (page 19).

This example features:

- Noisy data and random initial guesses for the parameters
- Unequally spaced X position values, passed to `gpufit()` using the user_info parameter.

The following code illustrates how the X positions of the data points are stored in the user_info variable, for this model function. The user_info points at a vector of float values. Note, however, that the way in which user_info is used by a model function may vary from function to function.

```
// custom x positions for the data points of every fit, stored in user_info
std::vector< float > user_info(n_points_per_fit);
for (size_t i = 0; i < n_points_per_fit; i++)
{
    user_info[i] = static_cast<float>(pow(2, i));
}

// size of user_info in bytes
size_t const user_info_size = n_points_per_fit * sizeof(float);
```

By providing the data coordinates for only one fit in user_info, the model function will use the same coordinates for all fits in the dataset, as described in *Fit Model functions* (page 19).

In the next section, the initial parameters for each fit are set to random values, uniformly distributed around the true parameter value.

```
// true parameters
std::vector< float > true_parameters { 5, 2 }; // offset, slope

// initial parameters (randomized)
std::vector< float > initial_parameters(n_fits * n_model_parameters);
for (size_t i = 0; i != n_fits; i++)
{
    // random offset
    initial_parameters[i * n_model_parameters + 0] = true_parameters[0] * (0.8f + 0.4f * uniform_
↪dist(rng));
    // random slope
    initial_parameters[i * n_model_parameters + 1] = true_parameters[0] * (0.8f + 0.4f * uniform_
↪dist(rng));
}
```

The data is then generated as the value of a linear function plus additive, normally distributed, random noise.

```
// generate data
std::vector< float > data(n_points_per_fit * n_fits);
for (size_t i = 0; i != data.size(); i++)
{
    size_t j = i / n_points_per_fit; // the fit
    size_t k = i % n_points_per_fit; // the position within a fit

    float x = user_info[k];
    float y = true_parameters[0] + x * true_parameters[1];
    data[i] = y + normal_dist(rng);
}
```

In the following code, the model and estimator IDs for the fit are initialized.

```
// estimator ID
int const estimator_id = LSE;

// model ID
int const model_id = LINEAR_1D;
```

Finally, a call is made to `gpufit()` (*C Interface* (page 9)). The weights parameter is set to 0, indicating that the fits are unweighted.

```
// call to gpufit (C interface)
int const status = gpufit
    (
        n_fits,
        n_points_per_fit,
        data.data(),
        0,
        model_id,
        initial_parameters.data(),
        tolerance,
        max_number_iterations,
        parameters_to_fit.data(),
        estimator_id,
        user_info_size,
        reinterpret_cast< char * >( user_info.data() ),
        output_parameters.data(),
        output_states.data(),
        output_chi_square.data(),
        output_number_iterations.data()
    );
```

After the fits have been executed and the return value is checked to ensure that no error occurred, statistics describing the fit results are calculated and displayed, as in the previous example (see *Output statistics* (page 30)).

CHAPTER

SEVEN

CUSTOMIZATION

This sections explains how to add custom fit model functions and custom fit estimators within the Gpufit library. Functions calculating the estimator and model values are CUDA device functions that are defined in CUDA header files using the C syntax. For each function and estimator there exists a separate file. Therefore, to add an additional model or estimator a new CUDA header file containing the new model or estimator function must be created and included in the library.

Please note, that in order to add a model function or estimator, it is necessary to rebuild the Gpufit library from source. In future releases of Gpufit, it may be possible to include new fit functions or estimators at runtime.

7.1 Add a new fit model function

To add a new fit model, the model function itself as well as analytic expressions for its partial derivatives must to be known. A function calculating the values of the model as well as a function calculating the values of the partial derivatives of the model, with respect to the model parameters and possible grid coordinates, must be implemented.

Additionally, a new model ID must be defined and included in the list of available model IDs, and the number of model parameters and dimensions must be specified as well.

Detailed step by step instructions for adding a model function are given below.

1. Define an additional model ID in file constants.h. When using the language bindings, the model ID must also be added for Python (in gpufit.py), Matlab (in ModelID.m), Java (in Model.java).
2. Implement a CUDA device function within a newly created .cuh file in folder Gpufit/Gpufit/models according to the following template.

```
__device__ void ... (                // ... = function name
    float const * parameters,
    int const n_fits,
    int const n_points,
    float * value,
    float * derivative,
    int const point_index,
    int const fit_index,
    int const chunk_index,
    char * user_info,
    std::size_t const user_info_size)
{
    ///////////////////////////// value //////////////////////////////

    value[point_index] = ... ;                      // formula calculating fit model values

    /////////////////////////// derivative ///////////////////////////
    float * current_derivative = derivative + point_index;

    current_derivative[0 * n_points] = ... ;  // formula calculating derivative values with respect to
↪parameters[0]
    current_derivative[1 * n_points] = ... ;  // formula calculating derivative values with respect to
↪parameters[1]
    .
    .
```

(continues on next page)

(continued from previous page)

```
    .
}
```

This code can be used as a pattern, where the placeholders "..." must be replaced by user code which calculates model function values and partial derivative values of the model function for a particular set of parameters. See for example linear_1d.cuh.

3. Include the newly created .cuh file in models.cuh
4. Add a switch case in the CUDA device function `calculate_model()` in file models.cuh to allow calling the newly added model function.

```
switch (model_id)
{
case GAUSS_1D:
    calculate_gauss1d(parameters, n_fits, n_points, value, derivative, point_index, fit_index, chunk_index,
↪ user_info, user_info_size);
    break;
    .
    .
    .
case ...:                           // model ID
    ...                             // function name
        (parameters, n_fits, n_points, value, derivative, point_index, fit_index, chunk_index, user_info,␣
↪user_info_size);
    break;
    .
    .
    .
default:
    break;
}
```

5. Add a switch case in function `configure_model()` in file models.cuh.

```
switch (model_id)
{
case GAUSS_1D:              n_parameters = 4; n_dimensions = 1; break;
.
.
.
case ...:                   // model ID
    n_parameters = ...;     // number of model parameters
    n_dimensions = ...;     // number of model dimensions
    break;

default:                                                        break;
}
```

6. After adding a new model function, if CMake is being used to configure the compiler, then CMake must be run again. If not using CMake, the new model function file (the .cuh file) must be included in the project.
7. Re-build the Gpufit project.

7.2 Add a new fit estimator

To extend the Gpufit library with additional estimators, three CUDA device functions must be defined and integrated. The sections requiring modification are the functions which calculate the estimator function values, and its gradient and hessian values. Also, a new estimator ID must be defined. Detailed step by step instructions for adding an additional estimator is given below.

1. Define an additional estimator ID in constants.h When using the language bindings, the estimator ID must be added also for Python (in gpufit.py), Matlab (in EstimatorID.m), Java (in Estimator.java).
2. Implement three functions within a newly created .cuh file in the folder Gpufit/Gpufit/estimators calculating χ^2 values and its gradient and hessian according to the following template.

```
///////////////////////////// Chi-square ////////////////////////////
__device__ void ... (           // ... = function name Chi-square
    volatile float * chi_square,
    int const point_index,
    float const * data,
    float const * value,
    float const * weight,
    int * state,
    char * user_info,
    std::size_t const user_info_size)
{
    chi_square[point_index] = ... ;            // formula calculating Chi-square summands
}

////////////////////////////// gradient ////////////////////////////
__device__ void ... (           // ... = function name gradient of Chi-square
    volatile float * gradient,
    int const point_index,
    int const parameter_index,
    float const * data,
    float const * value,
    float const * derivative,
    float const * weight,
    char * user_info,
    std::size_t const user_info_size)
{
    gradient[point_index] = ... ;           // formula calculating summands of the gradient of Chi-square
                                            // model derivates are stored in derivative[parameter_index]
}

////////////////////////////// hessian /////////////////////////////
__device__ void ... (           // function name hessian
    double * hessian,
    int const point_index,
    int const parameter_index_i,
    int const parameter_index_j,
    float const * data,
    float const * value,
    float const * derivative,
    float const * weight,
    char * user_info,
    std::size_t const user_info_size)
{
    *hessian += ... ;            // formula calculating summands of the hessian of Chi-square
}
```

This code can be used as a pattern, where the placeholders "…" must be replaced by user code which calculates the estimator and the gradient and hessian values of the estimator given. For a concrete example, see lse.cuh.

3. Include the newly created .cuh file in estimators.cuh.

```
#include "....cuh"              // filename
```

4. Add a switch case in three CUDA device functions in the file estimators.cuh.

   4a. Calculation of Chi-square:

```
switch (estimator_id)
{
case LSE:
    calculate_chi_square_lse(chi_square, point_index, data, value, weight, state,␣
↪user_info, user_info_size);
    break;
    .
    .
    .
case ...:             // estimator ID
    ...               // function name Chi-square
        (chi_square, point_index, data, value, weight, state, user_info, user_info_
↪size);
    break;
```

(continues on next page)

(continued from previous page)

```
default:
    break;
}
```

4b. Calculation of the gradients of Chi-square:

```
switch (estimator_id)
{
case LSE:
    calculate_gradient_lse(gradient, point_index, parameter_index, data, value,␣
↪derivative, weight, user_info, user_info_size);
    break;
    .
    .
    .
case ...:           // estimator ID
    ...             // function name gradient
        (gradient, point_index, parameter_index, data, value, derivative, weight,␣
↪user_info, user_info_size);
    break;

default:
    break;
}
```

4c. Calculation of the Hessian:

```
switch (estimator_id)
{
case LSE:
    calculate_hessian_lse
        (hessian, point_index, parameter_index_i, parameter_index_j, data, value,␣
↪derivative, weight, user_info,user_info_size);
    break;
    .
    .
    .
case ...:           // estimator ID
    ...             // function name hessian
        (hessian, point_index, parameter_index_i, parameter_index_j, data, value,␣
↪derivative, weight, user_info, user_info_size);
    break;

default:
    break;
}
```

5. After adding a new estimator, if CMake is being used to configure the compiler, then CMake must be run again. If not using CMake, the new estimator file (the .cuh file) must be included in the project.
6. Re-build the Gpufit project.

7.3 Future releases

A current disadvantage of the Gpufit library, when compared with established CPU-based curve fitting packages, is that in order to add or modify a fit model function or a fit estimator, the library must be recompiled. We anticipate that this limitation can be overcome in future releases of the library, by employing run-time compilation of the CUDA code.

CHAPTER

EIGHT

EXTERNAL BINDINGS

This sections describes the Gpufit bindings to other programming languages. The bindings (to Python, Matlab or Java) aim to emulate the *C Interface* (page 9) as closely as possible.

Most high level languages feature multidimensional numeric arrays. In the bindings implemented for Matlab and Python, we adopt the convention that the input data should be organized as a 2D array, with one dimension corresponding to the number of data points per fit, and the other corresponding to the number of fits. Internally, in memory, these arrays should always be ordered such that the data values for each fit are kept together. In Matlab, for example, this means storing the data in an array with dimensions [number_points_per_fit, number_fits]. In this manner, the data in memory is ordered in the same way that is expected by the Gpufit C interface, and there is no need to copy or otherwise re-organize the data before passing it to the GPU. The same convention is used for the weights, the initial model parameters, and the output parameters.

In Java we pre-allocate one dimensional FloatBuffers or IntBuffers for the data and the fit results. The user is responsible for copying data into these buffers.

Unlike the C interface, the external bindings do not require the number of fits and the number of data points per fit to be specified explicitly. Instead, these numbers are inferred from the dimensions of the 2D input arrays.

8.1 Optional parameters with default values

The external bindings make some input parameters optional. The optional parameters are shown here. They are kept the same for all bindings.

tolerance default value 1e-4

max_n_iterations default value 25 iterations

estimator_id the default estimator is LSE as defined in constants.h

parameters_to_fit by default all parameters are fit

For instructions on how to specify these parameters explicitly, see the sections below.

8.2 Python

The Gpufit binding for Python is a project named pyGpufit. This project contains a Python package named pygpufit, which contains a module gpufit, and this module implements a method called fit. Calling this method is equivalent to calling the C interface function `gpufit()` of the Gpufit library. The package expects the input data to be stored as NumPy array. NumPy follows row-major order by default.

8.2.1 Installation

Wheel files for Python 2.X and 3.X on Windows 32/64 bit are included in the binary package. NumPy is required.

Install the wheel file with.

```
pip install --no-index --find-links=LocalPathToWheelFile pyGpufit
```

8.2.2 Python Interface

fit

The signature of the fit method (equivalent to calling the C interface function `gpufit()`) is

```
def fit(data, weights, model_id:ModelID, initial_parameters, tolerance:float=None, max_number_
↪iterations:int=None, parameters_to_fit=None, estimator_id:EstimatorID=None, user_info=None):
```

Optional parameters are passed in as None. The numbers of points, fits and parameters is deduced from the dimensions of the input data and initial parameters arrays.

Input parameters

data Data 2D NumPy array of shape (number_fits, number_points) and data type np.float32

weights Weights 2D NumPy array of shape (number_fits, number_points) and data type np.float32 (same as data)

special None indicates that no weights are available

tolerance Fit tolerance

type float

special If None, the default value will be used.

max_number_iterations Maximal number of iterations

type int

special If None, the default value will be used.

estimator_id estimator ID

type EstimatorID which is an Enum in the same module and defined analogously to constants.h.

special If None, the default value is used.

model_id model ID

type ModelID which is an Enum in the same module and defined analogously to constants.h.

initial_parameters Initial parameters 2D NumPy array of shape (number_fits, number_parameter)

array data type np.float32

parameters_to_fit parameters to fit 1D NumPy array of length number_parameter A zero indicates that this parameter should not be fitted, everything else means it should be fitted.

array data type np.int32

special If None, the default value is used.

user_info user info 1D NumPy array of arbitrary type. The length in bytes is deduced automatically.

special If None, no user_info is assumed.

Output parameters

parameters Fitted parameters for each fit 2D NumPy array of shape (number_fits, number_parameter) and data type np.float32

states Fit result states for each fit 1D NumPy array of length number_parameter of data type np.int32 As defined in constants.h:

chi_squares χ^2 values for each fit 1D NumPy array of length number_parameter of data type np.float32

n_iterations Number of iterations done for each fit 1D NumPy array of length number_parameter of data type np.int32

time Execution time of call to fit In seconds.

Errors are raised if checks on parameters fail or if the execution of fit failed.

fit_constrained

The `fit_constrained` method is very similar to the `fit` method with the additional possibility to specify parameter constraints.

The signature of the `fit_constrained` method (equivalent to calling the C interface function `gpufit_constrained()`) is

```
def fit_constrained(data, weights, model_id:ModelID, initial_parameters, constraints=None, constraint_
↪types=None, tolerance:float=None, max_number_iterations:int=None, parameters_to_fit=None, estimator_
↪id:EstimatorID=None, user_info=None):
```

Constraint input parameters

constraints Constraint bound intervals for every parameter and every fit. 2D NumPy array of shape (number_fits, 2*number_parameter) and data type np.float32

contraint_types Constraint types for every parameter 1D NumPy array of length number_parameter Valid values are defined in gf.ConstraintType

get_last_error

The signature of the get_last_error method (equivalent to calling the C interface function *gpufit_get_last_error*) is

```
def get_last_error():
```

Returns a string representing the error message of the last occurred error.

cuda_available

The signature of the cuda_available method (equivalent to calling the C interface function *gpufit_cuda_available*) is

```
def cuda_available():
```

Returns True if CUDA is available and False otherwise.

get_cuda_version

The signature of the get_cuda_version method (equivalent to calling the C interface function *gpufit_get_cuda_version*) is

```
def get_cuda_version():
```

Output parameters

runtime version Tuple of (Major version, Minor version)

driver version Tuple of (Major version, Minor version)

An error is raised if the execution failed (i.e. because CUDA is not available).

8.2.3 Python Examples

2D Gaussian peak example

An example can be found at Python Gauss2D example. It is equivalent to *Example of 2D Gaussian fits* (page 27).

The essential imports are:

```
import numpy as np
import pygpufit.gpufit as gf
```

First we test for availability of CUDA as well as CUDA driver and runtime versions.

```
# cuda available checks
print('CUDA available: {}'.format(gf.cuda_available()))
print('CUDA versions runtime: {}, driver: {}'.format(*gf.get_cuda_version()))
```

The true parameters describing an example 2D Gaussian peak functions are:

```
# true parameters
true_parameters = np.array((10, 5.5, 5.5, 3, 10), dtype=np.float32)
```

A 2D grid of x and y positions can conveniently be generated using the np.meshgrid function:

```
# generate x and y values
g = np.arange(size_x)
yi, xi = np.meshgrid(g, g, indexing='ij')
xi = xi.astype(np.float32)
yi = yi.astype(np.float32)
```

Using these positions and the true parameter values a model function can be calculated as

```
def generate_gauss_2d(p, xi, yi):
    """
    Generates a 2D Gaussian peak.
    http://gpufit.readthedocs.io/en/latest/api.html#gauss-2d

    :param p: Parameters (amplitude, x,y center position, width, offset)
    :param xi: x positions
    :param yi: y positions
    :return: The Gaussian 2D peak.
    """

    arg = -(np.square(xi - p[1]) + np.square(yi - p[2])) / (2*p[3]*p[3])
    y = p[0] * np.exp(arg) + p[4]

    return y
```

The model function can be repeated and noise can be added using the np.tile and np.random.poisson functions.

```
# generate data
data = generate_gauss_2d(true_parameters, xi, yi)
data = np.reshape(data, (1, number_points))
data = np.tile(data, (number_fits, 1))

# add Poisson noise
data = np.random.poisson(data)
data = data.astype(np.float32)
```

The model and estimator IDs can be set as

```
# estimator ID
estimator_id = gf.EstimatorID.MLE

# model ID
model_id = gf.ModelID.GAUSS_2D
```

When all input parameters are set we can call the C interface of Gpufit.

```
# run Gpufit
parameters, states, chi_squares, number_iterations, execution_time = gf.fit(data, None, model_id, initial_
↪parameters, tolerance, max_number_iterations, None, estimator_id, None)
```

And finally statistics about the results of the fits can be displayed where the mean and standard deviation of the fitted parameters are limited to those fits that converged.

```
# print fit results

# get fit states
converged = states == 0
number_converged = np.sum(converged)
print('ratio converged         {:6.2f} %'.format(number_converged / number_fits * 100))
print('ratio max it. exceeded  {:6.2f} %'.format(np.sum(states == 1) / number_fits * 100))
print('ratio singular hessian  {:6.2f} %'.format(np.sum(states == 2) / number_fits * 100))
print('ratio neg curvature MLE {:6.2f} %'.format(np.sum(states == 3) / number_fits * 100))
print('ratio gpu not read      {:6.2f} %'.format(np.sum(states == 4) / number_fits * 100))

# mean, std of fitted parameters
converged_parameters = parameters[converged, :]
converged_parameters_mean = np.mean(converged_parameters, axis=0)
converged_parameters_std = np.std(converged_parameters, axis=0)

for i in range(number_parameters):
    print('p{} true {:6.2f} mean {:6.2f} std {:6.2f}'.format(i, true_parameters[i], converged_parameters_
↪mean[i], converged_parameters_std[i]))

# print summary
print('model ID: {}'.format(model_id))
print('number of fits: {}'.format(number_fits))
print('fit size: {} x {}'.format(size_x, size_x))
print('mean chi_square: {:.2f}'.format(np.mean(chi_squares[converged])))
print('iterations: {:.2f}'.format(np.mean(number_iterations[converged])))
print('time: {:.2f} s'.format(execution_time))
```

2D Gaussian peak constrained fit example

An example for a constrained fit can be found at Python Gauss2D constrained fit example. It differs from the previous example only in that constraints are specified additionally (as 2D array of lower and upper bounds on parameters for every fit) as well as constraint types (for all parameters including fixed parameters) that can take a value of ConstraintType (FREE, LOWER, UPPER or LOWER_UPPER) in order to either do not enforce the constraints for a parameter or enforce them only at the lower or upper or both bounds.

The following code block demonstrates how the sigma of a 2D Gaussian peak can be constrained to the interval [2.9, 3.1] and the background and ampltiude to non-negative values.

```
# set constraints
constraints = np.zeros((number_fits, 2*number_parameters), dtype=np.float32)
constraints[:, 6] = 2.9
constraints[:, 7] = 3.1
constraint_types = np.array([gf.ConstraintType.LOWER, gf.ConstraintType.FREE, gf.ConstraintType.FREE, gf.
↪ConstraintType.LOWER_UPPER, gf.ConstraintType.LOWER], dtype=np.int32)

# run constrained Gpufit
parameters, states, chi_squares, number_iterations, execution_time = gf.fit_constrained(data, None, model_
↪id,
                                                                                            initial_parameters,␣
↪constraints, constraint_types,
                                                                                            tolerance, max_number_
↪iterations, None,
                                                                                            estimator_id, None)
```

8.3 Matlab

The Matlab binding for Gpufit is a Matlab script (gpufit.m). This script checks the input data, sets default parameters, and calls the C interface of the Gpufit library, via a compiled .mex file.

Please note, that before using the Matlab binding, the path to gpufit.m must be added to the Matlab path.

If other GPU-based computations are to be performed with Matlab in the same session, please use the Matlab GPU computing functionality first (for example with a call to gpuDevice or gpuArray) before calling the Gpufit Matlab binding. If this is not done, Matlab will throw an error (Error using gpuArray An unexpected error occurred during CUDA execution. The CUDA error was: cannot set while device is active in this process).

8.3.1 Matlab Interface

gpufit

Optional parameters are passed in as empty matrices (`[]`). The numbers of points, fits and parameters is deduced from the dimensions of the input data and initial parameters matrices.

The signature of the `gpufit` function is

```
function [parameters, states, chi_squares, n_iterations, time] = gpufit(data, weights, model_id, initial_
↪parameters, tolerance, max_n_iterations, parameters_to_fit, estimator_id, user_info)
```

Input parameters

data Data 2D matrix of size [number_points, number_fits] and data type single

weights Weights 2D matrix of size [number_points, number_fits] and data type single (same as data)

 special None indicates that no weights are available

tolerance Fit tolerance

 type single

 special If empty ([]), the default value will be used.

max_number_iterations Maximal number of iterations Will be converted to int32 if necessary

 special If empty ([]), the default value will be used.

estimator_id estimator ID

 type EstimatorID which is defined in EstimatorID.m analogously to constants.h.

 special If empty ([]), the default value is used.

model_id model ID

type ModelID which is defined in ModelID.m analogously to constants.h.

initial_parameters Initial parameters 2D matrix of size: [number_parameter, number_fits]

type single

parameters_to_fit parameters to fit vector of length number_parameter, will be converted to int32 if necessary A zero indicates that this parameter should not be fitted, everything else means it should be fitted.

special If empty ([]), the default value is used.

user_info user info vector of suitable type (correct type is not checked and depends on the chosen fit model function or estimator). The length of user_info in bytes is determined automatically.

Output parameters

parameters Fitted parameters for each fit 2D matrix of size: [number_parameter, number_fits] of data type single

states Fit result states for each fit vector of length number_parameter of data type int32 As defined in constants.h:

chi_squares χ^2 values for each fit vector of length number_parameter of data type single

n_iterations Number of iterations done for each fit vector of length number_parameter of data type int32

time Execution time of call to gpufit In seconds.

Errors are raised if checks on parameters fail or if the execution of gpufit fails.

gpufit_constrained

The `gpufit_constrained` function is very similar to the `gpufit` function with the additional possibility to specify parameter constraints.

The signature of the `gpufit_constrained` function is

```
function [parameters, states, chi_squares, n_iterations, time] = gpufit_constrained(data, weights, model_
↪id, initial_parameters, constraints, constraint_types, tolerance, max_n_iterations, parameters_to_fit,␣
↪estimator_id, user_info)
```

Constraint input parameters

constraints Constraint bound intervals for every parameter and every fit 2D matrix of size [2*number_parameter, number_fits] of data type single

contraint_types Constraint types for every parameter Vector of length number_parameter, will be converted to int32 if necessary. Valid values are defined in ConstraintType.m.

gpufit_cuda_available

The signature of the gputfit_cuda_available method (equivalent to calling the C interface function *gpufit_cuda_available*) is

```
function r = gpufit_cuda_available():
```

Returns True if CUDA is available and False otherwise.

8.3.2 Matlab Examples

Simple example

The most simple example is the Matlab simple example. It is equivalent to *Simple example (minimal call to gpufit())* (page 26) and additionally relies on default values for optional arguments.

2D Gaussian peak example

An example can be found at Matlab Gauss2D example. It is equivalent to *Example of 2D Gaussian fits* (page 27).

The true parameters describing an example 2D Gaussian peak functions are:

```
% true parameters
true_parameters = single([10, 5.5, 5.5, 3, 10]);
```

A 2D grid of x and y positions can conveniently be generated using the ndgrid function:

```
% generate x and y values
g = single(0 : size_x - 1);
[x, y] = ndgrid(g, g);
```

Using these positions and the true parameter values a model function can be calculated as

```
function g = gaussian_2d(x, y, p)
% Generates a 2D Gaussian peak.
% http://gpufit.readthedocs.io/en/latest/api.html#gauss-2d
%
% x,y - x and y grid position values
% p - parameters (amplitude, x,y center position, width, offset)

g = p(1) * exp(-((x - p(2)).^2 + (y - p(3)).^2) / (2 * p(4)^2)) + p(5);

end
```

The model function can be repeated and noise can be added using the repmat and poissrnd functions.

```
% generate data with Poisson noise
data = gaussian_2d(x, y, true_parameters);
data = repmat(data(:), [1, number_fits]);
data = poissrnd(data);
```

The model and estimator IDs can be set as

```
% estimator id
estimator_id = EstimatorID.MLE;

% model ID
model_id = ModelID.GAUSS_2D;
```

When all input parameters are set we can call the C interface of the Gpufit library.

```
%% run Gpufit
[parameters, states, chi_squares, n_iterations, time] = gpufit(data, [], model_id, initial_parameters,␣
↪tolerance, max_n_iterations, [], estimator_id, []);
```

And finally statistics about the results of the fits can be displayed where the mean and standard deviation of the fitted parameters are limited to those fits that converged.

```
%% displaying results

% get fit states
converged = states == 0;
number_converged = sum(converged);
fprintf(' ratio converged         %6.2f %%\n', number_converged / number_fits * 100);
```

(continues on next page)

(continued from previous page)

```
fprintf(' ratio max it. exceeded  %6.2f %%\n', sum(states == 1) / number_fits * 100);
fprintf(' ratio singular hessian  %6.2f %%\n', sum(states == 2) / number_fits * 100);
fprintf(' ratio neg curvature MLE %6.2f %%\n', sum(states == 3) / number_fits * 100);
fprintf(' ratio gpu not read      %6.2f %%\n', sum(states == 4) / number_fits * 100);

% mean and std of fitted parameters
converged_parameters = parameters(:, converged);
converged_parameters_mean = mean(converged_parameters, 2);
converged_parameters_std  = std(converged_parameters, [], 2);
for i = 1 : number_parameters
    fprintf(' p%d true %6.2f mean %6.2f std %6.2f\n', i, true_parameters(i), converged_parameters_mean(i),␣
↪converged_parameters_std(i));
end

% print summary
fprintf('model ID: %d\n', model_id);
fprintf('number of fits: %d\n', number_fits);
fprintf('fit size: %d x %d\n', size_x, size_x);
fprintf('mean chi-square: %6.2f\n', mean(chi_squares(converged)));
fprintf('iterations: %6.2f\n', mean(n_iterations(converged)));
fprintf('time: %6.2f s\n', time);
```

2D Gaussian peak constrained fit example

An example for a constrained fit can be found at Matlab Gauss2D constrained fit example. It differs from the previous example only in that constraints are specified additionally (as 2D array of lower and upper bounds on parameters for every fit) as well as constraint types (for all parameters including fixed parameters) that can take a value of ConstraintType (FREE, LOWER, UPPER or LOWER_UPPER) in order to either do not enforce the constraints for a parameter or enforce them only at the lower or upper or both bounds.

The following code block demonstrates how the sigma of a 2D Gaussian peak can be constrained to the interval [2.9, 3.1] and the background and amplitude to non-negative values.

```
%% set constraints
constraints = zeros([2*number_parameters, number_fits], 'single');
constraints(7, :) = 2.9;
constraints(8, :) = 3.1;
constraint_types = int32([ConstraintType.LOWER, ConstraintType.FREE, ConstraintType.FREE, ConstraintType.
↪LOWER_UPPER, ConstraintType.LOWER]);

%% run constrained Gpufit
[parameters, states, chi_squares, n_iterations, time] = gpufit_constrained(data, [], ...
    model_id, initial_parameters, constraints, constraint_types, tolerance, max_n_iterations, [],␣
↪estimator_id, []);
```

8.4 Java

The Gpufit binding for Java consists of a small adapter C library named GpufitJNI and a Gpufit jar archive containing a com.github.gpufit package. In these the class Gpufit has static methods largely equivalent to calling the C interface function `gpufit()` of the Gpufit library. The fit method expects the input to be given as a FitModel instance, which among other things specifies the model and the estimator as enums. The results are returned as a FitResult instance.

8.4.1 Installation

Build the Gpufit library and the GpufitJNI library from source as documented in *Installation and Testing* (page 3). Make sure both libraries are in the Java library path, for example by using the -Djava.library.path comman line switch for the VM.

Build the Gpufit.jar from the sources using Gradle on Gpufit/java/gpufit/build.gradle. Make sure this jar is in the Java class path of your application, e.g. by adding it as a dependency to your project

8.4.2 Java Interface

For a more complete description, see the Javadoc output of the Gpufit Java binding project.

Gpufit.fit

The signature of the fit method (calls the C interface function `gpufit()`) is

```
public static FitResult fit(FitModel fitModel, FitResult fitResult)
```

Input parameters are given as a FitModel, output parameters are stored in a FitResult. A FitResult can be re-used if the number of fits and the number of parameters of the model didn't change. It must then also be given as second parameter.

Input of the fit - Filling the FitModel

```
public FitModel(int numberFits, int numberPoints, boolean withWeights, Model model, Float tolerance,␣
↪Integer maxNumberIterations, Boolean[] parametersToFit, Estimator estimator, int userInfoSize)
```

numberFits Number of fits

numberPoints Number of data points per fit

widthWeights If true, a buffer for giving weights is pre-allocated, otherwise not

model An enum describing the model. See class Model for more information. Naming and id is equivalent to the C code.

tolerance Fit tolerance

special If null, the default value will be used.

maxNumberIterations Maximal number of iterations

special If null, the default value will be used.

parametersToFit Boolean array indicating which parameters should be fitted

special If null, the default value will be used.

estimator Enum describing the estimator function. See class Estimator for more information. Naming and id is equivalent to the C code.

special If None, the default value is used.

userInfoSize The size of the user info (in bytes).

special Must be positive, otherwise the buffer for user info is not pre-allocated.

Afterwards the buffers for data, weights (if desired), initial parameters and user info (if desired) must be filled with the appropriate content. The internal layout is the same as in the C part of Gpufit, i.e. the data represents an 1D number array of length of number fits times number data points per fit with an order of data points followed one after another for all fits. In this batch. The initial parameters are number fits times number of parameters in the model with the parameters for each fit changing fastest and the number of fits slowest.

Fit output - The FitResult

Memory for the fit output is either created automatically or a previous instance of FitResult can be reused to avoid recreation.

```
public class FitResult {

    public final FloatBuffer parameters;
    public final IntBuffer states;
    public final FloatBuffer chiSquares;
    public final IntBuffer numberIterations;
    public float fitDuration;
```

parameters Fitted parameters for each fit

states Fit result states for each fit As defined in constants.h:

chi_squares χ^2 values for each fit

n_iterations Number of iterations done for each fit

time Execution time of call to fit In seconds.

Errors are raised if checks on parameters fail or if the execution of fit failed.

Gpufit.getLastError

The signature of the get_last_error method (equivalent to calling the C interface function *gpufit_get_last_error*) is

```
public static native String getLastError()
```

Returns a string representing the error message of the last occurred error.

Gpufit.isCudaAvailable

The signature of the cuda_available method (equivalent to calling the C interface function *gpufit_cuda_available*) is

```
public static native boolean isCudaAvailable()
```

Returns True if CUDA is available and False otherwise.

get_cuda_version

The signature of the get_cuda_version method (equivalent to calling the C interface function *gpufit_get_cuda_version*) is

```
public static CudaVersion getCudaVersion()
```

The output is a CudaVersion instance with two simple member variables.

runtime version String of "Major version.Minor version"

driver version String of "Major version.Minor version"

An error is raised if the execution failed (i.e. because CUDA is not available).

8.4.3 Java Example

2D Gaussian peak example

An example can be found at Java Gauss2D example. It is equivalent to *Example of 2D Gaussian fits* (page 27).

First we test for availability of CUDA as well as CUDA driver and runtime versions.

```
// print general CUDA information
System.out.println(String.format("CUDA available: %b", Gpufit.isCudaAvailable()));
CudaVersion cudaVersion = Gpufit.getCudaVersion();
System.out.println(String.format("CUDA versions runtime: %s, driver: %s", cudaVersion.runtime, cudaVersion.
→driver));
```

The model and estimator IDs can be set as

```
Model model = Model.GAUSS_2D;
Estimator estimator = Estimator.MLE;
```

The true parameters describing an example 2D Gaussian peak functions are:

```
// true parameters (order: amplitude, center-x, center-y, width, offset)
float[] trueParameters = new float[]{10, 5.5f, 5.5f, 3, 10};
```

A 2D grid of x and y positions can conveniently be generated:

```
// generate x and y values
float[] xi = new float[numberPoints];
float[] yi = new float[numberPoints];
for (int i = 0; i < sizeX; i++) {
    for (int j = 0; j < sizeX; j++) {
        xi[i * sizeX + j] = i;
        yi[i * sizeX + j] = j;
    }
}
```

Using these positions and the true parameter values a model function can be calculated as

```
/**
 * Computes a 2D Gaussian peak given x and y values and parameters.
 *
 * See also: http://gpufit.readthedocs.io/en/latest/api.html#gauss-2d
 *
 * @param p Parameter array
 * @param x x values array
 * @param y y values array
 * @return Model values array
 */
private static float[] generateGauss2D(float[] p, float[] x, float[] y) {
    // checks
    assert(x.length == y.length);
    assert(p.length == 5);

    // calculate data
    float[] data = new float[x.length];
    for (int i = 0; i < x.length; i++) {
        float arg = -((x[i] - p[1]) * (x[i] - p[1]) + (y[i] - p[2]) * (y[i] - p[2])) / (2 * p[3] * p[3]);
        data[i] = p[0] * (float)Math.exp(arg) + p[4];
    }
    return data;
}
```

The model function can be repeated and Poisson noise can be added.

```
// generate data
float[] gauss2D = generateGauss2D(trueParameters, xi, yi);
float[] data = new float[numberFits * numberPoints];
for (int i = 0; i < numberFits; i++) {
```

(continues on next page)

(continued from previous page)

```
    System.arraycopy(gauss2D, 0, data, i * numberPoints, numberPoints);
}

// add Poisson noise
for (int i = 0; i < numberFits * numberPoints; i++) {
    data[i] = nextPoisson(data[i], rand);
}
```

A FitModel containing all the input data including copying the data values from an array to a Java buffer can be done via

```
// assemble FitModel
FitModel fitModel = new FitModel(numberFits, numberPoints, false, model, tolerance, maxNumberIterations,␣
↪null, estimator, 0);

// fill data and initial parameters in the fit model
fitModel.data.clear();
fitModel.data.put(data);
fitModel.initialParameters.clear();
fitModel.initialParameters.put(initialParameters);
```

When all input parameters are set we can call the C interface of Gpufit.

```
// fun Gpufit
FitResult fitResult = Gpufit.fit(fitModel);
```

And finally statistics about the results of the fits can be displayed where the mean and standard deviation of the fitted parameters are limited to those fits that converged.

```
// count FitState outcomes and get a list of those who converged
boolean[] converged = new boolean[numberFits];
int numberConverged = 0, numberMaxIterationExceeded = 0, numberSingularHessian = 0,␣
↪numberNegativeCurvatureMLE = 0;
for (int i = 0; i < numberFits; i++) {
    FitState fitState = FitState.fromID(fitResult.states.get(i));
    converged[i] = fitState.equals(FitState.CONVERGED);
    switch (fitState) {
        case CONVERGED:
            numberConverged++;
            break;
        case MAX_ITERATIONS:
            numberMaxIterationExceeded++;
            break;
        case SINGULAR_HESSIAN:
            numberSingularHessian++;
            break;
        case NEG_CURVATURE_MLE:
            numberNegativeCurvatureMLE++;
    }
}

// get mean and std of converged parameters
float [] convergedParameterMean = new float[]{0, 0, 0, 0, 0};
float [] convergedParameterStd = new float[]{0, 0, 0, 0, 0};
for (int i = 0; i < numberFits; i++) {
    for (int j = 0; j < model.numberParameters; j++) {
        if (converged[i]) {
            convergedParameterMean[j] += fitResult.parameters.get(i * model.numberParameters + j);
        }
    }
}
for (int i = 0; i < model.numberParameters; i++) {
    convergedParameterMean[i] /= numberConverged;
}
for (int i = 0; i < numberFits; i++) {
    for (int j = 0; j < model.numberParameters; j++) {
        if (converged[i]) {
            float dev = fitResult.parameters.get(i * model.numberParameters + j) -␣
↪convergedParameterMean[j];
```

(continues on next page)

(continued from previous page)

```
            convergedParameterStd[j] += dev * dev;
        }
    }
}
for (int i = 0; i < model.numberParameters; i++) {
    convergedParameterStd[i] = (float)Math.sqrt(convergedParameterStd[i] / numberConverged);
}

// print fit results
System.out.println("*Gpufit*");
System.out.println(String.format("Model: %s", model.name()));
System.out.println(String.format("Number of fits: %d", numberFits));
System.out.println(String.format("Fit size: %d x %d", sizeX, sizeX));
System.out.println(String.format("Mean Chi²: %.2f", meanFloatBuffer(fitResult.chiSquares, converged)));
System.out.println(String.format("Mean  number iterations: %.2f", meanIntBuffer(fitResult.numberIterations,
↪ converged)));
System.out.println(String.format("Time: %.2fs", fitResult.fitDuration));
System.out.println(String.format("Ratio converged: %.2f %%", (float) numberConverged / numberFits * 100));
System.out.println(String.format("Ratio max it. exceeded: %.2f %%", (float) numberMaxIterationExceeded /␣
↪numberFits * 100));
System.out.println(String.format("Ratio singular Hessian: %.2f %%", (float) numberSingularHessian /␣
↪numberFits * 100));
System.out.println(String.format("Ratio neg. curvature MLE: %.2f %%", (float) numberNegativeCurvatureMLE /␣
↪numberFits * 100));

System.out.println("\nParameters of 2D Gaussian peak");
for (int i = 0; i < model.numberParameters; i++) {
    System.out.println(String.format("parameter %d, true: %.2f, mean %.2f, std: %.2f", i,␣
↪trueParameters[i], convergedParameterMean[i], convergedParameterStd[i]));
}
```

CHAPTER

NINE

APPENDIX

9.1 Levenberg-Marquardt algorithm

A flowchart of the implementation of the Levenberg-Marquardt algorithm is given in Fig. 9.1.

9.2 Performance comparison to other GPU benchmarks

Using the bundled application (initial release created with CUDA runtime 8.0) to estimate the fitting speed per second of 10 million fits for various CUDA capable graphics cards of various architectures (on different computers with different versions of graphics drivers) we can compare to the results of Passmark G3D. By and large, the results seem to correlate, i.e. a high Passmark G3D score also relates to a high Gpufit fitting speed.

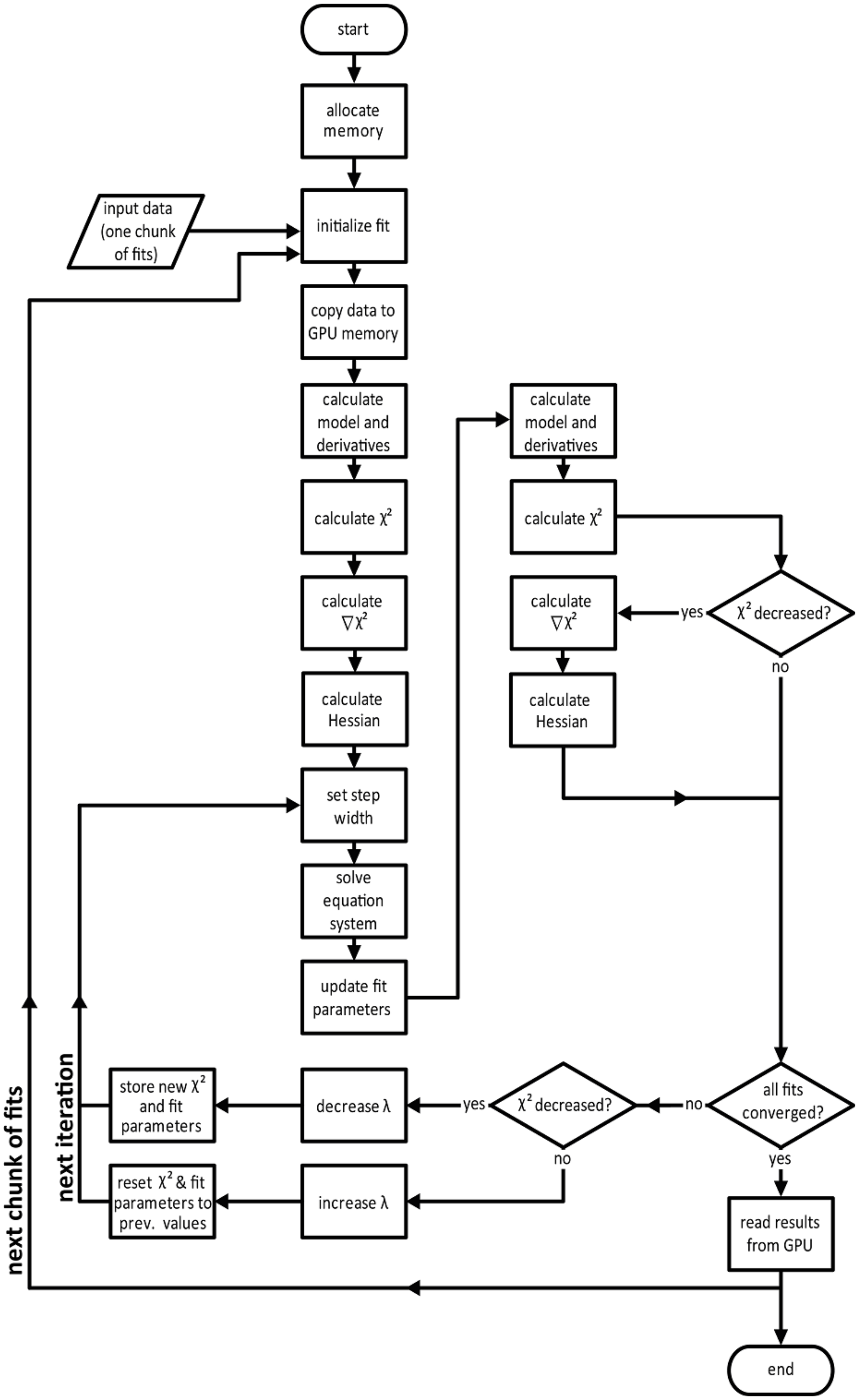

Fig. 9.1: Levenberg-Marquardt algorithm flow as implemented in the Gpufit library.

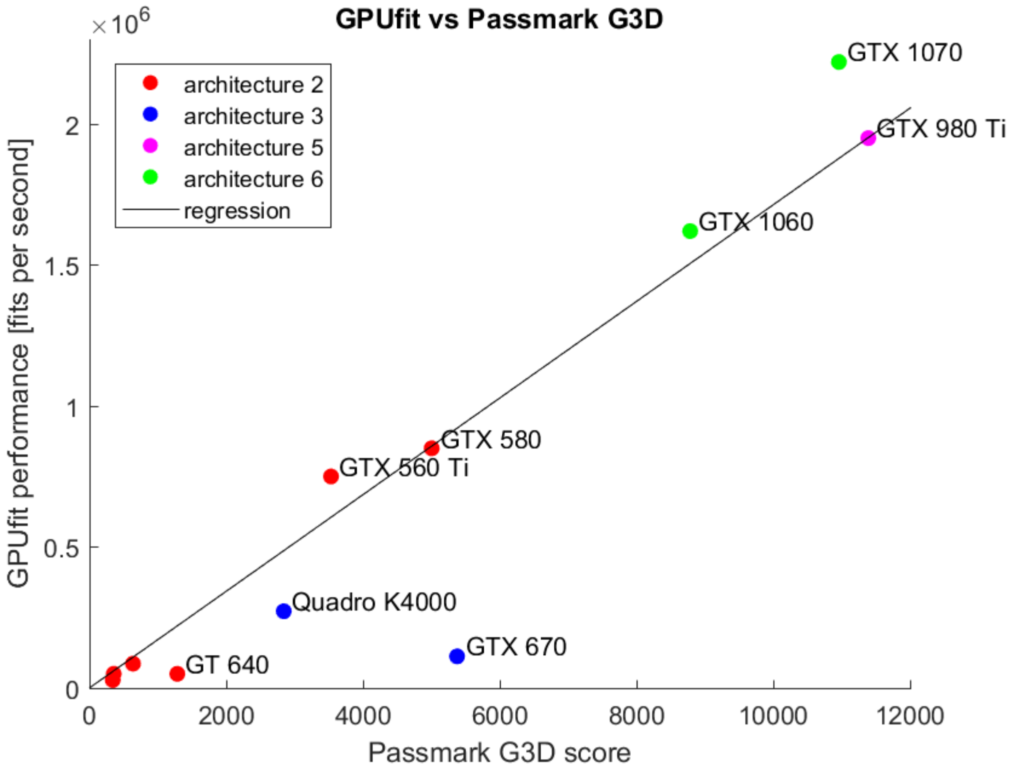

Fig. 9.2: Performance of Gpufit vs Passmark G3D

CHAPTER

TEN

GPUFIT SOFTWARE LICENSE

MIT License

Copyright (c) 2017 Mark Bates, Adrian Przybylski, Björn Thiel, and Jan Keller-Findeisen

Permission is hereby granted, free of charge, to any person obtaining a copy of this software and associated documentation files (the “Software”), to deal in the Software without restriction, including without limitation the rights to use, copy, modify, merge, publish, distribute, sublicense, and/or sell copies of the Software, and to permit persons to whom the Software is furnished to do so, subject to the following conditions:

The above copyright notice and this permission notice shall be included in all copies or substantial portions of the Software.

THE SOFTWARE IS PROVIDED “AS IS”, WITHOUT WARRANTY OF ANY KIND, EXPRESS OR IMPLIED, INCLUDING BUT NOT LIMITED TO THE WARRANTIES OF MERCHANTABILITY, FITNESS FOR A PARTICULAR PURPOSE AND NONINFRINGEMENT. IN NO EVENT SHALL THE AUTHORS OR COPYRIGHT HOLDERS BE LIABLE FOR ANY CLAIM, DAMAGES OR OTHER LIABILITY, WHETHER IN AN ACTION OF CONTRACT, TORT OR OTHERWISE, ARISING FROM, OUT OF OR IN CONNECTION WITH THE SOFTWARE OR THE USE OR OTHER DEALINGS IN THE SOFTWARE.
