# Supplementary material for: Optimal precision and accuracy in 4Pi-STORM using dynamic spline PSF models
Source: Nat Methods. 2022 May 16;19(5):603–12. doi: 10.1038/s41592-022-01465-8 (PMC9119851; doi:10.1038/s41592-022-01465-8)
Supplement: Supplementary file 3 — Supplementary software and data package. [file 41592_2022_1465_MOESM3_ESM.zip › supplementary_data_and_software/code/gpuspline/Gpuspline_1.0.0_Manual.pdf]

Gpuspline Documentation

Release 1.0.0

Adrian Przybylski, Jan Keller-Findeisen, Mark Bates

Oct 13, 2021

CONTENTS

1 Introduction **1**

2 Installation and Testing **2**
2.1 Building from source code 2

3 API description **5**
3.1 C Interfaces 5

4 External bindings **19**
4.1 Python 19
4.2 Matlab 22

5 Model description **27**
5.1 1D Spline model 27
5.2 2D Spline model 27
5.3 3D Spline model 28

CHAPTER

ONE

INTRODUCTION

Gpuspline is a software library for the calculation of multidimensional cubic splines. It calculates spline coefficients and interpolated data values in 1D to 3D.

This manual describes how to install, build and use the Gpuspline library and its external bindings. It includes:

- Instructions for building and installing Gpuspline
- A detailed description of the C interface
- A detailed description of the external bindings to Matlab and Python
- Usage examples for C, Matlab, and Python

The current version of the Gpuspline library is 1.0.0 (homepage).

This manual was compiled on Oct 13, 2021.

CHAPTER

TWO

INSTALLATION AND TESTING

2.1 Building from source code

This section describes how to build Gpuspline from source code. The source code has been build successfully and been tested with the Microsoft Visual Studio compiler (2013 - 2019) on Windows 10 as well as with gcc 9.3 on Ubuntu 20.04.

2.1.1 Prerequisites

The following tools are required in order to build Gpuspline from source.

Required

- CMake 3.11 or later
- A C/C++ Compiler (gcc on Linux, Visual Studio on Windows)

Optional

- MATLAB if building the MATLAB bindings
- Python if building the Python bindings (and for producing Latex and HTML documentation output)
- PDF Latex installation (like Miktex on Windows or texlive-binaries on Linux) if converting the documentation from Latex to PDF

2.1.2 Source code availability

The source code is available in an open repository hosted at Github, at the following URL.

```
https://github.com/gpufit/Gpuspline.git
```

To obtain the code, Git may be used to clone the repository.

2.1.3 Compiler configuration via CMake

CMake is an open-source tool designed to build, test, and package software. It is used to control the software compilation process using compiler independent configuration files, and generate native makefiles and workspaces that can be used in the compiler environment. In this section we provide a simple example of how to use CMake in order to generate the input files for the compiler (e.g. the Visual Studio solution file), which can then be used to compile Gpuspline.

First, identify the directory which contains the Gpuspline source code (for example, on a Windows computer the Gpuspline source code may be stored in *C:\Sources\Gpuspline*). Next, create a build directory outside the source code source directory (e.g. *C:\Sources\Gpuspline-build-64*). Finally, run cmake to configure and generate the compiler input files.

2.1.4 Using the CMake Graphical User Interface

There is a graphical user interface available for CMake, which simplifies the configuration and generation steps. For further details, see Running CMake. The following steps outline how to use the basic features of the CMake GUI.

First, select the source code directory (the top level directory where the Gpuspline source code is located), and the build directory (where the binaries will be built). For this example, the source directory might be *C:\Sources\Gpuspline*, and the build directory might be *C:\Sources\Gpuspline-build-64*.

Next, click the "Configure" button, and select the desired compiler from the drop down list (e.g. Visual Studio 16 2019). Under *Optional platform for Generator*, select the desired architecture (e.g. *x64* to compile 64-bit binaries).

Once configuration is complete, CMake will have automatically found the Matlab installation, and the installation directories will be listed in the *NAME* and *VALUE* columns. If the Matlab installation was not found, the entries in the *VALUE* column can be manually edited.

Next, click on *Generate* to generate the Visual Studio solution files, which will be used to build the Gpuspline package.

2.1.5 Running CMake from the command line

The following commands, executed from the command prompt, assume that the cmake executable (e.g. *C:\Program Files\CMake\bin\cmake.exe*) is automatically found via the PATH environment variable (if not, the full path to cmake.exe must be specified). This example also assumes that the source and build directories have been set up as specified above.

```
cd C:\Sources\Gpuspline-build-64
cmake -G "Visual Studio 16 2019 Win64" C:\Sources\Gpuspline
```

Note that in this example the *-G* flag has been used to specify the 64-bit version of the Visual Studio 14 compiler. This flag should be changed depending on the compiler used, and the desired architecture (e.g. 32- or 64-bit). Further details of the CMake command line arguments can be found here.

2.1.6 Common issues encountered during CMake configuration

Python launcher

Set Python_WORKING_DIRECTORY to a valid directory, it will be added to the Python path.

Matlab launcher

Set Matlab_WORKING_DIRECTORY to a valid directory, it will be added to the Matlab path.

2.1.7 Compiling Gpuspline on Windows

After configuring and generating the solution files using CMake, go to the desired build directory and open Gpuspline.sln using Visual Studio. Select the "Debug" or "Release" build options, as appropriate. Select the build target "ALL_BUILD", and build this target. If the build process completes without errors, the Gpuspline binary files will be created in the corresponding "Debug" or "Release" folders in the build directory.

2.1.8 Compiling Gpuspline on Linux

The following commands can be executed to build Gpuspline on Linux.

```
git clone https://github.com/gpufit/Gpuspline.git Gpuspline
mkdir Gpuspline-build
cd Gpuspline-build
cmake -DCMAKE_BUILD_TYPE=RELEASE ../Gpuspline
make
```

Run the test with

```
./splines_tests
```

To install the Python package

```
cd pyGpuspline/dist
pip install pyGpuspline-X.Y.Z-py2.py3-none-any.whl
```

Finally run the examples with (matplotlib needs a backend, for example pyqt5)

```
pip install matplotlib
pip install pyqt5
python ../../../Gpuspline/examples/python/example_1d_interpolation.py
python ../../../Gpuspline/examples/python/example_2d_resampling.py
```

Optional: Depending on the gcc and the Matlab versions, to run the Matlab package you may need to tell Matlab to use a newer version of the C++ standard library

```
export LD_PRELOAD=/usr/lib/x86_64-linux-gnu/libstdc++.so.6
```

Start Matlab.

```
matlab
```

Then in Matlab add the matlab output directory to the path and execute some examples.

```
addpath('XX/Gpuspline-build/matlab');
cd('XX/Gpuspline/src/examples/matlab');
example_1d_interpolation();
```

CHAPTER

THREE

API DESCRIPTION

The Gpuspline source code compiles to a dynamic-link library (DLL), providing C interfaces. In the sections below, the C interfaces and their arguments are described in detail.

3.1 C Interfaces

The C interfaces are defined in the header file: spline.h.

3.1.1 calculate_coefficients_1d()

This function calculates 1D cubic spline coefficients for intervals between data points of the input data. Every interval is represented by 4 coefficients. The number of spline intervals is one less than the number of data points.

```
int calculate_coefficients_1d(
    REAL * data,
    size_t data_size_x,
    REAL * coefficients);
```

Description of input parameters

data Pointer to data values

type REAL *

length data_size_x

data_size_x Number of data points

type size_t

Description of output parameters

coefficients Calculated spline coefficients, ordered first by spline interval and then by increasing coefficient order (in x).

type REAL *

length 4 * (data_size_x - 1)

3.1.2 calculate_coefficients_2d()

This function calculates 2D cubic spline coefficients for intervals between data points of the input data. Every interval is represented by 16 coefficients. The number of spline intervals is the product of the dimensions of the input data array reduced by 1 in each dimension.

```
int calculate_coefficients_2d(
    REAL * data,
    size_t data_size_x,
    size_t data_size_y,
    REAL * coefficients);
```

Description of input parameters

data Pointer to data values

type REAL *

length data_size_x * data_size_y

data_size_x Data dimension x

type size_t

data_size_y Data dimension y

type size_t

Description of output parameters

coefficients Calculated spline coefficients, ordered first by the spline intervals (in x and then y) and then by increasing orders of the spline coefficients (first in x and then in y).

type REAL *

length 16 * (data_size_x - 1) * (data_size_y - 1)

3.1.3 calculate_coefficients_3d()

This function calculates 3D cubic spline coefficients for intervals between data points of the input data. Every interval is represented by 64 coefficients. The number of spline intervals is the product of the dimensions of the input data array reduced by 1 in each dimension.

```
int calculate_coefficients_3d(
    REAL * data,
    size_t data_size_x,
    size_t data_size_y,
    size_t data_size_z,
    REAL * coefficients);
```

Description of input parameters

data Pointer to data values

type REAL *

length data_size_x * data_size_y * data_size_z

data_size_x Data dimension x

type size_t

data_size_y Data dimension y

type size_t

data_size_z Data dimension z

type size_t

Description of output parameters

coefficients Calculated spline coefficients, ordered first by the spline intervals (in x,then y, then z) and then by increasing orders of the spline coefficients (first in x, then in y, then in z).

type REAL *

length 64 * (data_size_x - 1) * (data_size_y - 1) * (data_size_z - 1)

3.1.4 interpolate_1d()

This function performs a 1D data interpolation based on the cubic spline interpolation method.

```
int interpolate_1d(
    REAL * data,
    size_t data_size_x,
    size_t new_size_x,
    REAL * x_values,
    REAL * interpolated_data);
```

Description of input parameters

data Pointer to data values

type REAL *

length data_size_x

data_size_x number of input data points

type size_t

new_size_x number of output data points

type size_t

x_values Pointer to independent variable values, starting with 0 (1st spline interval, covering the distance between 1st and 2nd data point) and going to N-1 for N data points.

type REAL *

length new_size_x

Description of output parameters

interpolated_data Pointer to output data values. Outside of the valid spline interval positions, the values returned will be constant (closest data values).

type REAL *

length new_size_x

3.1.5 interpolate_2d()

This function performs a 2D data interpolation based on the cubic spline interpolation method.

```
int interpolate_2d(
    REAL * data,
    size_t data_size_x,
    size_t data_size_y,
    size_t new_size_x,
    size_t new_size_y,
    REAL * x_values,
    REAL * y_values,
    REAL * interpolated_data);
```

Description of input parameters

data Pointer to data values

> **type** REAL *
>
> **length** data_size_x * data_size_y

data_size_x Input data dimension x

> **type** size_t

data_size_y Input data dimension y

> **type** size_t

new_size_x Output data dimension x

> **type** size_t

new_size_y Output data dimension y

> **type** size_t

x_values Pointer to independent variable x values, starting with 0 (1st spline interval, covering the distance between 1st and 2nd data point) and going to N-1 for N data points in x.

> **type** REAL *
>
> **length** new_size_x

y_values Pointer to independent variable y values, starting with 0 (1st spline interval, covering the distance between 1st and 2nd data point) and going to M-1 for M data points in y.

> **type** REAL *
>
> **length** new_size_y

Description of output parameters

interpolated_data Pointer to output data values. Outside of the valid spline interval positions, the values returned will be constant (closest data values).

> **type** REAL *
>
> **length** new_size_x * new_size_y

3.1.6 interpolate_3d()

This function performs a 3D data interpolation based on the cubic spline interpolation method.

```
int interpolate_3d(
    REAL * data,
    size_t data_size_x,
    size_t data_size_y,
    size_t data_size_z,
    size_t new_size_x,
    size_t new_size_y,
    size_t new_size_z,
    REAL * x_values,
    REAL * y_values,
    REAL * z_values,
    REAL * interpolated_data);
```

Description of input parameters

data Pointer to data values

type REAL *

length data_size_x * data_size_y * data_size_z

data_size_x Input data dimension x

type size_t

data_size_y Input data dimension y

type size_t

data_size_z Input data dimension z

type size_t

new_size_x Output data dimension x

type size_t

new_size_y Output data dimension y

type size_t

new_size_z Output data dimension z

type size_t

x_values Pointer to independent variable x values, starting with 0 (1st spline interval, covering the distance between 1st and 2nd data point) and going to N-1 for N data points in x.

type REAL *

length new_size_x

y_values Pointer to independent variable y values, starting with 0 (1st spline interval, covering the distance between 1st and 2nd data point) and going to M-1 for M data points in y.

type REAL *

length new_size_y

z_values Pointer to independent variable z values, starting with 0 (1st spline interval, covering the distance between 1st and 2nd data point) and going to K-1 for K data points in z.

type REAL *

length new_size_z

Description of output parameters

interpolated_data Pointer to output data values. Outside of the valid spline interval positions, the values returned will be constant (closest data values).

type REAL *

length new_size_x * new_size_y * new_size_z

3.1.7 calculate_values_1d()

This function calculates 1D function values based on provided spline coefficients and independent variable values.

```
int calculate_values_1d(
    REAL * coefficients,
    size_t const n_intervals_x,
    size_t const values_size_x,
    REAL * x_values,
    REAL * spline_values);
```

Description of input parameters

coefficients Pointer to spline coefficients

type REAL *

length 4 * n_intervals_x

n_intervals_x Number of spline intervals

type size_t

values_size_x Number of output data points

type size_t

x_values Pointer to independent variable values, starting with 0 (1st spline interval, covering the distance between 1st and 2nd data point) and going to N-1 for N data points in x.

type REAL *

length values_size_x

Description of output parameters

spline_values Pointer to output data values. Outside of the valid spline interval positions, the values returned will be constant (closest data values).

type REAL *

length values_size_x

3.1.8 calculate_values_2d()

This function calculates function values based on provided spline coefficients and independent variable values.

```
int calculate_values_2d(
    REAL * coefficients,
    size_t const n_intervals_x,
    size_t const n_intervals_y,
    size_t const values_size_x,
    size_t const values_size_y,
    REAL * x_values,
```

(continues on next page)

(continued from previous page)

```
    REAL * y_values,
    REAL * spline_values);
```

Description of input parameters

coefficients Pointer to spline coefficients

type REAL *

length 16 * n_intervals_x * n_intervals_y

n_intervals_x Number of spline intervals in x

type size_t

n_intervals_y Number of spline intervals in y

type size_t

values_size_x Output data dimension x

type size_t

values_size_y Output data dimension y

type size_t

x_values Pointer to independent variable x values, starting with 0 (1st spline interval, covering the distance between 1st and 2nd data point) and going to N-1 for N data points in x.

type REAL *

length values_size_x

y_values Pointer to independent variable y values, starting with 0 (1st spline interval, covering the distance between 1st and 2nd data point) and going to M-1 for M data points in y.

type REAL *

length values_size_y

Description of output parameters

spline_values Pointer to output data values. Outside of the valid spline interval positions, the values returned will be constant (closest data values).

type REAL *

length values_size_x * values_size_y

3.1.9 calculate_values_3d()

This function calculates function values based on provided spline coefficients and independent variable values.

```
int calculate_values_3d(
    REAL * coefficients,
    size_t const n_intervals_x,
    size_t const n_intervals_y,
    size_t const n_intervals_z,
    size_t const values_size_x,
    size_t const values_size_y,
    size_t const values_size_z,
    REAL * x_values,
    REAL * y_values,
    REAL * z_values,
    REAL * spline_values);
```

Description of input parameters

coefficients Pointer to spline coefficients

type REAL *

length 64 * n_intervals_x * n_intervals_y * n_intervals_z

n_intervals_x Number of spline intervals in x

type size_t

n_intervals_y Number of spline intervals in y

type size_t

n_intervals_z Number of spline intervals in z

type size_t

values_size_x Output data dimension x

type size_t

values_size_y Output data dimension y

type size_t

values_size_y Output data dimension z

type size_t

x_values Pointer to independent variable x values, starting with 0 (1st spline interval, covering the distance between 1st and 2nd data point) and going to N-1 for N data points in x.

type REAL *

length values_size_x

y_values Pointer to independent variable y values, starting with 0 (1st spline interval, covering the distance between 1st and 2nd data point) and going to M-1 for M data points in y.

type REAL *

length values_size_y

z_values Pointer to independent variable z values, starting with 0 (1st spline interval, covering the distance between 1st and 2nd data point) and going to K-1 for K data points in z.

type REAL *

length values_size_z

Description of output parameters

spline_values Pointer to output data values. Outside of the valid spline interval positions, the values returned will be constant (closest data values).

type REAL *

length values_size_x * values_size_y * values_size_z

3.1.10 calculate_coefficients_1d_portable()

This function is a simple wrapper around the `calculate_coefficients_1d()` function, providing an alternative means of passing the function parameters.

```
int calculate_coefficients_1d_portable(int argc, void *argv[]);
```

Description of parameters

argc The length of the argv pointer array

argv Array of pointers to *calculate_coefficients_1d* parameters, as defined above. For reference, the type of each element of the *argv* array is listed below.

argv[0] Data

type REAL *

argv[1] Number of data points

type size_t *

argv[2] Spline coefficients

type REAL *

3.1.11 calculate_coefficients_2d_portable()

This function is a simple wrapper around the `calculate_coefficients_2d()` function, providing an alternative means of passing the function parameters.

```
int calculate_coefficients_2d_portable(int argc, void *argv[]);
```

Description of parameters

argc The length of the argv pointer array

argv Array of pointers to *calculate_coefficients_2d* parameters, as defined above. For reference, the type of each element of the *argv* array is listed below.

argv[0] Data

type REAL *

argv[1] Data dimension x

type size_t *

argv[2] Data dimension y

type size_t *

argv[3] Spline coefficients

type REAL *

3.1.12 calculate_coefficients_3d_portable()

This function is a simple wrapper around the `calculate_coefficients_3d()` function, providing an alternative means of passing the function parameters.

```
int calculate_coefficients_3d_portable(int argc, void *argv[]);
```

Description of parameters

argc The length of the argv pointer array

argv Array of pointers to *calculate_coefficients_3d* parameters, as defined above. For reference, the type of each element of the *argv* array is listed below.

- **argv[0]** Data
  - **type** REAL *
- **argv[1]** Data dimension x
  - **type** size_t *
- **argv[2]** Data dimension y
  - **type** size_t *
- **argv[3]** Data dimension z
  - **type** size_t *
- **argv[4]** Spline coefficients
  - **type** REAL *

3.1.13 interpolate_1d_portable()

This function is a simple wrapper around the `interpolate_1d()` function, providing an alternative means of passing the function parameters.

```
int interpolate_1d_portable(int argc, void *argv[]);
```

Description of parameters

argc The length of the argv pointer array

argv Array of pointers to *interpolate_1d* parameters, as defined above. For reference, the type of each element of the *argv* array is listed below.

- **argv[0]** Input data
  - **type** REAL *
- **argv[1]** Input number of data points
  - **type** size_t *
- **argv[2]** Output number of data points
  - **type** size_t *
- **argv[3]** Independent variable values
  - **type** REAL *
- **argv[4]** Output data
  - **type** REAL *

3.1.14 interpolate_2d_portable()

This function is a simple wrapper around the `interpolate_2d()` function, providing an alternative means of passing the function parameters.

```
int interpolate_2d_portable(int argc, void *argv[]);
```

Description of parameters

argc The length of the argv pointer array

argv Array of pointers to *interpolate_2d* parameters, as defined above. For reference, the type of each element of the *argv* array is listed below.

argv[0] Input data

type REAL *

argv[1] Input data dimension x

type size_t *

argv[2] Input data dimension y

type size_t *

argv[3] Output data dimension x

type size_t *

argv[4] Output data dimension y

type size_t *

argv[5] Independent variable x values

type REAL *

argv[6] Independent variable y values

type REAL *

argv[7] Output data

type REAL *

3.1.15 interpolate_3d_portable()

This function is a simple wrapper around the `interpolate_3d()` function, providing an alternative means of passing the function parameters.

```
int interpolate_3d_portable(int argc, void *argv[]);
```

Description of parameters

argc The length of the argv pointer array

argv Array of pointers to *interpolate_3d* parameters, as defined above. For reference, the type of each element of the *argv* array is listed below.

argv[0] Input data

type REAL *

argv[1] Input data dimension x

type size_t *

argv[2] Input data dimension y

type size_t *

argv[3] Input data dimension z

type size_t *

argv[4] Output data dimension x

type size_t *

argv[5] Output data dimension y

type size_t *

argv[6] Output data dimension z

type size_t *

argv[7] Independent variable x values

type REAL *

argv[8] Independent variable y values

type REAL *

argv[9] Independent variable z values

type REAL *

argv[10] Output data

type REAL *

3.1.16 calculate_values_1d_portable()

This function is a simple wrapper around the `calculate_values_1d()` function, providing an alternative means of passing the function parameters.

```
int calculate_values_1d_portable(int argc, void *argv[]);
```

Description of parameters

argc The length of the argv pointer array

argv Array of pointers to *calculate_values_1d* parameters, as defined above. For reference, the type of each element of the *argv* array is listed below.

argv[0] Spline coefficients

type REAL *

argv[1] Number of spline intervals

type size_t *

argv[2] Number of output data points

type size_t *

argv[3] Independent variable values

type REAL *

argv[4] Output data values

type REAL *

3.1.17 calculate_values_2d_portable()

This function is a simple wrapper around the `calculate_values_2d()` function, providing an alternative means of passing the function parameters.

```
int calculate_values_2d_portable(int argc, void *argv[]);
```

Description of parameters

argc The length of the argv pointer array

argv Array of pointers to *calculate_values_2d* parameters, as defined above. For reference, the type of each element of the *argv* array is listed below.

argv[0] Spline coefficients

type REAL *

argv[1] Number of spline intervals in x

type size_t *

argv[2] Number of spline intervals in y

type size_t *

argv[3] Output data dimension x

type size_t *

argv[4] Output data dimension y

type size_t *

argv[5] Independent variable x values

type REAL *

argv[6] Independent variable y values

type REAL *

argv[7] Output data values

type REAL *

3.1.18 calculate_values_3d_portable()

This function is a simple wrapper around the `calculate_values_3d()` function, providing an alternative means of passing the function parameters.

```
int calculate_values_3d_portable(int argc, void *argv[]);
```

Description of parameters

argc The length of the argv pointer array

argv Array of pointers to *calculate_values_3d* parameters, as defined above. For reference, the type of each element of the *argv* array is listed below.

argv[0] Spline coefficients

type REAL *

argv[1] Number of spline intervals in x

type size_t *

argv[2] Number of spline intervals in y

type size_t *

argv[3] Number of spline intervals in z

type size_t *

argv[4] Output data dimension x

type size_t *

argv[5] Output data dimension y

type size_t *

argv[6] Output data dimension z

type size_t *

argv[7] Independent variable x values

type REAL *

argv[8] Independent variable y values

type REAL *

argv[9] Independent variable z values

type REAL *

argv[10] Output data values

type REAL *

CHAPTER

FOUR

EXTERNAL BINDINGS

This sections describes the Gpuspline bindings to other programming languages. The bindings to Python and Matlab aim to emulate the *C Interfaces* (page 5) as closely as possible.

4.1 Python

The Python binding for Gpuspline consists of a Python package pyGpuspline that provides various functions that call the C interface of the Gpuspline library. In general the routines expect data as NumPy arrays.

4.1.1 Installation

Wheel files for Python (x64) on Windows are included in the binary package. NumPy is required.

Install the wheel file with

```
pip install --no-index --find-links=LocalPathToWheelFile pyGpuspline
```

4.1.2 Python Interface

The Python interface is a thin wrapper around the C interface. Please see the API documentation of the *C Interfaces* (page 5) for more details on the interpretation of input and output parameters.

spline_coefficients

The signature of the spline_coefficients method is

```
def spline_coefficients(data)
```

The data must be a 1-3D NumPy array of data type single. This method is equivalent to call the C interface functions calculate_coefficients_Xd. The return value is a NumPy array of size 4^d (d=dimension of data) times product of number of pixels in each direction minus one.

spline_interpolate

The signature of the spline_interpolate method is

```
def spline_interpolate(data, x, y=None, z=None):
```

The data must be a 1-3D NumPy array of data type single. This method is equivalent to call the C interface functions interpolate_Xd. In case of a 1D data array only x values should be specified, in case of a 2D array x and y values and for a 3D data array x, y and z values.

The output is the interpolated data, a NumPy array with product of elements in x times elements in y times elements in z entries.

spline_values

The signature of the spline_values method is

```
def spline_values(coefficients, x, y=None, z=None):
```

The coefficients are a (d+1) dimensional NumPy array of type single (d=dimension of original data) describing the spline associated with the data. In the first dimension with 4^d entries are the coefficients of a single spline interval. The spline intervals for each data pixel are stored from the second dimension on. For a 1D spline, only specify x, for a 2D spline only x and y and for a 3D spline only x, y and z. The output is a NumPy array holding the interpolated values of the data represented by the splines at the positions specified by x, y and z. This method is equivalent to call functions calculate_values_Xd in the C interface.

4.1.3 Python Examples

1D interpolation example

An example for interpolating data points calling a cubic spline interpolation routine implemented in C.1D data is upsampled, cut, stretched and shifted. The example can be found at example_1d_interpolation.py.

```
"""
Example of the Python binding of the Gpuspline library for the
calculation of multidimensional cubic splines.
https://github.com/gpufit/Gpuspline
https://gpuspline.readthedocs.io/en/latest/bindings.html#python

Interpolates 1D data. The data is upsampled, cut, stretched and shifted.

Requires pyGpuspline, Numpy and Matplotlib
"""

import numpy as np
from matplotlib import pyplot as plt
import pygpuspline.gpuspline as gs

if __name__ == '__main__':
    # input data
    y = np.array([0, 0, 0.2, 1, 1.1, 1.3, 2, 2.5, 3, 4, 4.25, 4, 3, 2.5, 2, 1.3, 1.1, 1, 0.2, 0, 0], np.
↪float32)
    x = np.arange(y.size)
    center = x[-1] / 2

    # interpolation parameters
    edge = 1.4
    width = 1.1
    shift = 1.2
    sampling_factor = 0.5

    # interpolation
    xq = np.arange(x[0], x[-1], sampling_factor, np.float32)
```

(continues on next page)

(continued from previous page)

```
    xq = xq[np.logical_and(xq >= edge, xq <= np.amax(xq) - edge)]
    xq /= width
    xq += center * (1 - 1 / width) - shift
    yq = gs.spline_interpolate(y, xq)  # call to the spline library

    # show result
    fig, ax = plt.subplots()
    ax.plot(x, y, color='blue', label='original')
    ax.plot(xq + shift, yq, color='red', marker='x', label='interpolated')
    ax.grid()
    ax.set_xlim(0, 20)
    ax.set_ylim(0, 1.1 * np.amax(y))
    ax.legend()
    plt.show()
```

2D resampling and shifting example

The example can be found at example_2d_resampling.py.

```
"""
Example of the Matlab binding of the Gpuspline library for the
calculation of multidimensional cubic splines.
https://github.com/gpufit/Gpuspline
https://gpuspline.readthedocs.io/en/latest/bindings.html#python

2D data is interpolated (up- and downsampled and shifted).

Requires pyGpuspline, Numpy and Matplotlib
"""

import numpy as np
from matplotlib import pyplot as plt
import pygpuspline.gpuspline as gs


def calculate_psf(x, y, p):
    """
    Calculates an elliptic 2D Gaussian peak function.
    """
    sx = p[3] - 0.2
    sy = p[3] + 0.2

    psf = p[0] * np.exp(-0.5 * (((x - p[1]) / sx) ** 2 + ((y - p[2]) / sy) ** 2)) + p[4]

    return psf


if __name__ == '__main__':
    # PSF size
    size_x = 10
    size_y = 20

    # derived values
    x = np.arange(size_x, dtype=np.float32).reshape((size_x, 1))
    y = np.arange(size_y, dtype=np.float32).reshape((1, size_y))

    x_up = np.arange(size_x, step=0.1, dtype=np.float32)
    y_up = np.arange(size_y, step=0.1, dtype=np.float32)

    x_down = np.arange(size_x, step=2, dtype=np.float32)
    y_down = np.arange(size_y, step=2, dtype=np.float32)

    x_shift = x - 1.3
    y_shift = y + 2.7

    # PSF parameters
    psf_parameters = (100, (size_x - 1) / 2, (size_y - 1) / 2, 3, 10)
```

(continues on next page)

(continued from previous page)

```
# calculate PSF
psf = calculate_psf(x, y, psf_parameters)

# calculate spline coefficients
coefficients = gs.spline_coefficients(psf)  # call to spline library

# generate upsampled PSF
psf_up = gs.spline_values(coefficients, x_up, y_up)  # call to spline library

# generate downsampled PSF
psf_down = gs.spline_values(coefficients, x_down, y_down)  # call to spline library

# generate shifted PSF
psf_shift = gs.spline_values(coefficients, x_shift, y_shift)  # call to spline library

# display results
fig, axs = plt.subplots(2, 2)
fig.tight_layout()
axs = axs.flat
axs[0].imshow(psf, cmap='hot')
axs[0].set_title('Original data')
axs[1].imshow(psf_up, cmap='hot')
axs[1].set_title('Upsampled')
axs[2].imshow(psf_down, cmap='hot')
axs[2].set_title('Downsampled')
axs[3].imshow(psf_shift, cmap='hot')
axs[3].set_title('Shifted')
plt.show()
```

4.2 Matlab

The Matlab binding for Gpuspline consists of Matlab scripts (spline_coefficients.m, spline_values.m, spline_interpolate.m). These scripts check the input data and call the *C Interfaces* (page 5) of the Gpuspline library, via compiled .mex files. Please note, that before using the Matlab binding, the path to the .m and .mex files must be added to the Matlab path.

4.2.1 Matlab Interfaces

spline_coefficients

The data dimensions are deduced from the dimensions of the input data.

The signature of the spline_coefficients function is

```
function [coefficients, time] = spline_coefficients(data)
```

Input parameters

data Data 1D, 2D or 3D matrix of data type single.

Output parameters

coefficients Cubic spline coefficients 2D, 3D or 4D matrix of data type single of size: [N_c, N_x, N_y, N_z] ,where N_c represents the number of coefficients per spline interval and N_x, N_y, N_z the number of spline intervals in x, y, z.

time Execution time of call to spline_coefficientsMex in seconds.

Errors are raised if checks on parameters fail or if the execution of the function fails.

spline_values

The spline dimensions are deduced from the dimensions of the input data and the number of input arguments. This function calculates function values based on cubic spline coefficients. Optionally it calculates values for multiple splines, if the 5th dimension of the input spline coefficients is greater than 1.

The signature of the spline_values function is

```
function [coefficients, time] = spline_values(coefficients, x, y, z)
```

Input parameters

coeficients Cubic spline coefficients 2D, 3D, 4D or 5D matrix of data type single.

dimension1 Number of spline coefficients per spline interval depending on the number of dimensions of the spline (4, 16 or 64)

dimension2 Number of spline intervals in x

dimension3 Number of spline intervals in y

dimension4 Number of spline intervals in z

dimension5 Number of splines/channels

x Independent variable x values

y Independent variable y values (optional)

z Independent variable z values (optional)

y and z parameter are optional (for 2D/3D data)

Output parameters

values Output values 1D, 2D, 3D or 4D matrix of data type single of size: [N_x, N_y, N_z, N_{ch}] ,where N_x, N_y, N_z represent the number of output data points in x, y, z and N_{ch} the number of channels.

time Execution time of call to spline_valuesMex in seconds.

Errors are raised if checks on parameters fail or if the execution of the function fails.

spline_interpolate

The data dimensions are deduced from the number of input arguments.

The signature of the spline_interpolate function is

```
function [interpolated_data, time] = spline_interpolate(data, x, y, z)
```

Input parameters

data Input data values 1D, 2D or 3D matrix of data type single.

x Independent variable x values 1D matrix of data type single.

y Independent variable y values (optional) 1D matrix of data type single.

z Independent variable z values (optional) 1D matrix of data type single.

y and z parameter are optional (for 2D/3D interpolation)

Output parameters

values Interpolated data values 1D, 2D or 3D matrix of data type single of size: [N_x, N_y, N_z] ,where N_x, N_y, N_z represent the number of output data points in x, y, z.

time Execution time of call to spline_interpolateMex in seconds.

Errors are raised if checks on parameters fail or if the execution of the function fails.

4.2.2 Matlab Examples

1D interpolation example

An example for interpolating data points calling a cubic spline interpolation routine implemented in C.1D data is upsampled, cut, stretched and shifted. The example can be found at example_1d_interpolation.m.

2D resampling example

Example can be found at example_2d_resampling.m.

```
function example_2d_resampling()
% Example of the Matlab binding of the Gpuspline library for the
% calculation of multidimensional cubic splines.
% https://github.com/gpufit/Gpuspline
%
% 2D data is interpolated (up- and downsampled).
% https://gpuspline.readthedocs.io/en/latest/bindings.html#matlab

%% psf size
size_x = 15;
size_y = 20;

%% derived values
x = single(0 : size_x - 1)';
y = single(0 : size_y - 1);

x_up = single(0 : 0.1 : size_x - 1)';
y_up = single(0 : 0.1 : size_y - 1)';

x_down = single(0 : 2 : size_x - 1)';
y_down = single(0 : 2 : size_y - 1)';

%% PSF parameters
psf_parameters = single([100, (size_x-1)/2, (size_y-1)/2, 3, 10]);

%% calculate PSF
psf = calculate_psf(x, y, psf_parameters);

%% calculate spline coefficients
coefficients = spline_coefficients(psf);

%% generate upsampled psf
psf_up = spline_values(coefficients, x_up, y_up);

%% generate downsampled psf
psf_down = spline_values(coefficients, x_down, y_down);

%% figure
figure;
subplot(131); imagesc(x, y, psf);
axis image; title('Original data');
subplot(132); imagesc(x_up, y_up, psf_up);
axis image; title('Upsampled');
subplot(133); imagesc(x_down, y_down, psf_down);
axis image; title('Downsampled');
colormap('hot');

end

function psf = calculate_psf(x, y, p)
% PSF consists of an elliptic 2D Gaussian

% p(1) - amplitude
% p(2) - center x
% p(3) - center y
% p(4) - Standard deviation
% p(5) - constant background
assert(nargin == 3);
```

(continues on next page)

(continued from previous page)

```
sx = p(4) - 0.2;
sy = p(4) + 0.2;

arg_ex = exp(-1/2*((x-p(2))/sx).^2-1/2*((y-p(3))/sy).^2);

psf = p(1) .* arg_ex + p(5); % scale with amplitude and background

end
```

example_2d_shift()

Example can be found at example_2d_shift.m.

```
function example_2d_shift()
% Example of the Matlab binding of the Gpuspline library for the
% calculation of multidimensional cubic splines.
% https://github.com/gpufit/Gpuspline
%
% 2D data is interpolated (shifted).
% https://gpuspline.readthedocs.io/en/latest/bindings.html#matlab

%% psf size
size_x = 20;
size_y = 30;

%% derived values
x = single(0 : size_x - 1)';
y = single(0 : size_y - 1);

x_shifted = x - 1.3;
y_shifted = y + 2.7;

%% PSF parameters
psf_parameters = single([100, (size_x-1)/2, (size_y-1)/2, 2, 10]);

%% calculate PSF
psf = calculate_psf(x, y, psf_parameters);

%% calculate spline coefficients
coefficients = spline_coefficients(psf);

%% generate upsampled psf
psf_shifted = spline_values(coefficients, x_shifted, y_shifted);

%% figure
figure;
subplot(121); imagesc(x, y, psf);
axis image; title('Original');
subplot(122); imagesc(x_shifted, y_shifted, psf_shifted);
axis image; title('Shifted');
colormap('hot');

end

function psf = calculate_psf(x, y, p)
% PSF consists of an elliptic 2D Gaussian

% p(1) - amplitude
% p(2) - center x
% p(3) - center y
% p(4) - Standard deviation
% p(5) - constant background
assert(nargin == 3);

sx = p(4) - 0.2;
sy = p(4) + 0.2;
```

(continues on next page)

(continued from previous page)

```
arg_ex = exp(-1/2*((x-p(2))/sx).^2-1/2*((y-p(3))/sy).^2);

psf = p(1) .* arg_ex + p(5); % scale with amplitude and background

end
```

CHAPTER

FIVE

MODEL DESCRIPTION

From a given set of d-dimensional (d=1-3) data points, a cubic spline representation is calculated with 4^d coefficients per d-dimensional data interval. The spline model function will take exactly the value of the data points and the end condition is that 1st and 2nd derivative of the spline model function is zero at the end points (and outside the spline will be extrapolated by constant values, i.e. the nearest data values). As reference the Wolfram Mathworld page on Cubic splines as well as the Python implementation of cubic splines by the ZhuangLab were used.

The cubic spline representation uses the logical spacing of the data (i.e. the interval lengths are all one) with indexing starting at 0. Please take care to translate the spline interval coordinates to your real x,y,z coordinates yourself. In the following the spline model functions for d=1-3 dimensions is given explicitly. The spline model is fully characterized by the set of spline coefficients $S_{.,.}$.

5.1 1D Spline model

The 1D spline model with coefficients $S_{i,m}$ with i specifying the spline interval and m (=0-3) specifying the polynomial order is given by:

$$f_S(x) = \sum_{m=0}^{3} S_{i,m} \left(\frac{x - t_i}{\Delta t_i}\right)^m$$

$$t_i \leq x \leq t_{i+1}, \Delta t_i = t_{i+1} - t_i$$

Each interval is represented by 4 coefficients $S_{i,:}$. The positions t_i specify the left borders of the spline interval i and Δt_i is its size. Here t_i goes from 0 to N-1 for N data points and Δt_i equals 1.

5.2 2D Spline model

The 2D spline model with coefficients $S_{i,j,m,n}$ with (i, j) specifying the spline interval in 2D and (m, n) (=0-3) specifying the polynomial orders in x and y is given by

$$f_S(x, y) = \sum_{m=0}^{3}\sum_{n=0}^{3} S_{i,j,m,n} \left(\frac{x - t_i}{\Delta t}\right)^m \left(\frac{y - u_j}{\Delta u}\right)^n$$

$$t_i \leq x \leq t_{i+1}, \Delta t = t_{i+1} - t_i$$

$$u_j \leq y \leq u_{j+1}, \Delta u = u_{j+1} - u_j$$

Each interval is represented by 16 coefficients $S_{i,j,:,:}$. The positions (t_i, u_j) specify the left borders of the spline interval (i, j) and $(\Delta t_i, \Delta u_j)$ is its size in x and y. Here (t_i, u_j) goes from (0,0) to (N-1, M-1) for NxM data points and $(\Delta t_i, \Delta u_j)$ equals (1,1).

5.3 3D Spline model

The 3D spline model with coefficients $S_{i,j,k,m,n,o}$ with (i, j, k) specifying the spline interval in 3D and (m, n, o) (=0-3) specifying the polynomial orders in x, y and z is given by

$$f_S(x, y, z) = \sum_{m=0}^{3} \sum_{n=0}^{3} \sum_{o=0}^{3} S_{i,j,k,m,n,o} \left(\frac{x - t_i}{\Delta t}\right)^m \left(\frac{y - u_j}{\Delta u}\right)^n \left(\frac{z - v_k}{\Delta v}\right)^o$$

$$t_i \leq x \leq t_{i+1}, \Delta t = t_{i+1} - t_i$$

$$u_j \leq y \leq u_{j+1}, \Delta u = u_{j+1} - u_j$$

$$v_k \leq z \leq v_{k+1}, \Delta v = v_{k+1} - v_k$$

Each interval is represented by 64 coefficients $S_{i,j,k,:,:,:}$. The positions (t_i, u_j, v_k) specify the left borders of the spline interval (i, j, k) and $(\Delta t_i, \Delta u_j, \Delta v_k)$ is its size in x, y and z. Here (t_i, u_j, v_k) goes from (0,0,0) to (N-1, M-1, K-1) for NxMxK data points and $(\Delta t_i, \Delta u_j, \Delta v_k)$ equals (1,1,1).
