# Supplementary material for: Optimal precision and accuracy in 4Pi-STORM using dynamic spline PSF models
Source: Nat Methods. 2022 May 16;19(5):603–12. doi: 10.1038/s41592-022-01465-8 (PMC9119851; doi:10.1038/s41592-022-01465-8)
Supplement: Supplementary file 4 — Supplementary mechanical drawings. [file 41592_2022_1465_MOESM4_ESM.zip › supplementary_mechanical_drawings/README.pdf]

Optimal Precision and Accuracy in 4Pi-STORM using Dynamic Spline PSF Models

Mark Bates[1,2*], Jan Keller-Findeisen[1], Adrian Przybylski[1], Andreas Hüper[1], Till Stephan[1,3], Peter Ilgen[1,3], Angel R. Cereceda Delgado[1,4], Elisa D'Este[5], Alexander Egner[2], Stefan Jakobs[1,3], Steffen J. Sahl[1], and Stefan W. Hell[1,4,*]

[1]Department of NanoBiophotonics, Max Planck Institute for Biophysical Chemistry, 37077 Göttingen, Germany
[2]Department of Optical Nanoscopy, Institute for NanoPhotonics, 37077 Göttingen, Germany
[3]Clinic of Neurology, University Medical Center Göttingen, 37075 Göttingen, Germany
[4]Department of Optical Nanoscopy, Max Planck Institute for Medical Research, 69120 Heidelberg, Germany
[5]Optical Microscopy Facility, Max Planck Institute for Medical Research, 69120 Heidelberg, Germany

* To whom correspondence should be addressed.
mark.bates@mpibpc.mpg.de, stefan.hell@mpibpc.mpg.de

Description of 4Pi-STORM optical setup mechanical drawings

Mechanical drawings for three custom-built components are included as supplementary data files with the manuscript. These parts are the fixed objective lens mount, the movable objective lens mount, and the sample stage. The files are provided in STEP format, which is accessible from many CAD software environments.

The authors note that the files are intended to serve as a design guideline, rather than ready-to-build parts. Our microscope was built in stages, and many parts were modified during fabrication to correct minor design flaws etc. Hence, interested readers are encouraged to use these CAD files as advanced starting points for designing their own system, and to check in detail the mechanical tolerances of the parts shown in the drawings.

The three components, (i) fixed objective mount, (ii) movable objective mount, and (iii) sample stage, are included in a single file: **storm_4pi_mechanical_drawings.stp**.

Notes:

1. All custom parts were constructed from Aluminum, with the exception of the following: sample mounting disc (stainless steel), fixed objective mounting tube (stainless steel), movable objective mounting tube (brass), mounting assembly supporting the three-axis piezo stage (stainless steel). All aluminum parts were anodized prior to assembly.
2. The X- and Y- axis manual micrometer screws shown in the drawing are not present in the real microscope. In place of these parts, we used Thorlabs Z825B 25mm motorized actuators, which connect to the stage via the same clamp mount as the micrometer screws.

License:

The included hardware designs are released under the terms of the CERN Open Hardware License Version 2 - Strongly Reciprocal. Further information regarding the license terms can be found online at https://ohwr.org/cernohl.
